# Pathogen‐induced inflammation is attenuated by the iminosugar M*O*N‐DNJ via modulation of the unfolded protein response

**DOI:** 10.1111/imm.13393

**Published:** 2021-08-01

**Authors:** Andrew C. Sayce, Fernando O. Martinez, Beatrice E. Tyrrell, Nilanka Perera, Michelle L. Hill, Raymond A. Dwek, Joanna L. Miller, Nicole Zitzmann

**Affiliations:** ^1^ Oxford Glycobiology Institute Department of Biochemistry University of Oxford Oxford UK; ^2^ Vanderbilt University School of Medicine Vanderbilt University Nashville Tennessee USA; ^3^ School of Biosciences and Medicine University of Surrey Guildford UK; ^4^ Faculty of Medical Sciences University of Sri Jayewardenepura Gangodawila Nugegoda Sri Lanka

**Keywords:** dengue virus, iminosugar, inflammation, sepsis, unfolded protein response

## Abstract

Sepsis is a life‐threatening condition involving a dysregulated immune response to infectious agents that cause injury to host tissues and organs. Current treatments are limited to early administration of antibiotics and supportive care. While appealing, the strategy of targeted inhibition of individual molecules in the inflammatory cascade has not proved beneficial. Non‐targeted, systemic immunosuppression with steroids has shown limited efficacy and raises concern for secondary infection. Iminosugars are a class of small molecule glycomimetics with distinct inhibition profiles for glycan processing enzymes based on stereochemistry. Inhibition of host endoplasmic reticulum resident glycoprotein processing enzymes has demonstrated efficacy as a broad‐spectrum antiviral strategy, but limited consideration has been given to the effects on host glycoprotein production and consequent disruption of signalling cascades. This work demonstrates that iminosugars inhibit dengue virus, bacterial lipopolysaccharide and fungal antigen‐stimulated cytokine responses in human macrophages. In spite of decreased inflammatory mediator production, viral replication is suppressed in the presence of iminosugar. Transcriptome analysis reveals the key interaction of pathogen‐induced endoplasmic reticulum stress, the resulting unfolded protein response and inflammation. Our work shows that iminosugars modulate these interactions. Based on these findings, we propose a new therapeutic role for iminosugars as treatment for sepsis‐related inflammatory disorders associated with excess cytokine secretion.

## Graphical abstract

Iminosugars are a class of small molecule glycomimetics with distinct inhibition profiles for glycan processing enzymes based on stereochemistry. We report a distinct activity of iminosugars whereby modulation of the unfolded protein response occurs as a result of inhibition of ER‐resident glycoprotein processing enzymes. This results in decreased inflammation arising from diverse pathogens including replicating virus, bacterial endotoxin and fungal antigen.

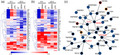

**Abbreviations**
ANOVA: analysis of variance
ANP32A: acidic nuclear phosphoprotein 32 family member A
ATF6: activating transcription factor 6
CRELD2: cysteine rich with EGF‐like domains 2
DENV: dengue virus
EIF2AK3/PERK: eukaryotic translation initiation factor 2 activating kinase 3/PKR‐like ER kinase
ELISA: enzyme‐linked immunosorbance assay
ER: endoplasmic reticulum
FDR: false discovery rate
G‐CSF: granulocyte colony‐stimulating factor
gMFI: geometric mean fluorescence intensity
HERPUD1: Homocysteine‐inducible, endoplasmic reticulum stress‐inducible ubiquitin‐like domain member 1
HMGB1: high mobility group box 1
IC50: dose of drug required for 50 per cent reduction in infectious virus titre
IC90: dose of drug required for 90 per cent reduction in infectious virus titre
IFN‐γ: interferon‐γ
IL‐10: interleukin 10
IL‐17A: interleukin 17A
IL‐4: interleukin 4
IL‐6: interleukin 6
IL‐8: interleukin 8
IP‐10: C‐X‐C motif chemokine ligand 10
IRE1/ERN: inositol requiring enzyme 1/endoplasmic reticulum to nucleus signalling 1
LPS: lipopolysaccharide
M1: classical macrophage activation
M2: alternative macrophage activation
MCP‐1: C‐C motif chemokine ligand 2
MIF: macrophage migration inhibitory factor
MIP‐1β: C‐C motif chemokine ligand 4
MOI: multiplicity of infection
MON‐DNJ: N‐(9‐methoxynonyl)‐1‐deoxynojirimycin
NB‐DGJ: N‐butyl‐deoxygalactonojirimycin
NB‐DNJ: N‐butyl‐deoxynojirimycin
NUCB2: nucleobindin 2
p.i.: post‐infection
p38a MAPK: p38a mitogen‐activated protein kinase
PBMC: peripheral blood mononuclear cell
PCA: principal component analysis
PGM3: phosphoglucomutase 3
PPP2CB: protein phosphatase 2 catalytic subunit beta
qRT‐PCR: quantitative reverse transcriptase polymerase chain reaction
RANTES: C‐C motif chemokine ligand 5
ROS: reactive oxygen species
RPLP1: ribosomal protein lateral stalk subunit p1
RPLP2: ribosomal protein lateral stalk subunit p2
SUMO1: small ubiquitin‐like modifier 1
TLR1: Toll‐like receptor 1
TLR‐4: Toll‐like receptor 4
TNFRSF9: TNF receptor superfamily member 9
TNF‐α: tumour necrosis factor‐α
TRAF1: TNF receptor‐associated factor 1
UGDH: UDP‐glucose 6‐dehydrogenase
UI: uninfected
UPR: unfolded protein response

## INTRODUCTION

Recent changes to the consensus definitions of sepsis and septic shock highlight a shift in clinical risk stratification to identify patients at greater risk of mortality through a focus on dysregulation of the immune response to invading pathogens [1]. The complexity of dynamic immune responses complicates identification of individual proteins or signalling networks that are responsible for sepsis. Nevertheless, there is evidence for dysregulation of several systems contributing to septic pathophysiology including excessive inflammation, coagulopathy, endothelial dysfunction, immune suppression, epigenetic alteration and metabolic dysregulation [2, 3, 4]. The most thoroughly explored avenue for development of sepsis is excessive inflammation through a process often described as a ‘cytokine storm’. Unfortunately, approaches aimed at inhibiting expression or signalling by single molecules expressed early in the onset of inflammation (e.g. TNF‐α) have failed to improve mortality in sepsis in individual trials although a meta‐analysis suggests potential benefit [5]. Systemic anti‐inflammatory therapy (e.g. with glucocorticosteroids) has demonstrated similar mixed efficacy in clinical trials [6, 7]. Novel strategies are therefore desirable to identify possible therapeutic strategies.

In this study, we investigated the use of the iminosugar *N*‐(9‐methoxynonyl)‐1‐deoxynojirimycin (M*O*N‐DNJ) as a potential therapy for attenuation of the excessive response to infection. Iminosugars are promising candidates for treatment of dengue virus (DENV) infection with antiviral efficacy demonstrated in cell culture and animal models [8, 9, 10]. These glycan mimics inhibit host glycoprotein processing enzymes necessary for correct folding of viral glycoproteins and therefore infectious virus production, and this activity is responsible for reduction of infectious virus burden [11, 12]. Prior work *in vivo* has demonstrated M*O*N‐DNJ‐mediated reduction of cytokine expression in DENV‐infected mice [9, 13], but the reduction in infectious virus obfuscates the mechanism of cytokine reduction.

Because of the host‐directed nature of iminosugars, we hypothesized that inflammatory signatures generated in response to DENV infection might be altered in the presence of iminosugar independent of changes in infectious virus production. We have recently demonstrated iminosugar‐mediated interference of IFN‐γ and TNF‐α receptor signalling as well as mannose receptor binding in the context of infection [14]. Bicyclic sphingolipid mimic iminosugars have demonstrated the capacity to reduce inflammation in chronic disease conditions (e.g. diabetic retinopathy) via direct binding of p38a MAPK [15, 16]. We therefore sought to investigate the potential for iminosugar to alter the network‐level cellular response to diverse pathogens.

The unfolded protein response (UPR), is one such network‐level response to cellular stress, whereby excess unfolded proteins are detected in the endoplasmic reticulum (ER) and three interdependent pathways are activated to manage the ER stress response. The three arms of the UPR: IRE1/ERN, ATF6 and EIF2AK3/PERK pathways, serve to increase folding of nascent ER proteins while providing crucial feedback signals to inflammation, autophagy, apoptosis and reactive oxygen species pathways among others. Our group and others have reviewed these complex dynamics in cellular stress elsewhere [17, 18]. Given the inhibition of glycoprotein folding caused by iminosugars [11, 12], we hypothesized that iminosugars would induce UPR. We reasoned that the significant interdependence of the UPR with innate inflammation and generation of reactive oxygen species (ROS) could provide a mechanism whereby iminosugar‐induced blockade of glycoprotein processing would have pathogen‐independent effects on inflammation. We further hypothesized that changes to inflammation would extend to diverse pathogens including bacteria and fungi commonly implicated in sepsis. To evaluate these hypotheses, we compared M*O*N‐DNJ‐mediated modulation of network‐level responses to DENV infection with M*O*N‐DNJ‐mediated changes arising from activation of the pattern recognition receptor TLR4 signalling by lipopolysaccharide (LPS) in macrophages.

## RESULTS

### Iminosugars attenuate inflammatory cytokine production in macrophages

**FIGURE 1:**
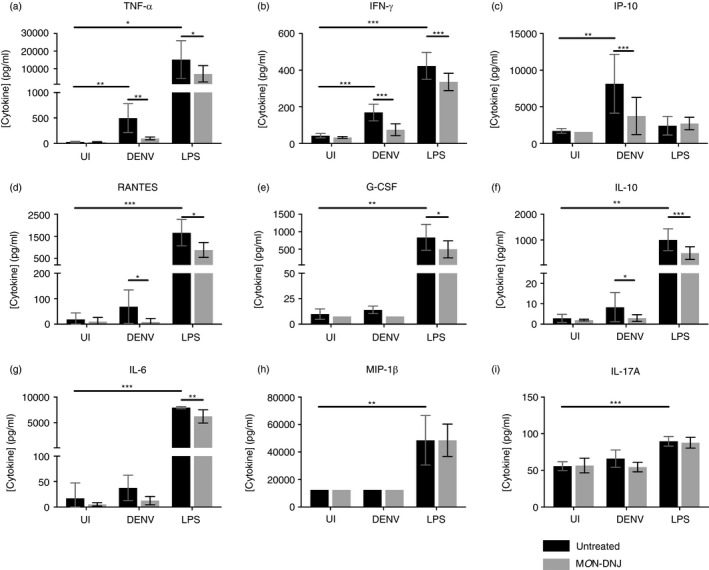
M*O*N‐DNJ reduces DENV‐ and LPS‐induced inflammation in macrophages. (a–i) Nine of twelve cytokines assayed on a Luminex bead‐based platform at 24 h p.i. demonstrate differential expression in macrophages treated with media (UI), DENV 2 16681 at moi =1 (DENV) or LPS from *S*. *enterica* at 100 ng/ml (LPS). Equivalent data were collected and analysed at 72 and 120 h p.i. (Figure S2). M*O*N‐DNJ samples (grey bars) were compared to untreated samples (black bars) by one‐way, repeated‐measures ANOVA or equivalent ANOVA of ranks for non‐normally distributed data with post hoc pairwise comparison using the Holm–Šídák method (parametric) or Tukey test (non‐parametric). Induction of cytokine by each mode of infection was tested independently by ANOVA with correction for multiple comparisons across time points using Holm–Šídák method. Biological replicates (*n* = 5) were assayed in technical singlicate. Discontinuous axes are used where necessary as a consequence of >10‐fold difference in level of cytokine induced by DENV and LPS. Samples were normalized to untreated controls for individual donor and stimulus and evaluated by parametric *t*‐testing with Holm–Šídák correction for multiple testing. *p < 0·05, **p < 0·01, ***p < 0·001

**FIGURE 2:**
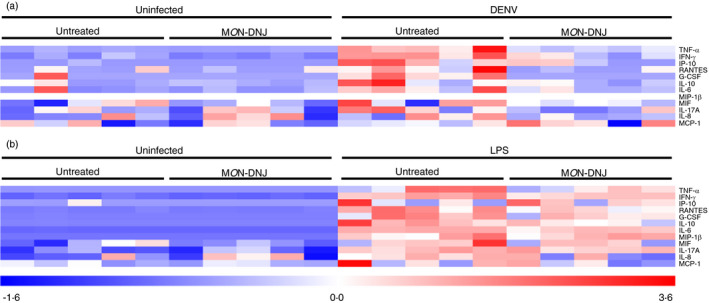
M*O*N‐DNJ dampens cytokines with donor‐to‐donor variability. (a) Protein‐level data displayed in (Figure 1) were converted to a heatmap with normalization ([value‐mean]/standard deviation) of each cytokine using TMEV for DENV infection at 24 h p.i. Each box represents the normalized level of cytokine for a single donor. (b) Protein‐level cytokine data were normalized for LPS in an identical fashion as for DENV. Darker red represents greater relative cytokine concentration, and darker blue represents lower relative cytokine concentration

**FIGURE 3:**
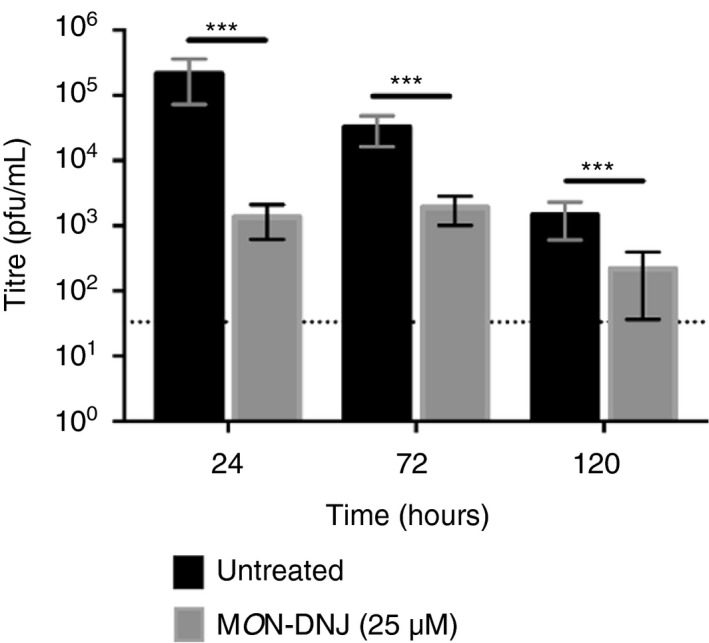
Sustained antiviral efficacy of M*O*N‐DNJ. Cell culture supernatants were collected at the time points indicated (*n* = 4 biological replicates), and infectious DENV titre was detected by LLC‐MK2 plaque assay (technical triplicate) as previously described [45]. Untreated samples (black bars) were compared to M*O*N‐DNJ‐treated samples (grey bars) using repeated‐measures ANOVA with correction for pairwise comparison using the Holm–Šídák method. Error bars represent standard deviation. *p < 0·05, **p < 0·01, ***p < 0·001

Macrophages play a central role in directing the immune response to sepsis [19] and are among the determinant cells in the outcome of DENV infection in humans, as target cells of DENV and orchestrators of the innate immune response required for viral control [20]. To investigate the effects of iminosugars on macrophages, we used a model that increases the susceptibility of macrophages to infection by DENV [21, 22, 23, 24] (Figure S1) to evaluate cytokine production in response to pathogen (Figure 1). Whereas LPS stimulation induced 8 of 12 cytokines tested by 24 h post‐infection (p.i.), cytokine induction by DENV was limited to TNF‐α, IFN‐γ and IP‐10 at 24 h suggesting a specific role of these cytokines in initiating the macrophage response to DENV. IL‐8, MCP‐1 and MIF were not induced by either DENV or LPS at 24 h (data not shown). By 72 h p.i., 9 of 12 cytokines tested were induced by DENV infection and this was sustained at 120 h (Figure S2). In the presence of 25 µM M*O*N‐DNJ, a statistically significant reduction in cytokine level was detected for all induced cytokines at 24 h p.i. irrespective of viral or bacterial stimulus with the exception of LPS‐induced MIP‐1β (Figure 1h) and IL‐17A (Figure 1i). Lack of M*O*N‐DNJ modulation of MIP‐1β and IL‐17A in the presence of LPS suggests that cytokine effects are specific to a limited subset of cytokines and not reflective of global reduction of secreted proteins. Results of statistical significance testing for all cytokines at all time points are shown in Table S1. A heat map of normalized cytokine protein expression demonstrates the donor variability of cytokine expression in DENV infection (Figure 2b) and LPS treatment (Figure 2B) and further reveals that the statistically significant reduction in cytokine levels with M*O*N‐DNJ does not lead to complete reduction of cytokine to uninfected, untreated levels (i.e. columns remain pink rather than blue). Steroid treatment in the same model results in reduced cytokine production and an antiviral effect in the first 24 h; however, in the presence of steroid infectious virus production rebounds to untreated levels by 72 h p.i. [25]. In contrast, antiviral efficacy is maintained with M*O*N‐DNJ treatment up to at least 120 h p.i. (Figure 3) suggesting that the reduced inflammation does not potentiate virus replication.

**FIGURE 4:**
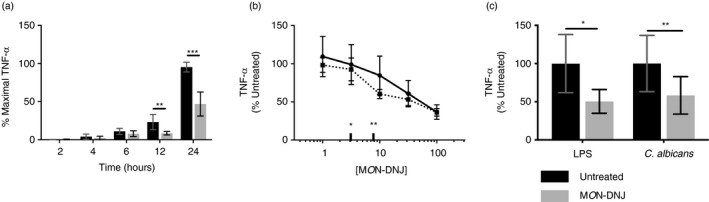
M*O*N‐DNJ reduces TNF‐α produced by macrophages in response to viral, bacterial and fungal antigens. (a) 24‐h time course of TNF‐α in response to DENV infection was undertaken (*n* = 4 biological replicates) to assess the time at which M*O*N‐DNJ (grey bars) begins to limit cytokine production. Total cytokine was measured by ELISA (technical duplicate) and the maximal TNF‐α level observed for each donor was set to 100 per cent. Repeated‐measures ANOVA on the arcsine transform of normalized data was used to assess significance with Holm–Šídák method of multiple comparisons. (b) The dose–response relationship of M*O*N‐DNJ and total TNF‐α production (solid line) in DENV‐infected macrophages at 24 h p.i. was assessed by ELISA in technical duplicate (*n* = 3 biological replicates). Functional TNF‐α (dashed line) was assayed in HEK‐blue cells in technical and biological triplicate. The IC_50_ (*) and IC_90_ (**) of M*O*N‐DNJ for reduction of infectious DENV in this model [8] are indicated for comparison to reduction of cytokine. (c) Functional TNF‐α secretion in response to LPS and heat‐killed *C*. *albicans* at 24 h p.i. was assayed in HEK‐blue cells for 3 biological replicates each assayed in technical triplicate as in (b). Samples were normalized to untreated controls for individual donor and stimulus and evaluated by parametric *t*‐testing with Holm–Šídák correction for multiple testing. *p < 0·05, ** *p* < 0·01, ***p < 0·001

Whereas cytokines produced in LPS‐treated cells are not dependent on pathogen replication, cytokines produced in DENV‐infected macrophages are elicited in the context of active viral replication. To assess whether reduced cytokine in DENV infection is solely dependent upon reduced virus production, we performed time course (Figure 4a) and M*O*N‐DNJ titration (Figure 4b) experiments. The flavivirus infectious cycle in cell culture is virus and cell‐type dependent but generally results in release of progeny virus around 12 h p.i. [26, 27, 28], and in our model, statistically significant reduction of cytokine with M*O*N‐DNJ is concurrent with this time although the difference at 6 h p.i. approaches significance (*p* = 0·0517). The dose–response relationship of M*O*N‐DNJ to total and functional TNF‐α production appears to extend beyond the antiviral IC_90_ (dose of drug required for 90 per cent reduction in infectious virus titre, Figure 4b**). Furthermore, functional levels of TNF‐α are reduced with M*O*N‐DNJ treatment of cells stimulated with non‐replicating pathogen‐associated ligands including LPS and heat‐killed *Candida albicans* (Figure 4c). Taken together, these results suggest that iminosugar‐mediated reduction of TNF‐α is not exclusively dependent on inhibition of viral replication.

### Iminosugars modulate the macrophage transcriptome

**FIGURE 5:**
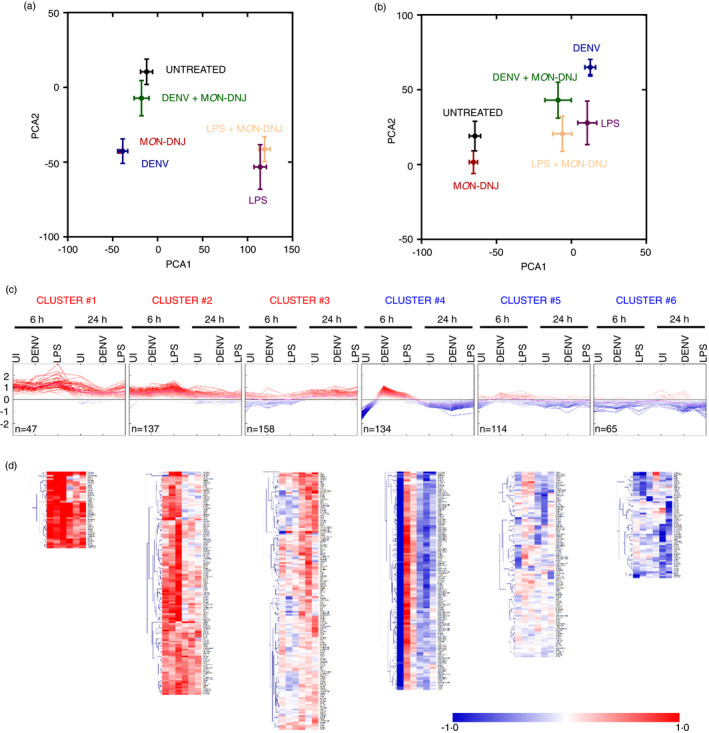
Characterization of M*O*N‐DNJ transcriptomic effects. Principal component analysis (PCA) of all expressed transcripts at 6 h p.i. (a) and 24 h p.i. (b) was performed in ClustVis. Unit variance scaling is applied to rows, and singular value decomposition is used to calculate principal components. X‐ and Y‐axes show principal component 1 (PCA1) and principal component 2 (PCA2) that explain 54 per cent and 36 per cent of the total variance, respectively. Error bars represent standard error. (c) The 655 transcripts (324 genes, Table S2) with the greatest differential expression in our dataset (as described in Figure S1b) were subjected to K means clustering into 6 groups based on Euclidean distance. Expression patterns are noted with fold change (log_2_) for M*O*N‐DNJ treatment relative to each untreated infection condition at each time point displayed on the x‐axis as an averaged value of *n* = 5 biological replicates. Individual transcripts are represented by a single line, and colour further represents magnitude of fold change such that darkest red is ≥1 log_2_ induction with M*O*N‐DNJ relative to untreated and darkest blue is ≥1 log_2_ down‐regulation with M*O*N‐DNJ relative to untreated. (d) Unsupervised hierarchical clustering (Euclidean distance, complete linkage) was performed on the clusters. Average fold change (log_2_) in gene expression of *n* = 5 biological replicates with iminosugar treatment is represented by a single coloured box

**FIGURE 6:**
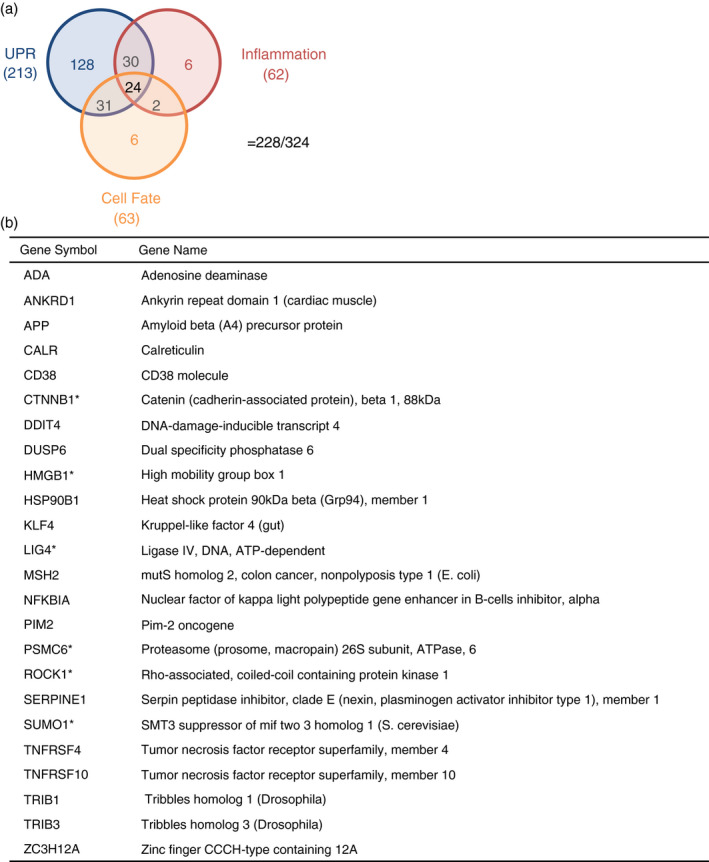
Differentially expressed genes overlap in molecular function between UPR, inflammation and signalling of cell fate. (a) The list of all biological processes (Table S3D) over‐represented by M*O*N‐DNJ‐modulated transcripts (Table S2) was curated to identify processes associated with the UPR (Table S4A), inflammation (Table S4B) and signalling of cell fate (Table S4C), and the overlap between the genes involved with these processes was identified as represented by the Venn diagram. Of the 324 differentially expressed genes identified, 228 are involved in these three networks. The total number of genes for each process is listed under the process label, and the number of genes in each intersection set is represented by graphical location. (b) The 24 genes that have a curated involvement in all three pathways are listed with those demonstrating the highlighted expression pattern in Figure 7 represented by bold font and an asterisk

To identify pathways involved in the disruption of inflammation by M*O*N‐DNJ, a time course of macrophage transcriptomic responses to DENV, LPS and M*O*N‐DNJ was investigated. An early time point of 6 h p.i. was chosen to evaluate changes to transcriptional patterns independent of the effects of infectious virus release, and a further point at 24 h p.i. was chosen to capture changes occurring in the presence of replicated virus and to allow comparison of the dynamic changes likely to be occurring. A total of 21,705 transcripts were expressed above background in at least one sample and these probes were subjected to principal component analysis (PCA) to identify changes to gene signatures (Figure 5a,b). Principal components 1 and 2 account for 54 per cent and 36 per cent of the variance of the entire dataset, respectively. In general, there were relatively modest changes to the transcriptome with DENV and iminosugar treatment in comparison with LPS. Comparing uninfected to DENV‐infected and LPS‐treated samples in the absence of drug treatment demonstrates the dynamic macrophage transcriptomic responses to stimuli. As anticipated, switching from alternate (M2) activation stimulation with IL‐4 to classical (M1) activation with LPS induces a profound and early shift in transcript levels with 9,188 and 6,142 transcripts modulated at 6 and 24 h, respectively. In comparison, DENV infection induces statistically significant change in 914 and 6,517 transcripts at 6 and 24 h, respectively. We focus on conserved changes to the macrophage transcriptome induced by treatment with M*O*N‐DNJ (Figure S1b) with 655 differentially expressed probes (mapping to 324 unique genes, Table S2) of particular interest. These 655 probes were analysed by K means clustering (Figure 5c) to identify patterns of response to M*O*N‐DNJ. Two major response patterns, induction (cluster 1–3, Figure 5c) and down‐regulation (cluster 4–6, Figure 5c), were identified. Difference in timing of response to M*O*N‐DNJ appears to be the principal factor that differentiates sub‐clusters (e.g. sustained induction in cluster 1 vs. early induction only in cluster 2 vs. late induction only in cluster 3). Unsupervised hierarchical clustering (Euclidean distance with complete linkage) was performed (Figure 5d), and transcripts associated with each cluster were analysed in STRING‐DB to assess for enrichment of genes associated with biological processes. Of note, cluster 1 and cluster 2 are heavily associated with ER stress and the UPR (Table S3A,B). Cluster 6 is the only down‐regulated gene cluster with STRING‐DB‐identified enrichment of biological processes, and those processes identified are almost exclusively associated with inflammation (Table S3C). Additional biological processes associated with the 655 differentially expressed probes were identified in STRING‐DB using the entire unclustered dataset (Table S3D). These networks can generally be classified into three categories: ER UPR (Table S4A), inflammation (Table S4B) and cell fate (e.g. autophagy vs. apoptosis signalling, Table S4C). These three networks account for 228 of the 324 differentially expressed genes, and 24 genes are involved in all three processes (Figure 6).

**FIGURE 7:**
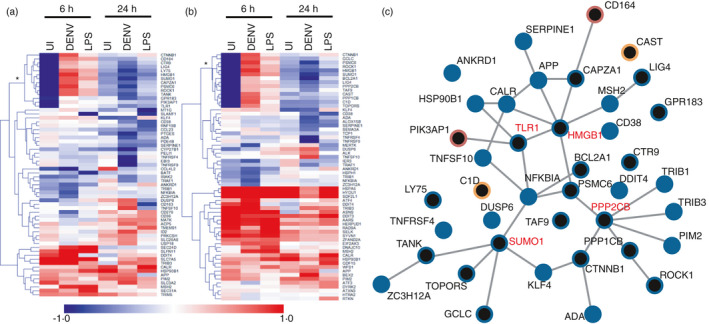
M*O*N‐DNJ modifies inflammatory and cell fate transcriptional signatures. (a) Heatmap of M*O*N‐DNJ modulated transcripts related to inflammation. Genes for clustering were identified from the intersection of those differentially expressed with M*O*N‐DNJ treatment as presented in Table S4B. Average fold change (log_2_) in gene expression of *n* = 5 biological replicates with iminosugar treatment is represented by a single coloured box. HCL using Euclidean distance identifies a subset of genes (*) with early inhibition in uninfected macrophages that extends to all infection conditions by 24 h. (b) Heatmap of M*O*N‐DNJ modulated transcripts related to cell fate. Genes for clustering were identified from the intersection of those differentially expressed with M*O*N‐DNJ treatment as presented in Table S4C. Average fold change (log_2_) in gene expression of *n* = 5 biological replicates with iminosugar treatment is represented by a single coloured box. HCL using Euclidean distance identifies a subset of genes (*) with early inhibition in uninfected macrophages that extends to all infection conditions by 24 h. Six of the fourteen genes identified overlap with the labelled group in (a). (c) The union set of the highlighted genes from (a) and (b) and Figure 6 was used to construct an interaction network in STRING‐DB. Nodes with involvement in at least two biological processes are represented by blue circles while those with involvement in a single biological process are red (inflammation) or orange (cell fate). Black central colouring indicates the gene is a member of the union set identified by the asterisk in (a) and/or (b). Published, curated interactions are represented by solid lines between nodes for all interaction scores >0·4. Nodes conforming to the identified regulatory pattern and having at least 4 connections are highlighted in red text

Extensive overlap between the UPR and inflammation has been observed [18, 29], and the particular role of the UPR in DENV infection has also recently gained interest [17]. As M*O*N‐DNJ treatment reduces cytokine production and induces the UPR, we were interested to identify links between the systems that are altered with M*O*N‐DNJ treatment. To do so, we first identified genes that are associated with the immune response that are differentially expressed with M*O*N‐DNJ treatment as described in the Methods and generated a heat map of mean fold change with iminosugar treatment for unsupervised hierarchical clustering (Figure 7a). Among these 62 genes, 24 overlap with the previously described cluster 6 associated with inflammation. An additional node of 14 genes (*) was of particular interest. These genes exhibit strong down‐regulation with M*O*N‐DNJ in uninfected macrophages 6 h p.i., and by 24 h p.i., the transcript level is reduced in the presence of M*O*N‐DNJ irrespective of infection conditions. This pattern was identified by K means clustering (Figure 5c,d, cluster 4) to match 134 total transcripts (mapping to 67 genes, Table S5). Given the abundance of transcripts with this interesting response pattern, identical methods were applied to the differentially expressed genes associated with signalling of cell fate, and 14 transcripts (6 of which were included in the inflammation list) with a similar response were identified (Figure 7b). We therefore generated a network of known interactions for the union set of these genes in addition to the 24 differentially expressed genes with published involvement in UPR, inflammation and cell fate determination (Figure 6) using STRING‐DB (Figure 7c). The full gene name is provided in Table S6 for all protein members of the network in Figure 7c. Several nodes within this network demonstrate a high level of interaction including HMGB1, PPP2CB, TLR1 and SUMO1—all of which are connected to at least 4 other network members and conform to the previously described transcriptional response pattern (highlighted in red text in Figure 7c). Because of the biologically interesting intersection of pathways at HMGB1, we investigated total protein secretion in a small subset of donors (*n* = 2); however, we were not able to identify any consistent pattern of modulation with M*O*N‐DNJ (Figure S3).

**FIGURE 8:**
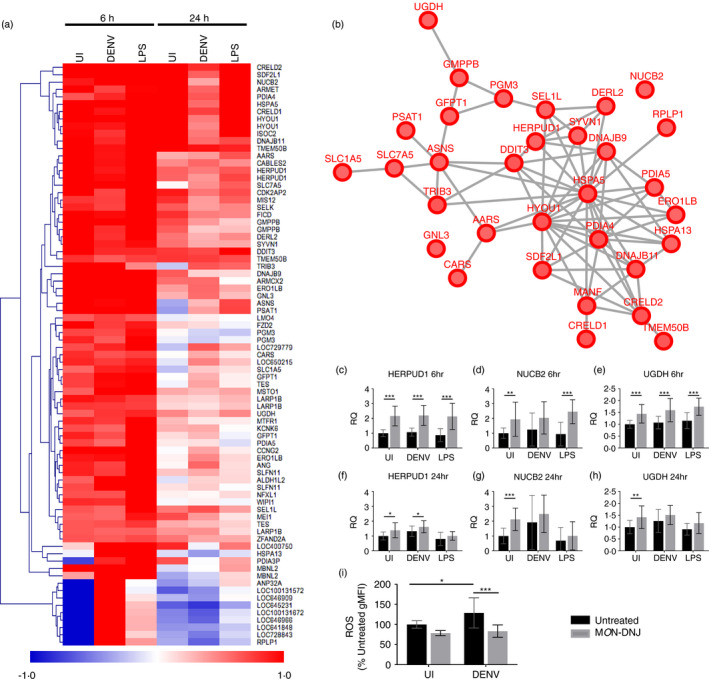
The unfolded protein response is induced by M*O*N‐DNJ. (a) Heatmap of M*O*N‐DNJ modulated transcripts. Genes for clustering were identified as described in Figure S1b. Average fold change (log_2_) in gene expression of *n* = 5 biological replicates of M*O*N‐DNJ treatment in comparison with infection and time‐matched controls is represented by a single coloured box. (b) STRING‐DB network of the genes listed in (a). Interactions curated in STRING‐DB are represented by lines between nodes for all interaction scores >0·4. (c–h) Selected transcripts were assayed by qRT‐PCR to confirm differential regulation observed by microarray. Macrophages from *n* = 4 donors were infected and treated with control (black bars) or 25 μM M*O*N‐DNJ (grey bars) in duplicate and RNA was collected and assayed by qRT‐PCR in technical duplicate. Holm–Šídák correction for multiple comparisons was performed to identify statistically significant differences. All error bars represent standard deviation. RQ = relative quantity based on normalization to unmodulated transcript RPLP2. (a) Reactive oxygen species (ROS) generated in macrophages at 18 h p.i. was assessed using a flow cytometry‐based detection assay. Biological replicates (*n* = 3) were measure in technical triplicate, and all samples generated for a given donor were normalized to the uninfected, untreated control (black bar, UI). Two‐way ANOVA with Holm–Šídák correction for multiple comparisons was performed to identify statistically significant differences. *p < 0·05, **p < 0·01, ***p < 0·001

Our initial clustering analysis (Figure 5) suggests that the strongest signature associated with M*O*N‐DNJ treatment is for induction of the UPR, and this network was further investigated. Among transcripts with the greatest differential expression, 79 transcripts mapping to 57 genes had at least twofold expression change in two of the three infection conditions (uninfected, DENV and LPS) with drug treatment at 6 h p.i. or at least 1·5‐fold expression change in all three conditions. A heatmap of the fold change of these genes (Figure 8a) was generated with 33 genes mapping to a single network (related to the UPR, Figure 8b). Notably, almost all genes identified in this manner have elevated expression with M*O*N‐DNJ across infection groups at 6 h with the exception of RPLP1 and ANP32A. Unsupervised hierarchical clustering demonstrates a robust rise in transcript at 6 h p.i. with a variable return towards baseline by 24 h. Both the persistently induced genes (e.g. CRELD2) and the transiently induced genes (e.g. PGM3) demonstrate involvement in the UPR including all three major arms (IRE1/ERN, ATF6 and EIF2AK3/PERK) [18]. Quantitative reverse transcriptase PCR (qRT‐PCR) was used to validate the strong pattern of early UPR gene induction with M*O*N‐DNJ for representative members of the M*O*N‐DNJ‐modulated network generated in Figure 8b. These genes (HERPUD1: homocysteine‐inducible, endoplasmic reticulum stress‐inducible, ubiquitin‐like domain member 1, NUCB2: nucleobindin 2 and UGDH: UDP‐glucose 6‐dehydrogenase) are all components of the UPR with strong induction noted in our transcriptomic data and qRT‐PCR confirms induction with M*O*N‐DNJ (grey bars) with return towards baseline levels at 24 h p.i. (Figure 8c–h).

### Iminosugars reduce generation of reactive oxygen species

Association between the UPR, TNF‐α and generation of reactive oxygen species (ROS) in DENV infection [30] and the role of ROS (and reactive nitrogen species) in severe DENV disease [31] led us to investigate whether M*O*N‐DNJ could alter the generation of ROS. As anticipated, DENV infection of macrophages induces ROS (Figure 8i). With M*O*N‐DNJ treatment in DENV‐infected macrophages, ROS are restored to baseline suggesting that M*O*N‐DNJ is able to control the generation of potentially damaging free radical species in addition to inflammatory cytokines. Thus, M*O*N‐DNJ treatment appears to reduce oxidative stress and inflammation while modulating the unfolded protein response and maintaining effective antiviral activity.

## DISCUSSION

These studies provide the first comprehensive characterization of the effects of the iminosugar M*O*N‐DNJ on host processes. In so doing, we have identified a mechanism of action that expands the therapeutic potential specifically of M*O*N‐DNJ and more generally of ER α‐glucosidase inhibiting iminosugars. In addition to reducing infectious virus production, M*O*N‐DNJ is able to reduce pro‐inflammatory cytokine production induced by actively replicating viral pathogen in addition to that caused by the TLR4 ligand LPS and *C. albicans* fungal antigen. The reduced cytokine production is co‐ordinated with a reduction in ROS further suggesting reduced oxidative stress as a consequence of iminosugar treatment. The UPR is robustly induced by M*O*N‐DNJ treatment, a result that is in keeping with the inhibition of ER‐resident α‐glucosidases necessary for glycoprotein processing [11, 12]. Although there appears to be a return to baseline for many of the induced UPR‐associated genes by 24 h p.i., the functional consequences of iminosugar treatment (i.e. cytokine reduction and antiviral efficacy) appear to be longer in duration. Such observations are consistent with our recent finding that a single large dose of iminosugar late in the course of DENV or influenza virus infection confers a survival benefit in murine models [32]. Indeed, the experiments presented herein demonstrate that the limited set of genes related to inflammation that are modulated by iminosugars are most consistently suppressed at 24 h p.i. Taken in concert with our prior murine data, this suggests that a single‐dose, high concentration bolus of iminosugar late in the course of diseases with acute dysregulation of inflammation (such as viral, bacterial and fungal sepsis) may be an effective means of controlling the disease process.

Although induction of the UPR is generally associated with increased inflammation in macrophages [18, 33], there are distinct roles for each of the three arms of the network, and pathogens are known to actively antagonize particular elements of the response to favour their own replication and avoid the anti‐pathogenic elements of the UPR [34]. By overriding pathogen‐mediated regulatory responses through wholesale manipulation of the UPR by addition of iminosugar, we hypothesize that the balance of UPR and inflammatory responses is restored in favour of anti‐pathogenic pathways. The finding that a small number of genes associated with both the UPR and inflammation are down‐regulated at an early time point following iminosugar treatment is suggestive of a mechanism whereby inhibiting the cell's N‐linked glycoprotein processing leads to an altered transcriptional profile that favours a limited but productive inflammatory phenotype. It is tempting to speculate that one of these genes provides a singular mechanistic link. Indeed, we considered that HMGB1 could be solely responsible for the changes observed, but we did not detect a difference in level of secreted protein with iminosugar treatment (Figure S3). However, HMGB1 signalling is complex [35, 36, 37, 38, 39] and dependent upon a number of factors including oxidation state and subcellular localization, and these details may underlie a physiologic explanation for our observations.

While there is much support in the literature for a molecule such as HMGB1 playing a key role in mediating M*O*N‐DNJ‐induced dampening of inflammation [40], particularly in DENV disease [41, 42, 43, 44], it is also probable that the concerted manipulation of several/all of these genes and their downstream signalling is essential for successful clearance of pathogen in the context of a controlled inflammatory milieu. The data presented herein demonstrate that iminosugars can control inflammation in viral, bacterial and fungal sepsis. These results expand the therapeutic potential of iminosugars to include control of inflammation in diverse pathologies irrespective of their ability to limit pathogen replication.

## METHODS

### Virus

DENV2 strain 16681 (a gift from G. Screaton, Oxford, UK) was propagated in mosquito C6/36 cells (a gift from Armed Forces Research Institute of Medical Sciences, Thailand), collected from supernatant and concentrated by precipitation with 10% (w/v) poly(ethylene glycol) M_r_ 6,000 (Sigma), 0·6% sodium chloride (Sigma) overnight at 4℃. Following precipitation, virus was centrifuged at 2830 *g* for 45 min at 4℃, resuspended in Leibovitz's L15 media +10% HI FBS and stored at −80℃ until use. Virus titres were obtained by plaque assay on LLC‐MK2 monkey kidney cells (a gift from Armed Forces Research Institute of Medical Sciences, Thailand), as described previously [45].

### Iminosugars

The iminosugar compounds *N*B‐DNJ (solubilized in PBS, Oxford GlycoSciences Ltd.), M*O*N‐DNJ (solubilized in acidified water, United Therapeutics) and *N*B‐DGJ (solubilized in 83% DMSO, Toronto Research Chemicals) were verified to contain less than 0·05 endotoxin units per mL.

### Isolation of monocytes and macrophage model

Human PBMCs (peripheral blood mononuclear cells) were isolated from buffy coats (NHS Blood and Transport) by centrifugation over a Ficoll‐Paque^TM^ PLUS (Amersham) gradient and monocytes isolated by adherence as previously reported. Autologous plasma was collected, heat‐inactivated (56℃, 30 min) and used to supplement (1%) X‐VIVO10 (Lonza) medium to produce complete growth medium. Cells were differentiated for 3 days (37℃, 5% CO_2_) in complete growth medium +25 ng/ml recombinant human IL‐4 (rhIL‐4, Peprotech) to generate alternatively activated macrophages [14, 21]. The use of human blood was approved by the NHS National Research Ethics Service (09/H0606/3).

### Macrophage stimulation, infection and drug treatment

Macrophages were stimulated with LPS (200 ng/ml from *Salmonella enterica*, Sigma), heat‐killed *Candida albicans* (2 × 10^6^ c/ml, Invivogen), unstimulated (media‐only) or infected with DENV2 16681 diluted to a multiplicity of infection (MOI) of 1, in X‐VIVO10 without supplements for 90 min (20℃, with rocking). Subsequently, virus or media was removed and replaced with fresh complete growth medium without IL‐4, but containing M*O*N‐DNJ (25 µM, unless otherwise indicated) or media‐only control. For LPS and *C*. *albicans*, stimulus was not removed, and M*O*N‐DNJ (25 μM final concentration) or control medium was added such that LPS stimulation continued at 100 ng/ml and *C*. *albicans* stimulation at 1 × 10^6^ c/ml. Cells were incubated for indicated times (37℃, 5% CO_2_) before supernatant harvesting and centrifugation for 5 min (room temperature, 400 *g*) to pellet any cells/debris. Aliquots were stored at −80℃ until analysis.

### Luminex detection of cytokines

Cytokines/chemokines (IL‐6, IL‐8, IL‐10, IL‐17A, G‐CSF, IFN‐γ, IP‐10, MCP‐1, MIF, MIP‐1β, RANTES and TNF‐α) were detected by a multiplex fluorescent bead‐based assay (Bio‐Rad) in supernatants following treatments described above. Collected supernatants were centrifuged (5 min, 900 *g*) to remove cellular debris, aliquoted and stored at −80℃ until analysis. Samples were handled in a 96‐well format using a filter‐bottom plate (Bio‐Rad) to allow washing of magnetic beads as per the manufacturer's instructions and analysed on a Luminex 200 (Luminex) fluorescence detector. Concentrations of cytokines were determined based on 5‐point linear regression curves in comparison with standards. The iminosugars *N*B‐DNJ, *N*B‐DGJ and M*O*N‐DNJ were used to treat four donors, and statistical evaluation was performed on all cytokines with correction for multiple comparisons as reported in Table S1. Statistical analyses were performed using SigmaPlot 12 (Systat Software) using either parametric *t*‐tests or ANOVA or, in the case, where data compared did not meet necessary assumptions of normality by Shapiro‐Wilk test or equality of variance by *F*‐test, non‐parametric *t*‐tests or ANOVA. Post hoc testing was conducted by Holm–Šídák method for parametric methods or by Dunnett's test for non‐parametric methods. Data in the main text are limited to M*O*N‐DNJ for clarity; however, all significance is corrected for multiple comparisons based on all three iminosugars tested.

### Analysis of cytokines by ELISA

Supernatant TNF‐α concentration was determined by enzyme‐linked immunosorbent assay (ELISA), based on manufacturer supplied TNF‐α standard curve (Invitrogen, KHC3011). DENV‐infected samples were diluted 1:2, and LPS‐treated samples were diluted 1:10 in X‐VIVO10 to ensure cytokine levels were within the range of the standard curve. IP‐10 and MIP‐1β cytokine levels were quantified by ELISA using Quantikine kits (R&D Systems) based on manufacturer supplied cytokine standard curve, with all supernatants diluted 1:100 in X‐VIVO10 (with centrifugation for 4 min at 2,000 *g*). ELISAs were conducted as per the manufacturer's instructions. Plates were read on a SpectraMax M5 microplate reader (Molecular Devices) to determine absorbance at 450 nm with subtractive correction of absorbance at 540 nm. Samples were assayed in technical singlicate with biological replicates averaged for statistical analyses. HMGB1 secretion was quantified by Shino‐Test ELISA (Tecan IBL, ST51011). All samples were diluted 1:2 in diluent buffer and assayed in technical duplicate according to the manufacturer's instructions for the high sensitivity standard curve. Absorbance at 450 nm was determined using a SpectraMax M5 microplate reader, and biological triplicates were averaged for statistical analysis. Analysis of significant differences was conducted as for Luminex assays above.

### Quantification of functional TNF‐α

HEK‐Blue™ TNF‐α cells (Invivogen) were cultured, and detection of biologically functional cytokine in stimulated macrophage supernatants was performed according to the manufacturer's instructions. Plates were incubated (37℃, 5% CO2) for 30 min to 1 h and secreted alkaline phosphatase levels quantified by measuring absorbance at 645 nm using a NOVOstar microplate reader (BMG Labtech). Cytokine concentration was determined relative to a standard curve of recombinant human TNF‐α (Peprotech) using GraphPad Prism version 7·01 (GraphPad Software, Inc).

### Transcriptomic sample generation and quality control

Macrophages were stimulated as previously described to generate time points post‐infection of 6 h and 24 h. Following appropriate incubation, cells were washed in 37℃ PBS (Sigma) then lysed with TRIzol (Life Technologies) (5 min, 20℃). Cellular debris was cleared by centrifugation (12,000 *g*, 1 min, room temperature), and RNA‐containing supernatants were mixed 1:1 with 100% EtOH (Fisher Scientific). Samples were applied to a Direct‐zol^TM^ RNA Mini‐prep column (Zymo Research) and washed in accordance with manufacturer's instructions. RNA was eluted by two sequential applications of 25 μl of RNase‐free water (50 μl total). Samples were stored at −80℃ and processed at Cambridge Genomic Services (CGS).

### Transcriptomic RNA quantification and normalization

RNA samples were amplified and biotinylated using the Illumina® TotalPrep^TM^ RNA Amplification Kit (Ambion), directly hybridized to a HumanHT‐12v4 BeadChip (Illumina) and scanned using an iScan system (Illumina). Illumina GenomeStudio analytical software was used to generate mappings and intensities and perform bead‐level processing (data available at: GSE128303). GenomeStudio generated data were imported into R Bioconductor v2·14 using the lumi package [46, 47, 48, 49]. Data were normalized via the neqc protocol [50] to account for variation in negative control probes. Inclusion criteria for further analysis required a detection value <0·01 for at least one sample for a given probe; this cut‐off allows for ‘on/off’ responses to various stimuli (e.g. DENV, LPS or M*O*N‐DNJ) and reduced the dataset to 21,705 expressed probes. GSE128303 contains the list of normalized expression values for all expressed probes.

### Statistical analyses of macrophage transcriptome in response to DENV, LPS and M*O*N‐DNJ

Differentially expressed genes were then identified for various treatments using an ANOVA approach implemented in lumi with thresholds requiring adjusted for multiple comparisons *p*‐value less than 0·01 and fold change greater than 20 per cent. Unsupervised hierarchical clustering was performed using Euclidean distance with complete linkage. Principal component analysis and hierarchical clustering of the entire transcript set were done using ClustVis [51]. Hierarchical clustering of transcript subsets identified in STRING‐DB was performed using TMeV [52, 53].

### STRING database identification of functional gene networks

Transcripts modulated in response to M*O*N‐DNJ treatment were identified in uninfected (UI), DENV‐infected (DENV) and LPS‐treated (LPS) macrophages at both 6 h and 24 h p.i. The top 100 differentially expressed transcripts identified by absolute value log_2_ fold change for each condition were merged in addition to all transcripts with at least a twofold change (absolute value log_2_ ≥ 1) to obtain a list of 655 differentially expressed probes mapping to 324 unique genes. This set was evaluated using STRING‐DB.org v10·5 [54, 55] for enrichment of biological processes (Tables [Link], [Link]).

To identify consistent changes with drug treatment, probes with at least a twofold change in 2 of 3 infection conditions at 6 h (*n* = 44) were combined with probes with at least a 1·5‐fold change in all 3 infection conditions at 6 h (*n* = 66) for a total of 79 probes mapping to 57 unique genes. At 24 h, an identical sorting process yields *n* = 12 probes with at least twofold change in 2 of 3 infection conditions and *n* = 8 probes with at least 1·5‐fold change in all 3 infection conditions (for 13 unique genes, Table S7). From the list of 57 genes identified at 6 h, STRING‐DB identified a single network of interactions principally associated with ER response to stress (FDR = 6·66 × 10^−16^), cellular response to topologically incorrect proteins (FDR = 6·66 × 10^−16^) and the ER UPR (FDR = 4·92 × 10^−15^). This network was used to generate the UPR‐associated heatmap, network image, and to evaluate enriched biological processes. Unsupervised hierarchical clustering using Euclidean distance, complete linkage was executed in TMeV based on mean fold change with M*O*N‐DNJ treatment compared to untreated controls.

Inflammatory transcriptional changes were identified from the set of 324 unique genes identified. STRING‐DB gene ontology enrichment identified 9 pathways associated with the immune response based on 60 genes (Table S4B). Additional TNF‐related genes TRAF1 and TNFRSF9 not identified by the above analysis but included in the set of 324 genes were added to the set to obtain a final list of 62 differentially expressed immune‐related genes. Unsupervised hierarchical clustering using Euclidean distance was executed in TMeV based on mean fold change with M*O*N‐DNJ treatment compared with untreated controls. The fifteen gene subset with strong early suppression in uninfected macrophages and ubiquitous suppression by 24 h p.i. was combined with a similarly identified gene set for cell fate and those genes involved in UPR, inflammation and cell fate determination (Figure 6), and this union was used to assemble a network in STRING‐DB.

### Detection of reactive oxygen species (ROS)

Macrophages were DENV‐ or mock‐infected and M*O*N‐DNJ‐treated as previously described. ROS levels were measured 18 h post‐infection for *n* = 3 donors in technical triplicate. Cells were washed once in PBS and stained with 5 μM CellROX Green reagent (Invitrogen, C10444) for 30 min at 37℃. Cells were then scraped and fixed in 4% paraformaldehyde for 15 min at 4℃. After a further washing step, cells were resuspended in PBS with 0·5% BSA and 5 mM EDTA and fluorescence was measured using a BD FACS Calibur. Cells were gated to exclude debris and doublets and geometric mean fluorescent intensity was recorded based on a minimum of 5000 gated events. Geometric mean fluorescence intensities (gMFI) were exported to Prism, normalized to uninfected, untreated controls, and then analysed by two‐way ANOVA for statistically significant differences.

### qRT‐PCR gene validation

Selected transcripts were assayed by qRT‐PCR to confirm differential regulation observed by microarray. Macrophages from *n* = 4 donors were mock‐infected, DENV‐infected or LPS‐treated and treated with control or 25 μM M*O*N‐DNJ (grey bars) in duplicate in identical fashion to samples generated for microarray. RNA was collected at 6 h and 24 h p.i. by TRIzol lysis and Direct‐zol (Zymogen) isolation as described above and stored at −80℃ prior to assay. Samples were thawed and added to Verso 1‐step RT‐qPCR kit (Thermo Fisher Scientific) with ROX, and amplification was monitored by fluorescence detection using an AB7500 Real‐Time PCR System (Thermo Fisher Scientific) as per the manufacturer's instructions. FAM‐MGB experimental primer/probe sets were obtained from Thermo Fisher Scientific and validated to have efficiency of 100 ± 10%. All experimental probes were normalized to relative quantity of RPLP2 VIC‐MGB endogenous control (Thermo Fisher Scientific) based on observed homogeneity of RPLP2 levels across all treatments in transcriptomic experiments. Experimental samples were normalized to relative quantity of transcript at the equivalent time point for uninfected, untreated samples. Holm–Šídák correction for multiple comparisons was performed to identify statistically significant differences for pairwise comparisons.

### Statistical analyses

Specific statistical tests used to evaluate for significance are described in the appropriate figure legend. In general, parametric tests were used where possible with post hoc pairwise testing using Holm–Šídák correction for multiple comparisons. Where assumptions of normality were not met, equivalent non‐parametric tests on rank were used with Tukey testing for multiple comparisons. All transcriptomic data handling was performed in R as described in the associated section of the methods. All further data handling and statistical analyses were conducted in SigmaPlot and GraphPad Prism.

## CONFLICT OF INTEREST

The authors declare no competing interests.

## AUTHOR CONTRIBUTIONS

A.C.S. designed the study, conducted experiments, analysed data and co‐wrote the manuscript. F.O.M. designed and performed analyses and co‐wrote the manuscript. B.E.T. performed experiments and edited the manuscript. N.P. performed experiments and edited the manuscript. M.L.H. performed experiments. R.A.D. designed the study and edited the manuscript. J.L.M. designed the study, conducted experiments and co‐wrote the manuscript. N.Z. designed the study and co‐wrote the manuscript.

## Supporting information

Fig S1

Fig S2

Fig S3

Table S1

Table S2

Table S3

Table S4

Table S5

Table S6

Table S7
