# Supplementary material for: Pathogen‐induced inflammation is attenuated by the iminosugar MON‐DNJ via modulation of the unfolded protein response
Source: Immunology. 2021 Aug 1;164(3):587–601. doi: 10.1111/imm.13393 (PMC8517592; doi:10.1111/imm.13393)
Supplement: Supplementary file 1 — Fig S1 [file IMM-164-587-s008.pdf]

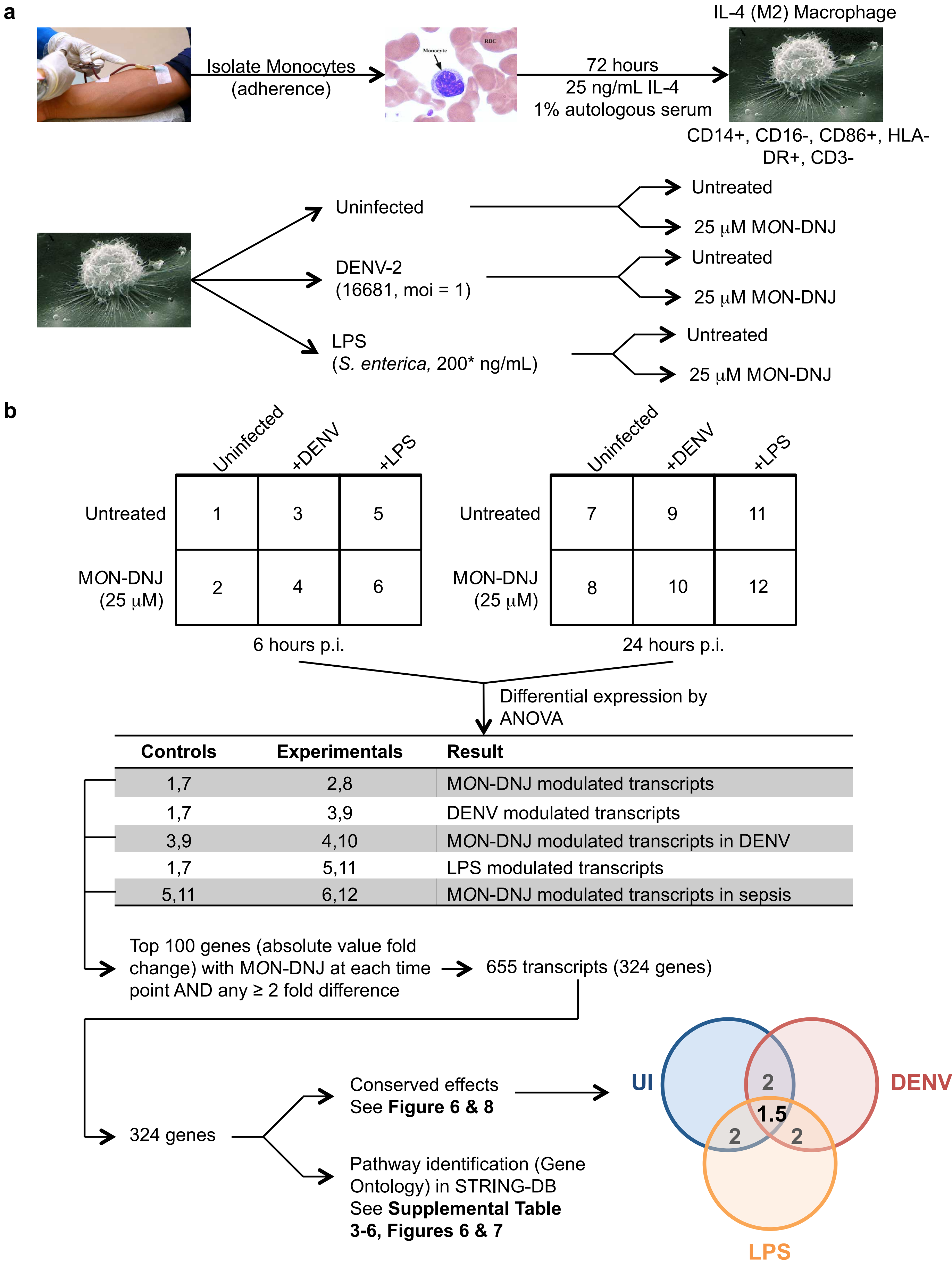
a
Isolate Monocytes
(adherence)
Monocyte
RBC
72 hours
25 ng/mL IL-4
1% autologous serum
IL-4 (M2) Macrophage
CD14+, CD16-, CD86+, HLA-DR+, CD3-
Uninfected
Untreated
25 µM MON-DNJ
DENV-2
(16681, moi = 1)
Untreated
25 µM MON-DNJ
LPS
(*S. enterica,* 200* ng/mL)
Untreated
25 µM MON-DNJ
b
Uninfected
+DENV
+LPS
Untreated
1
3
5
MON-DNJ
(25 µM)
2
4
6
6 hours p.i.
Uninfected
+DENV
+LPS
Untreated
7
9
11
MON-DNJ
(25 µM)
8
10
12
24 hours p.i.
Differential expression by ANOVA
Controls
Experimentals
Result
1,7
2,8
MON-DNJ modulated transcripts
1,7
3,9
DENV modulated transcripts
3,9
4,10
MON-DNJ modulated transcripts in DENV
1,7
5,11
LPS modulated transcripts
5,11
6,12
MON-DNJ modulated transcripts in sepsis
Top 100 genes (absolute value fold change) with MON-DNJ at each time point AND any ≥ 2 fold difference
655 transcripts (324 genes)
324 genes
Conserved effects
See **Figure 6 & 8**
Pathway identification (Gene Ontology) in STRING-DB
See **Supplemental Table 3-6, Figures 6 & 7**
UI
DENV
LPS
2
1.5
2
2

Supplemental Figure S1 Macrophage model and treatment descriptions. (a) Human monocytes were isolated from fresh blood by adherence to bovine gelatin coated plates and cultured for 3 days in the presence of 1% autologous human plasma and the Th2 cytokine IL-4. IL-4 induces gene and protein expression programs previously referred to as alternative activation or M2 activation (20–22). After 72 hours of IL-4 stimulation, cells were infected with DENV at a MOI 1, treated with LPS from *Salmonella enterica* at 200 ng/mL, or mock infected with replacement of media only (uninfected, UI). After 90 minutes in the presence of pathogen, the iminosugar M*O*N-DNJ was added for the remainder of treatment duration at a final concentration of 25 µM. LPS was diluted by the addition of drug to a final concentration of 100 ng/mL for the remainder of the treatment duration. Supernatants were collected at indicated time points for measurement of cytokine and viral titre. Cell lysates were collected for measurement of reactive oxygen species (ROS) and RNA transcripts. (b) Transcriptomics data were processed as described in the Methods to identify 324 genes differentially expressed in response to M*O*N-DNJ in at least one sample. Conserved changes were evaluated from the union set by identifying the intersection of genes at 6 hours with at least 2-fold change in 2 of 3 infection states or 1.5 fold change in all 3 infection states (see **Fig. 6 and Fig. 8**). An identical process was applied at 24 hours and yielded only 13 unique genes (**Supplemental Table S4A**).
