# Supplementary material for: Pathogen‐induced inflammation is attenuated by the iminosugar MON‐DNJ via modulation of the unfolded protein response
Source: Immunology. 2021 Aug 1;164(3):587–601. doi: 10.1111/imm.13393 (PMC8517592; doi:10.1111/imm.13393)
Supplement: Supplementary file 2 — Fig S2 [file IMM-164-587-s009.pdf]

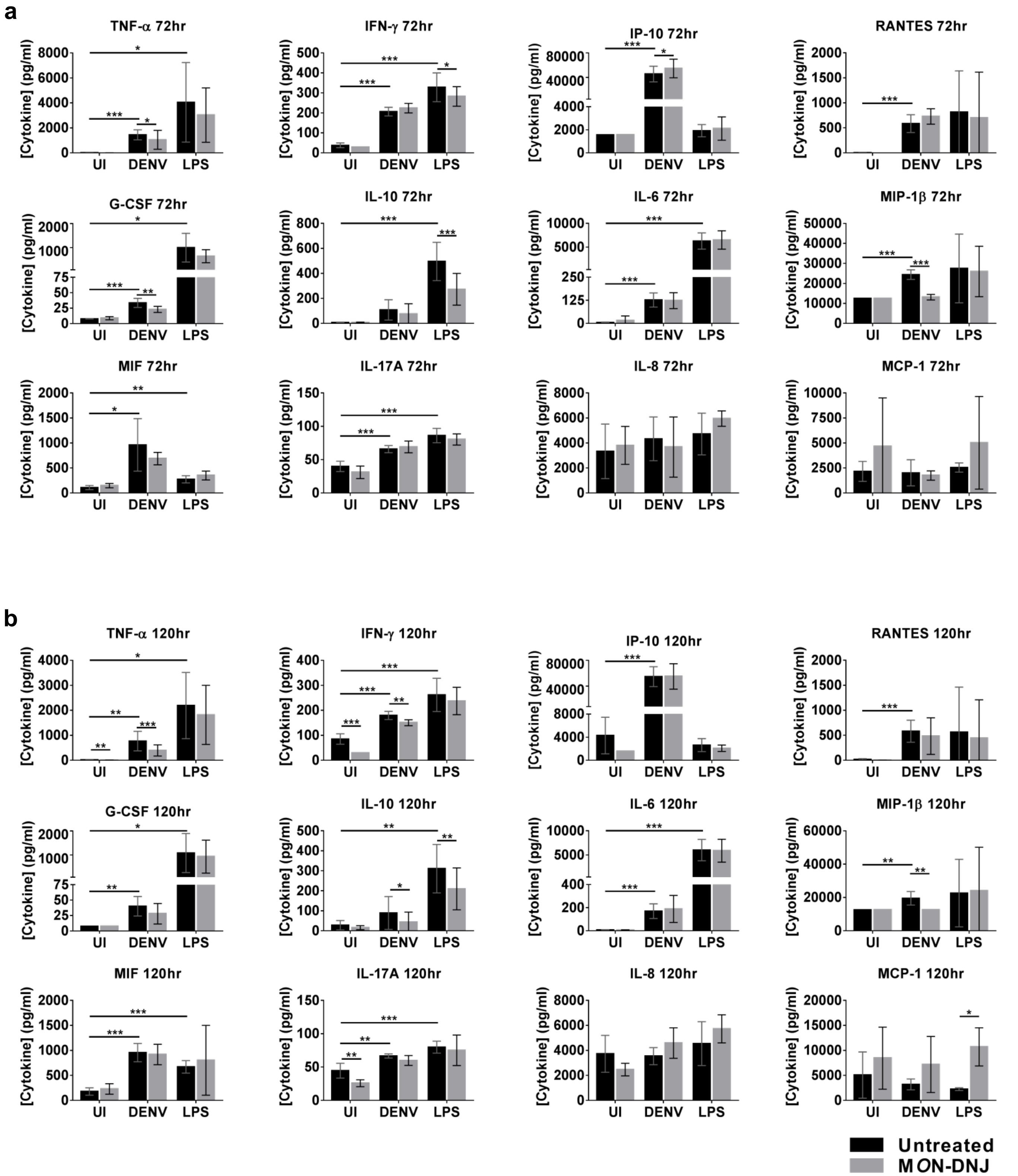

Supplemental Figure S2 Sustained reduction of cytokine by M*O*N-DNJ is target specific. Cytokines were assayed on a Luminex platform and analyzed for statistically significant differences as described for **Fig. 1** with collection at 72 hours (a) and 120 hours (b) p.i. for the same donors. Biological replicates (n=5) were assayed in technical singlicate. Discontinuous axes are used where necessary as a consequence of > 10 fold difference in level of cytokine induced by DENV and LPS. M*O*N-DNJ (grey bars) concentration is 25 µM. All error bars represent standard deviation. * $p < 0.05$, ** $p < 0.01$, *** $p < 0.001$
