# Supplementary material for: Pathogen‐induced inflammation is attenuated by the iminosugar MON‐DNJ via modulation of the unfolded protein response
Source: Immunology. 2021 Aug 1;164(3):587–601. doi: 10.1111/imm.13393 (PMC8517592; doi:10.1111/imm.13393)
Supplement: Supplementary file 3 — Fig S3 [file IMM-164-587-s007.pdf]

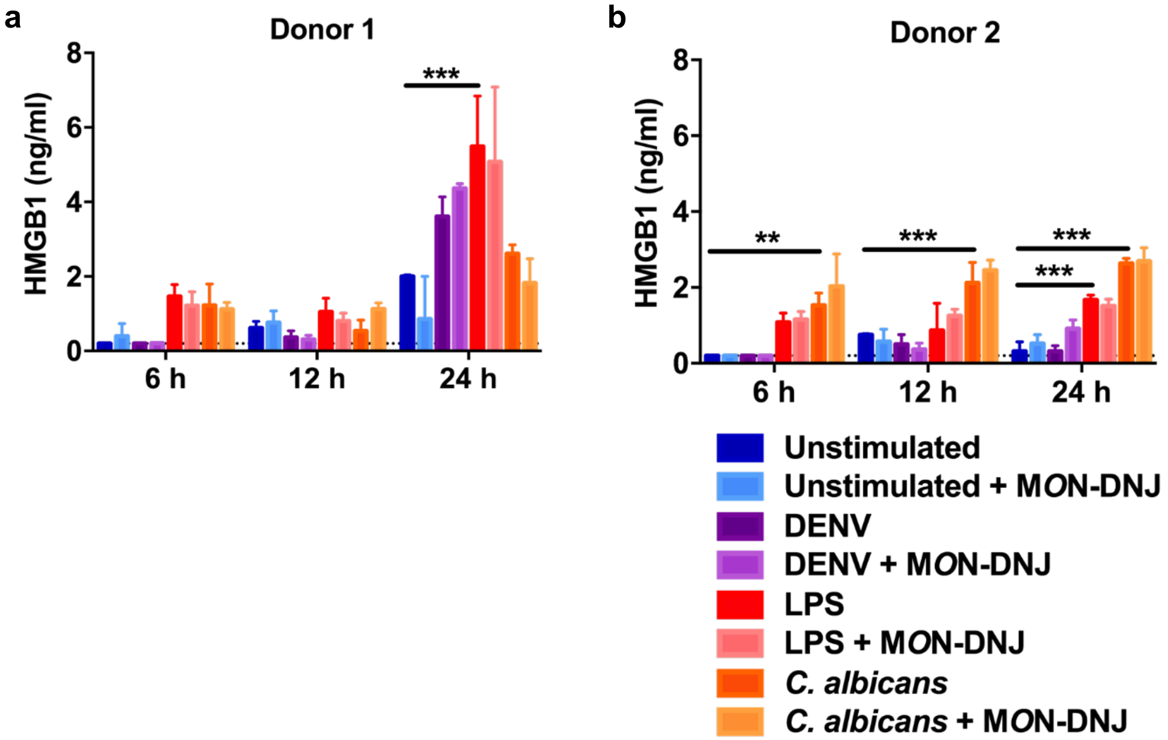

Supplemental Figure S3 HMGB1 secretion is induced by LPS and heat-killed *C. albicans*, but M*O*N-DNJ does not affect levels. Two donors (a, b) were treated as previously described with infectious stimuli +/- 25 µM M*O*N-DNJ for 6, 12, and 24 hour time points. Supernatants were collected for HMGB1 ELISA as described in the Methods. Each sample was assayed in technical triplicate (n=3). Repeated Measures Two way ANOVA with Holm-Šídák correction for multiple comparisons was performed to identify statistically significant differences. All error bars represent standard deviation. * $p < 0.05$, ** $p < 0.01$, *** $p < 0.001$
