# Supplementary material for: Pathogen‐induced inflammation is attenuated by the iminosugar MON‐DNJ via modulation of the unfolded protein response
Source: Immunology. 2021 Aug 1;164(3):587–601. doi: 10.1111/imm.13393 (PMC8517592; doi:10.1111/imm.13393)
Supplement: Supplementary file 4 — Table S1 [file IMM-164-587-s010.pdf]

Supplemental Table 1 Statistical significance of differential cytokine expression

TNF-α

	Infectious regulation			Drug Effects in Uninfected			Drug Effects in DENV			Druge Effects in LPS		
	Control	Experimental	*p* value	Control	Experimental	*p* value	Control	Experimental	*p* value	Control	Experimental	*p* value
Day 1	Uninf/untreat/d1	DENV/Unt/d1	0.005839	Uninf/untreat/d1	Uninf/NBDNJ/d1	ns	DENV/untreat/d1	DENV/NBDNJ/d1	0.021	LPS/untreat/d1	LPS/NBDNJ/d1	ns
		LPS/Unt/d1	0.027174		Uninf/MONDNJ/d1	ns		DENV/MONDNJ/d1	0.005		LPS/MONDNJ/d1	0.049
					Uninf/NBDGJ/d1	ns		DENV/NBDGJ/d1	ns		LPS/NBDGJ/d1	ns
Day 3	Uninf/untreat/d3	DENV/Unt/d3	0.000132	Uninf/untreat/d3	Uninf/NBDNJ/d3	ns	DENV/untreat/d3	DENV/NBDNJ/d3	ns	LPS/untreat/d3	LPS/NBDNJ/d3	ns
		LPS/Unt/d3	0.027174		Uninf/MONDNJ/d3	ns		DENV/MONDNJ/d3	<0.05		LPS/MONDNJ/d3	ns
					Uninf/NBDGJ/d3	ns		DENV/NBDGJ/d3	ns		LPS/NBDGJ/d3	ns
Day 5	Uninf/untreat/d5	DENV/Unt/d5	0.005334	Uninf/untreat/d5	Uninf/NBDNJ/d5	0.002	DENV/untreat/d5	DENV/NBDNJ/d5	ns	LPS/untreat/d5	LPS/NBDNJ/d5	ns
		LPS/Unt/d5	0.018838		Uninf/MONDNJ/d5	0.001		DENV/MONDNJ/d5	0.003		LPS/MONDNJ/d5	ns
					Uninf/NBDGJ/d5	ns		DENV/NBDGJ/d5	ns		LPS/NBDGJ/d5	ns

IFN-γ

	Infectious regulation			Drug Effects in Uninfected			Drug Effects in DENV			Druge Effects in LPS		
	Control	Experimental	*p* value	Control	Experimental	*p* value	Control	Experimental	*p* value	Control	Experimental	*p* value
Day 1	Uninf/untreat/d1	DENV/Unt/d1	0.000306	Uninf/untreat/d1	Uninf/NBDNJ/d1	ns	DENV/untreat/d1	DENV/NBDNJ/d1	ns	LPS/untreat/d1	LPS/NBDNJ/d1	ns
		LPS/Unt/d1	0.00001		Uninf/MONDNJ/d1	ns		DENV/MONDNJ/d1	<0.001		LPS/MONDNJ/d1	<0.001
					Uninf/NBDGJ/d1	ns		DENV/NBDGJ/d1	0.04		LPS/NBDGJ/d1	ns
Day 3	Uninf/untreat/d3	DENV/Unt/d3	<0.000001	Uninf/untreat/d3	Uninf/NBDNJ/d3	ns	DENV/untreat/d3	DENV/NBDNJ/d3	ns	LPS/untreat/d3	LPS/NBDNJ/d3	ns
		LPS/Unt/d3	0.000041		Uninf/MONDNJ/d3	ns		DENV/MONDNJ/d3	ns		LPS/MONDNJ/d3	0.049
					Uninf/NBDGJ/d3	ns		DENV/NBDGJ/d3	ns		LPS/NBDGJ/d3	ns
Day 5	Uninf/untreat/d5	DENV/Unt/d5	0.00009	Uninf/untreat/d5	Uninf/NBDNJ/d5	<0.001	DENV/untreat/d5	DENV/NBDNJ/d5	ns	LPS/untreat/d5	LPS/NBDNJ/d5	ns
		LPS/Unt/d5	0.000472		Uninf/MONDNJ/d5	<0.001		DENV/MONDNJ/d5	0.005		LPS/MONDNJ/d5	ns
					Uninf/NBDGJ/d5	ns		DENV/NBDGJ/d5	ns		LPS/NBDGJ/d5	ns

IP-10

	Infectious regulation			Drug Effects in Uninfected			Drug Effects in DENV			Druge Effects in LPS		
	Control	Experimental	*p* value	Control	Experimental	*p* value	Control	Experimental	*p* value	Control	Experimental	*p* value
Day 1	Uninf/untreat/d1	DENV/Unt/d1	0.007363	Uninf/untreat/d1	Uninf/NBDNJ/d1	ns	DENV/untreat/d1	DENV/NBDNJ/d1	0.001	LPS/untreat/d1	LPS/NBDNJ/d1	ns
		LPS/Unt/d1	0.457949		Uninf/MONDNJ/d1	ns		DENV/MONDNJ/d1	<0.001		LPS/MONDNJ/d1	ns
					Uninf/NBDGJ/d1	ns		DENV/NBDGJ/d1	ns		LPS/NBDGJ/d1	ns
Day 3	Uninf/untreat/d3	DENV/Unt/d3	0.000189	Uninf/untreat/d3	Uninf/NBDNJ/d3	ns	DENV/untreat/d3	DENV/NBDNJ/d3	ns	LPS/untreat/d3	LPS/NBDNJ/d3	ns
		LPS/Unt/d3	0.420332		Uninf/MONDNJ/d3	ns		DENV/MONDNJ/d3	0.02		LPS/MONDNJ/d3	ns
					Uninf/NBDGJ/d3	ns		DENV/NBDGJ/d3	ns		LPS/NBDGJ/d3	ns
Day 5	Uninf/untreat/d5	DENV/Unt/d5	0.000197	Uninf/untreat/d5	Uninf/NBDNJ/d5	ns	DENV/untreat/d5	DENV/NBDNJ/d5	ns	LPS/untreat/d5	LPS/NBDNJ/d5	ns
		LPS/Unt/d5	0.457949		Uninf/MONDNJ/d5	ns		DENV/MONDNJ/d5	ns		LPS/MONDNJ/d5	ns
					Uninf/NBDGJ/d5	ns		DENV/NBDGJ/d5	ns		LPS/NBDGJ/d5	ns

RANTES

	Infectious regulation			Drug Effects in Uninfected			Drug Effects in DENV			Druge Effects in LPS		
	Control	Experimental	*p* value	Control	Experimental	*p* value	Control	Experimental	*p* value	Control	Experimental	*p* value
Day 1	Uninf/untreat/d1	DENV/Unt/d1	0.155013	Uninf/untreat/d1	Uninf/NBDNJ/d1	ns	DENV/untreat/d1	DENV/NBDNJ/d1	ns	LPS/untreat/d1	LPS/NBDNJ/d1	ns
		LPS/Unt/d1	0.000839		Uninf/MONDNJ/d1	ns		DENV/MONDNJ/d1	0.032		LPS/MONDNJ/d1	0.013
					Uninf/NBDGJ/d1	ns		DENV/NBDGJ/d1	ns		LPS/NBDGJ/d1	ns
Day 3	Uninf/untreat/d3	DENV/Unt/d3	0.000253	Uninf/untreat/d3	Uninf/NBDNJ/d3	ns	DENV/untreat/d3	DENV/NBDNJ/d3	ns	LPS/untreat/d3	LPS/NBDNJ/d3	ns
		LPS/Unt/d3	0.112984		Uninf/MONDNJ/d3	ns		DENV/MONDNJ/d3	ns		LPS/MONDNJ/d3	ns
					Uninf/NBDGJ/d3	ns		DENV/NBDGJ/d3	ns		LPS/NBDGJ/d3	ns
Day 5	Uninf/untreat/d5	DENV/Unt/d5	0.000937	Uninf/untreat/d5	Uninf/NBDNJ/d5	ns	DENV/untreat/d5	DENV/NBDNJ/d5	ns	LPS/untreat/d5	LPS/NBDNJ/d5	ns
		LPS/Unt/d5	0.213855		Uninf/MONDNJ/d5	ns		DENV/MONDNJ/d5	ns		LPS/MONDNJ/d5	ns
					Uninf/NBDGJ/d5	ns		DENV/NBDGJ/d5	ns		LPS/NBDGJ/d5	ns

G-CSF

	Infectious regulation			Drug Effects in Uninfected			Drug Effects in DENV			Druge Effects in LPS		
	Control	Experimental	*p* value	Control	Experimental	*p* value	Control	Experimental	*p* value	Control	Experimental	*p* value
Day 1	Uninf/untreat/d1	DENV/Unt/d1	0.17618	Uninf/untreat/d1	Uninf/NBDNJ/d1	ns	DENV/untreat/d1	DENV/NBDNJ/d1	ns	LPS/untreat/d1	LPS/NBDNJ/d1	ns
		LPS/Unt/d1	0.003153		Uninf/MONDNJ/d1	ns		DENV/MONDNJ/d1	ns		LPS/MONDNJ/d1	0.015
					Uninf/NBDGJ/d1	ns		DENV/NBDGJ/d1	ns		LPS/NBDGJ/d1	ns
Day 3	Uninf/untreat/d3	DENV/Unt/d3	0.000146	Uninf/untreat/d3	Uninf/NBDNJ/d3	ns	DENV/untreat/d3	DENV/NBDNJ/d3	ns	LPS/untreat/d3	LPS/NBDNJ/d3	ns
		LPS/Unt/d3	0.011887		Uninf/MONDNJ/d3	ns		DENV/MONDNJ/d3	0.008		LPS/MONDNJ/d3	ns
					Uninf/NBDGJ/d3	ns		DENV/NBDGJ/d3	ns		LPS/NBDGJ/d3	ns
Day 5	Uninf/untreat/d5	DENV/Unt/d5	0.003473	Uninf/untreat/d5	Uninf/NBDNJ/d5	ns	DENV/untreat/d5	DENV/NBDNJ/d5	ns	LPS/untreat/d5	LPS/NBDNJ/d5	ns
		LPS/Unt/d5	0.018525		Uninf/MONDNJ/d5	ns		DENV/MONDNJ/d5	ns		LPS/MONDNJ/d5	ns
					Uninf/NBDGJ/d5	ns		DENV/NBDGJ/d5	ns		LPS/NBDGJ/d5	ns

IL-10

	Infectious regulation			Drug Effects in Uninfected			Drug Effects in DENV			Druge Effects in LPS		
	Control	Experimental	*p* value	Control	Experimental	*p* value	Control	Experimental	*p* value	Control	Experimental	*p* value
Day 1	Uninf/untreat/d1	DENV/Unt/d1	0.257635	Uninf/untreat/d1	Uninf/NBDNJ/d1	ns	DENV/untreat/d1	DENV/NBDNJ/d1	ns	LPS/untreat/d1	LPS/NBDNJ/d1	ns
		LPS/Unt/d1	0.001498		Uninf/MONDNJ/d1	ns		DENV/MONDNJ/d1	0.048		LPS/MONDNJ/d1	<0.001
					Uninf/NBDGJ/d1	ns		DENV/NBDGJ/d1	ns		LPS/NBDGJ/d1	ns
Day 3	Uninf/untreat/d3	DENV/Unt/d3	0.07038	Uninf/untreat/d3	Uninf/NBDNJ/d3	ns	DENV/untreat/d3	DENV/NBDNJ/d3	ns	LPS/untreat/d3	LPS/NBDNJ/d3	ns
		LPS/Unt/d3	0.000297		Uninf/MONDNJ/d3	ns		DENV/MONDNJ/d3	ns		LPS/MONDNJ/d3	<0.001
					Uninf/NBDGJ/d3	ns		DENV/NBDGJ/d3	ns		LPS/NBDGJ/d3	ns
Day 5	Uninf/untreat/d5	DENV/Unt/d5	0.257635	Uninf/untreat/d5	Uninf/NBDNJ/d5	ns	DENV/untreat/d5	DENV/NBDNJ/d5	ns	LPS/untreat/d5	LPS/NBDNJ/d5	ns
		LPS/Unt/d5	0.001498		Uninf/MONDNJ/d5	ns		DENV/MONDNJ/d5	0.04		LPS/MONDNJ/d5	0.006
					Uninf/NBDGJ/d5	ns		DENV/NBDGJ/d5	ns		LPS/NBDGJ/d5	ns

IL-6

	Infectious regulation			Drug Effects in Uninfected			Drug Effects in DENV			Druge Effects in LPS		
	Control	Experimental	*p* value	Control	Experimental	*p* value	Control	Experimental	*p* value	Control	Experimental	*p* value
Day 1	Uninf/untreat/d1	DENV/Unt/d1	0.272923	Uninf/untreat/d1	Uninf/NBDNJ/d1	ns	DENV/untreat/d1	DENV/NBDNJ/d1	ns	LPS/untreat/d1	LPS/NBDNJ/d1	ns
		LPS/Unt/d1	<0.000001		Uninf/MONDNJ/d1	ns		DENV/MONDNJ/d1	ns		LPS/MONDNJ/d1	0.003
					Uninf/NBDGJ/d1	ns		DENV/NBDGJ/d1	ns		LPS/NBDGJ/d1	ns
Day 3	Uninf/untreat/d3	DENV/Unt/d3	0.00031	Uninf/untreat/d3	Uninf/NBDNJ/d3	ns	DENV/untreat/d3	DENV/NBDNJ/d3	ns	LPS/untreat/d3	LPS/NBDNJ/d3	ns
		LPS/Unt/d3	0.000069		Uninf/MONDNJ/d3	ns		DENV/MONDNJ/d3	ns		LPS/MONDNJ/d3	ns
					Uninf/NBDGJ/d3	ns		DENV/NBDGJ/d3	ns		LPS/NBDGJ/d3	ns
Day 5	Uninf/untreat/d5	DENV/Unt/d5	0.000927	Uninf/untreat/d5	Uninf/NBDNJ/d5	ns	DENV/untreat/d5	DENV/NBDNJ/d5	ns	LPS/untreat/d5	LPS/NBDNJ/d5	ns
		LPS/Unt/d5	0.000303		Uninf/MONDNJ/d5	ns		DENV/MONDNJ/d5	ns		LPS/MONDNJ/d5	ns
					Uninf/NBDGJ/d5	ns		DENV/NBDGJ/d5	ns		LPS/NBDGJ/d5	ns

MIP-1β

	Infectious regulation			Drug Effects in Uninfected			Drug Effects in DENV			Druge Effects in LPS		
	Control	Experimental	*p* value	Control	Experimental	*p* value	Control	Experimental	*p* value	Control	Experimental	*p* value
Day 1	Uninf/untreat/d1	DENV/Unt/d1	ns	Uninf/untreat/d1	Uninf/NBDNJ/d1	ns	DENV/untreat/d1	DENV/NBDNJ/d1	ns	LPS/untreat/d1	LPS/NBDNJ/d1	ns
		LPS/Unt/d1	0.006119		Uninf/MONDNJ/d1	ns		DENV/MONDNJ/d1	ns		LPS/MONDNJ/d1	ns
					Uninf/NBDGJ/d1	ns		DENV/NBDGJ/d1	ns		LPS/NBDGJ/d1	ns
Day 3	Uninf/untreat/d3	DENV/Unt/d3	0.000007	Uninf/untreat/d3	Uninf/NBDNJ/d3	ns	DENV/untreat/d3	DENV/NBDNJ/d3	<0.001	LPS/untreat/d3	LPS/NBDNJ/d3	ns
		LPS/Unt/d3	0.16659		Uninf/MONDNJ/d3	ns		DENV/MONDNJ/d3	<0.001		LPS/MONDNJ/d3	ns
					Uninf/NBDGJ/d3	ns		DENV/NBDGJ/d3	0.003		LPS/NBDGJ/d3	ns
Day 5	Uninf/untreat/d5	DENV/Unt/d5	0.004759	Uninf/untreat/d5	Uninf/NBDNJ/d5	ns	DENV/untreat/d5	DENV/NBDNJ/d5	0.001	LPS/untreat/d5	LPS/NBDNJ/d5	ns
		LPS/Unt/d5	0.295728		Uninf/MONDNJ/d5	ns		DENV/MONDNJ/d5	0.002		LPS/MONDNJ/d5	ns
					Uninf/NBDGJ/d5	ns		DENV/NBDGJ/d5	ns		LPS/NBDGJ/d5	ns

MIF

	Infectious regulation			Drug Effects in Uninfected			Drug Effects in DENV			Druge Effects in LPS		
	Control	Experimental	*p* value	Control	Experimental	*p* value	Control	Experimental	*p* value	Control	Experimental	*p* value
Day 1	Uninf/untreat/d1	DENV/Unt/d1	0.294993	Uninf/untreat/d1	Uninf/NBDNJ/d1	ns	DENV/untreat/d1	DENV/NBDNJ/d1	ns	LPS/untreat/d1	LPS/NBDNJ/d1	ns
		LPS/Unt/d1	0.062659		Uninf/MONDNJ/d1	ns		DENV/MONDNJ/d1	ns		LPS/MONDNJ/d1	ns
					Uninf/NBDGJ/d1	ns		DENV/NBDGJ/d1	ns		LPS/NBDGJ/d1	ns
Day 3	Uninf/untreat/d3	DENV/Unt/d3	0.013415	Uninf/untreat/d3	Uninf/NBDNJ/d3	ns	DENV/untreat/d3	DENV/NBDNJ/d3	ns	LPS/untreat/d3	LPS/NBDNJ/d3	ns
		LPS/Unt/d3	0.004173		Uninf/MONDNJ/d3	ns		DENV/MONDNJ/d3	ns		LPS/MONDNJ/d3	ns
					Uninf/NBDGJ/d3	ns		DENV/NBDGJ/d3	ns		LPS/NBDGJ/d3	ns
Day 5	Uninf/untreat/d5	DENV/Unt/d5	0.000066	Uninf/untreat/d5	Uninf/NBDNJ/d5	ns	DENV/untreat/d5	DENV/NBDNJ/d5	ns	LPS/untreat/d5	LPS/NBDNJ/d5	ns
		LPS/Unt/d5	0.000187		Uninf/MONDNJ/d5	ns		DENV/MONDNJ/d5	ns		LPS/MONDNJ/d5	ns
					Uninf/NBDGJ/d5	ns		DENV/NBDGJ/d5	ns		LPS/NBDGJ/d5	ns

IL-17A

	Infectious regulation			Drug Effects in Uninfected			Drug Effects in DENV			Druge Effects in LPS		
	Control	Experimental	*p* value	Control	Experimental	*p* value	Control	Experimental	*p* value	Control	Experimental	*p* value
Day 1	Uninf/untreat/d1	DENV/Unt/d1	0.119493	Uninf/untreat/d1	Uninf/NBDNJ/d1	ns	DENV/untreat/d1	DENV/NBDNJ/d1	ns	LPS/untreat/d1	LPS/NBDNJ/d1	ns
		LPS/Unt/d1	0.000082		Uninf/MONDNJ/d1	ns		DENV/MONDNJ/d1	ns		LPS/MONDNJ/d1	ns
					Uninf/NBDGJ/d1	ns		DENV/NBDGJ/d1	ns		LPS/NBDGJ/d1	ns
Day 3	Uninf/untreat/d3	DENV/Unt/d3	0.000823	Uninf/untreat/d3	Uninf/NBDNJ/d3	ns	DENV/untreat/d3	DENV/NBDNJ/d3	ns	LPS/untreat/d3	LPS/NBDNJ/d3	ns
		LPS/Unt/d3	0.000101		Uninf/MONDNJ/d3	ns		DENV/MONDNJ/d3	ns		LPS/MONDNJ/d3	ns
					Uninf/NBDGJ/d3	ns		DENV/NBDGJ/d3	ns		LPS/NBDGJ/d3	ns
Day 5	Uninf/untreat/d5	DENV/Unt/d5	0.005407	Uninf/untreat/d5	Uninf/NBDNJ/d5	0.003	DENV/untreat/d5	DENV/NBDNJ/d5	ns	LPS/untreat/d5	LPS/NBDNJ/d5	ns
		LPS/Unt/d5	0.000548		Uninf/MONDNJ/d5	0.003		DENV/MONDNJ/d5	ns		LPS/MONDNJ/d5	ns
					Uninf/NBDGJ/d5	ns		DENV/NBDGJ/d5	ns		LPS/NBDGJ/d5	ns

IL-8

	Infectious regulation			Drug Effects in Uninfected			Drug Effects in DENV			Druge Effects in LPS		
	Control	Experimental	*p* value	Control	Experimental	*p* value	Control	Experimental	*p* value	Control	Experimental	*p* value
Day 1	Uninf/untreat/d1	DENV/Unt/d1	0.748544	Uninf/untreat/d1	Uninf/NBDNJ/d1	ns	DENV/untreat/d1	DENV/NBDNJ/d1	ns	LPS/untreat/d1	LPS/NBDNJ/d1	ns
		LPS/Unt/d1	0.125207		Uninf/MONDNJ/d1	ns		DENV/MONDNJ/d1	ns		LPS/MONDNJ/d1	ns
					Uninf/NBDGJ/d1	ns		DENV/NBDGJ/d1	ns		LPS/NBDGJ/d1	ns
Day 3	Uninf/untreat/d3	DENV/Unt/d3	0.748544	Uninf/untreat/d3	Uninf/NBDNJ/d3	ns	DENV/untreat/d3	DENV/NBDNJ/d3	ns	LPS/untreat/d3	LPS/NBDNJ/d3	ns
		LPS/Unt/d3	0.499502		Uninf/MONDNJ/d3	ns		DENV/MONDNJ/d3	ns		LPS/MONDNJ/d3	ns
					Uninf/NBDGJ/d3	ns		DENV/NBDGJ/d3	ns		LPS/NBDGJ/d3	ns
Day 5	Uninf/untreat/d5	DENV/Unt/d5	0.803446	Uninf/untreat/d5	Uninf/NBDNJ/d5	ns	DENV/untreat/d5	DENV/NBDNJ/d5	ns	LPS/untreat/d5	LPS/NBDNJ/d5	ns
		LPS/Unt/d5	0.499502		Uninf/MONDNJ/d5	ns		DENV/MONDNJ/d5	ns		LPS/MONDNJ/d5	ns
					Uninf/NBDGJ/d5	ns		DENV/NBDGJ/d5	ns		LPS/NBDGJ/d5	ns

MCP1

	Infectious regulation			Drug Effects in Uninfected			Drug Effects in DENV			Druge Effects in LPS		
	Control	Experimental	*p* value	Control	Experimental	*p* value	Control	Experimental	*p* value	Control	Experimental	*p* value
Day 1	Uninf/untreat/d1	DENV/Unt/d1	0.977149	Uninf/untreat/d1	Uninf/NBDNJ/d1	ns	DENV/untreat/d1	DENV/NBDNJ/d1	ns	LPS/untreat/d1	LPS/NBDNJ/d1	ns
		LPS/Unt/d1	0.450188		Uninf/MONDNJ/d1	ns		DENV/MONDNJ/d1	ns		LPS/MONDNJ/d1	ns
					Uninf/NBDGJ/d1	ns		DENV/NBDGJ/d1	ns		LPS/NBDGJ/d1	ns
Day 3	Uninf/untreat/d3	DENV/Unt/d3	0.977149	Uninf/untreat/d3	Uninf/NBDNJ/d3	ns	DENV/untreat/d3	DENV/NBDNJ/d3	ns	LPS/untreat/d3	LPS/NBDNJ/d3	ns
		LPS/Unt/d3	0.451886		Uninf/MONDNJ/d3	ns		DENV/MONDNJ/d3	ns		LPS/MONDNJ/d3	ns
					Uninf/NBDGJ/d3	ns		DENV/NBDGJ/d3	ns		LPS/NBDGJ/d3	ns
Day 5	Uninf/untreat/d5	DENV/Unt/d5	0.788497	Uninf/untreat/d5	Uninf/NBDNJ/d5	ns	DENV/untreat/d5	DENV/NBDNJ/d5	ns	LPS/untreat/d5	LPS/NBDNJ/d5	ns
		LPS/Unt/d5	0.450188		Uninf/MONDNJ/d5	ns		DENV/MONDNJ/d5	ns		LPS/MONDNJ/d5	0.012
					Uninf/NBDGJ/d5	ns		DENV/NBDGJ/d5	ns		LPS/NBDGJ/d5	ns
