# Supplementary material for: Pathogen‐induced inflammation is attenuated by the iminosugar MON‐DNJ via modulation of the unfolded protein response
Source: Immunology. 2021 Aug 1;164(3):587–601. doi: 10.1111/imm.13393 (PMC8517592; doi:10.1111/imm.13393)
Supplement: Supplementary file 5 — Table S2 [file IMM-164-587-s004.pdf]

**Supplemental Table 2 Differentially expressed genes with MON-DNJ**

| Gene Symbol | Gene Name |
|---|---|
| AARS | alanyl-tRNA synthetase |
| ABAT | 4-aminobutyrate aminotransferase |
| ACN9 | ACN9 homolog (S. cerevisiae) |
| ACP5 | Acid phosphatase 5, tartrate resistant |
| ADA | Adenosine deaminase |
| ADI1 | Acireductone dioxygenase 1 |
| AGPAT9 | 1-acylglycerol-3-phosphate O-acyltransferase 9 |
| ALDH1L2 | Aldehyde dehydrogenase 1 family, member L2 |
| ALDH5A1 | Aldehyde dehydrogenase 5 family, member A1 |
| ALG13 | Asparagine-linked glycosylation 13 homolog (S. cerevisiae) |
| ALK | Anaplastic lymphoma receptor tyrosine kinase |
| ALOX15B | Arachidonate 15-lipoxygenase, type B |
| AMY2B | Amylase, alpha 2B (pancreatic) |
| ANG | Angiogenin, ribonuclease, RNase A family, 5 |
| ANKRD1 | Ankyrin repeat domain 1 (cardiac muscle) |
| ANP32A | Acidic (leucine-rich) nuclear phosphoprotein 32 family, member A |
| APP | Amyloid beta (A4) precursor protein |
| ARHGEF10L | Rho guanine nucleotide exchange factor (GEF) 10-like |
| ARMCX2 | Armadillo repeat containing, X-linked 2 |
| ASNA1 | arsA arsenite transporter, ATP-binding, homolog 1 (bacterial) |
| ASNS | Asparagine synthetase (glutamine-hydrolyzing) |
| ATF3 | Activating transcription factor 3 |
| ATF4 | Activating transcription factor 4 (tax-responsive enhancer element B67) |
| ATF7IP2 | Activating transcription factor 7 interacting protein 2 |
| ATL1 | Atlastin GTPase 1 |
| ATMIN | ATM interactor |
| ATP1B1 | ATPase, Na+/K+ transporting, beta 1 polypeptide |
| ATP2A2 | ATPase, Ca++ transporting, cardiac muscle, slow twitch 2 |
| ATXN3 | Ataxin 3 |
| B3GNT5 | UDP-GlcNAc:betaGal beta-1,3-N-acetylglucosaminyltransferase 5 |
| BANP | BTG3 associated nuclear protein |
| BATF | Basic leucine zipper transcription factor, ATF-like |
| BCAS2 | Breast carcinoma amplified sequence 2 |
| BCL2A1 | BCL2-related protein A1 |
| BEX2 | Brain expressed X-linked 2 |
| BORA | Bora, aurora kinase A activator |
| C12orf23 | Chromosome 12 open reading frame 23 |
| C12orf65 | Chromosome 12 open reading frame 65 |
| C1D | C1D nuclear receptor corepressor |
| C1QTNF1 | C1q and tumor necrosis factor related protein 1 |
| C6orf62 | Chromosome 6 open reading frame 62 |
| CABLES2 | Cdk5 and Abl enzyme substrate 2 |
| CALR | Calreticulin |
| CAPZA1 | Capping protein (actin filament) muscle Z-line, alpha 1 |
| CARS | cysteinyl-tRNA synthetase |
| CAST | Calpastatin |
| CBWD1 | COBW domain containing 1 |
| CCDC167 | Coiled-coil domain containing 167 |
| CCDC28B | Coiled-coil domain containing 28B |
| CCDC60 | Coiled-coil domain containing 60 |
| CCL23 | Chemokine (C-C motif) ligand 23 |
| CCL4L2 | Chemokine (C-C motif) ligand 4-like 2 |
| CCNG2 | Cyclin G2 |
| CCPG1 | Cell cycle progression 1 |
| CD163 | CD163 molecule |
| CD164 | CD164 molecule, sialomucin |
| CD276 | CD276 molecule |
| CD36 | CD36 molecule (thrombospondin receptor) |
| CD38 | CD38 molecule |
| CDC42EP3 | CDC42 effector protein (Rho GTPase binding) 3 |
| CDK2AP2 | Cyclin-dependent kinase 2 associated protein 2 |
| CDR2 | Cerebellar degeneration-related protein 2, 62kDa |
| CDRT4 | CMT1A duplicated region transcript 4 |
| CHST2 | Carbohydrate (N-acetylglucosamine-6-O) sulfotransferase 2 |
| CISD2 | CDGSH iron sulfur domain 2 |
| CLIC2 | Chloride intracellular channel 2 |

| | |
|---|---|
| COMMD8 | COMM domain containing 8 |
| CRELD1 | Cysteine-rich with EGF-like domains 1 |
| CRELD2 | Cysteine-rich with EGF-like domains 2 |
| CTNNB1 | Catenin (cadherin-associated protein), beta 1, 88kDa |
| CTNS | Cystinosin, lysosomal cystine transporter |
| CTR9 | Ctr9, Paf1/RNA polymerase II complex component, homolog (S. cerevisiae) |
| CYB561 | Cytochrome b-561 |
| CYP27B1 | Cytochrome P450, family 27, subfamily B, polypeptide 1 |
| CYP51A1 | Cytochrome P450, family 51, subfamily A, polypeptide 1 |
| DDIT3 | DNA-damage-inducible transcript 3 |
| DDIT4 | DNA-damage-inducible transcript 4 |
| DDX17 | DEAD (Asp-Glu-Ala-Asp) box helicase 17 |
| DERL2 | Derlin 2 |
| DHRS11 | Dehydrogenase/reductase (SDR family) member 11 |
| DNAAF1 | Dynein, axonemal, assembly factor 1 |
| DNAJA4 | DnaJ (Hsp40) homolog, subfamily A, member 4 |
| DNAJB11 | DnaJ (Hsp40) homolog, subfamily B, member 11 |
| DNAJB5 | DnaJ (Hsp40) homolog, subfamily B, member 5 |
| DNAJB9 | DnaJ (Hsp40) homolog, subfamily B, member 9 |
| DNAJC10 | DnaJ (Hsp40) homolog, subfamily C, member 10 |
| DPCR1 | Diffuse panbronchiolitis critical region 1 |
| DPYD | Dihydropyrimidine dehydrogenase |
| DTX3L | Deltex 3-like (Drosophila) |
| DUSP6 | Dual specificity phosphatase 6 |
| DYNC1LI1 | Dynein, cytoplasmic 1, light intermediate chain 1 |
| DYRK2 | Dual-specificity tyrosine-(Y)-phosphorylation regulated kinase 2 |
| EBI3 | Epstein-Barr virus induced 3 |
| EEF1B2 | Eukaryotic translation elongation factor 1 beta 2 |
| EEPD1 | Endonuclease/exonuclease/phosphatase family domain containing 1 |
| EIF2AK3 | Eukaryotic translation initiation factor 2-alpha kinase 3 |
| ELOVL6 | ELOVL fatty acid elongase 6 |
| ENTPD7 | Ectonucleoside triphosphate diphosphohydrolase 7 |
| ERO1LB | ERO1-like beta (S. cerevisiae) |
| EVI5L | Ecotropic viral integration site 5-like |
| EVL | Enah/Vasp-like |
| EXT1 | Exostosin 1 |
| FABP5 | Fatty acid binding protein 5 (psoriasis-associated) |
| FAM129A | Family with sequence similarity 129, member A |
| FAM63A | Family with sequence similarity 63, member A |
| FAM70A | Family with sequence similarity 70, member A |
| FANCE | Fanconi anemia, complementation group E |
| FGFBP3 | Fibroblast growth factor binding protein 3 |
| FICD | FIC domain containing |
| FKBP11 | FK506 binding protein 11, 19 kDa |
| FKBP14 | FK506 binding protein 14, 22 kDa |
| FKBP2 | FK506 binding protein 2, 13kDa |
| FKBP4 | FK506 binding protein 4, 59kDa |
| FKBP5 | FK506 binding protein 5 |
| FNDC3A | Fibronectin type III domain containing 3A |
| FNDC3B | Fibronectin type III domain containing 3B |
| FPR1 | Formyl peptide receptor 1 |
| FPR3 | Formyl peptide receptor 3 |
| FZD2 | Frizzled family receptor 2 |
| G3BP1 | GTPase activating protein (SH3 domain) binding protein 1 |
| GABARAPL2 | GABA(A) receptor-associated protein-like 2 |
| GABRE | Gamma-aminobutyric acid (GABA) A receptor, epsilon |
| GADD45GIP1 | Growth arrest and DNA-damage-inducible, gamma interacting protein 1 |
| GCLC | Glutamate-cysteine ligase, catalytic subunit |
| GDF15 | Growth differentiation factor 15 |
| GFPT1 | Glutamine--fructose-6-phosphate transaminase 1 |
| GMPPB | GDP-mannose pyrophosphorylase B |
| GNB4 | Guanine nucleotide binding protein (G protein), beta polypeptide 4 |
| GNG10 | Guanine nucleotide binding protein (G protein), gamma 10 |
| GNL3 | Guanine nucleotide binding protein-like 3 (nucleolar) |
| GOT1 | Glutamic-oxaloacetic transaminase 1, soluble (aspartate aminotransferase 1) |
| GPR132 | G protein-coupled receptor 132 |
| GPR183 | G protein-coupled receptor 183 |
| GPT2 | Glutamic pyruvate transaminase (alanine aminotransferase) 2 |

| | |
|---|---|
| GRAMD1A | GRAM domain containing 1A |
| GYPC | Glycophorin C (Gerbich blood group) |
| HABP4 | Hyaluronan binding protein 4 |
| HCCS | Holocytochrome c synthase |
| HERPUD1 | Homocysteine-inducible, endoplasmic reticulum stress-inducible, ubiquitin-like domain member 1 |
| HIAT1 | Hippocampus abundant transcript 1 |
| HMGB1 | High mobility group box 1 |
| HRSP12 | Heat-responsive protein 12 |
| HSP90B1 | Heat shock protein 90kDa beta (Grp94), member 1 |
| HSPA13 | Heat shock protein 70kDa family, member 13 |
| HSPA1A | Heat shock 70kDa protein 1A |
| HSPA5 | Heat shock 70kDa protein 5 (glucose-regulated protein, 78kDa) |
| HSPH1 | Heat shock 105kDa/110kDa protein 1 |
| HTRA2 | HtrA serine peptidase 2 |
| HYOU1 | Hypoxia up-regulated 1 |
| ID2 | Inhibitor of DNA binding 2, dominant negative helix-loop-helix protein |
| IER3 | Immediate early response 3 |
| IRAK2 | Interleukin-1 receptor-associated kinase 2 |
| ISOC2 | Isochorismatase domain containing 2 |
| KCNK6 | Potassium channel, subfamily K, member 6 |
| KCTD15 | Potassium channel tetramerisation domain containing 15 |
| KIAA1033 | KIAA1033 |
| KIAA1279 | KIAA1279 |
| KLF4 | Kruppel-like factor 4 (gut) |
| KPNA2 | Karyopherin alpha 2 (RAG cohort 1, importin alpha 1) |
| LAD1 | Ladinin 1 |
| LAMB3 | Laminin, beta 3 |
| LARP1B | La ribonucleoprotein domain family, member 1B |
| LARP4 | La ribonucleoprotein domain family, member 4 |
| LIG4 | Ligase IV, DNA, ATP-dependent |
| LIMA1 | LIM domain and actin binding 1 |
| LIN7C | Lin-7 homolog C (C. elegans) |
| LMO4 | LIM domain only 4 |
| LTBP3 | Latent transforming growth factor beta binding protein 3 |
| LY75 | Lymphocyte antigen 75 |
| LY9 | Lymphocyte antigen 9 |
| LYPLA1 | Lysophospholipase I |
| MAF | V-maf musculoaponeurotic fibrosarcoma oncogene homolog (avian) |
| MANF | Mesencephalic astrocyte-derived neurotrophic factor |
| MATK | Megakaryocyte-associated tyrosine kinase |
| MBNL2 | Muscleblind-like splicing regulator 2 |
| ME3 | Malic enzyme 3, NADP(+)-dependent, mitochondrial |
| MED21 | Mediator complex subunit 21 |
| MEI1 | Meiosis inhibitor 1 |
| MERTK | C-mer proto-oncogene tyrosine kinase |
| METRNL | Meteorin, glial cell differentiation regulator-like |
| MFSD2A | Major facilitator superfamily domain containing 2A |
| MIS12 | MIS12, MIND kinetochore complex component, homolog (S. pombe) |
| MLEC | Malectin |
| MPLKIP | M-phase specific PLK1 interacting protein |
| MPV17L2 | MPV17 mitochondrial membrane protein-like 2 |
| MSH2 | mutS homolog 2, colon cancer, nonpolyposis type 1 (E. coli) |
| MSTO1 | Misato homolog 1 (Drosophila) |
| MT1G | Metallothionein 1G |
| MT1H | Metallothionein 1H |
| MT1M | Metallothionein 1M |
| MTFR1 | Mitochondrial fission regulator 1 |
| MTHFD2 | Methylenetetrahydrofolate dehydrogenase (NADP+ dependent) 2, methenyltetrahydrofolate cyclohydrolase |
| MTMR4 | Myotubularin related protein 4 |
| MYOM2 | Myomesin 2 |
| N4BP2 | NEDD4 binding protein 2 |
| NDUFA5 | NADH dehydrogenase (ubiquinone) 1 alpha subcomplex, 5, 13kDa |
| NFKBIA | Nuclear factor of kappa light polypeptide gene enhancer in B-cells inhibitor, alpha |
| NFXL1 | Nuclear transcription factor, X-box binding-like 1 |
| NPLOC4 | Nuclear protein localization 4 homolog (S. cerevisiae) |
| NUCB2 | Nucleobindin 2 |
| OSTC | Oligosaccharyltransferase complex subunit |
| PC | Pyruvate carboxylase |

| | |
|---|---|
| PDE4B | Phosphodiesterase 4B, cAMP-specific |
| PDIA4 | Protein disulfide isomerase family A, member 4 |
| PDIA5 | Protein disulfide isomerase family A, member 5 |
| PDIA6 | Protein disulfide isomerase family A, member 6 |
| PELI1 | Pellino E3 ubiquitin protein ligase 1 |
| PFDN2 | Prefoldin subunit 2 |
| PGM3 | Phosphoglucomutase 3 |
| PIGA | Phosphatidylinositol glycan anchor biosynthesis, class A |
| PIK3AP1 | Phosphoinositide-3-kinase adaptor protein 1 |
| PIM2 | Pim-2 oncogene |
| PLEKHJ1 | Pleckstrin homology domain containing, family J member 1 |
| PNP | Purine nucleoside phosphorylase |
| PPIG | Peptidylprolyl isomerase G (cyclophilin G) |
| PPIL1 | Peptidylprolyl isomerase (cyclophilin)-like 1 |
| PPP1CB | Protein phosphatase 1, catalytic subunit, beta isozyme |
| PPP1R15B | Protein phosphatase 1, regulatory subunit 15B |
| PPP2CB | Protein phosphatase 2, catalytic subunit, beta isozyme |
| PRKCSH | Protein kinase C substrate 80K-H |
| PRPF4B | PRP4 pre-mRNA processing factor 4 homolog B (yeast) |
| PRRC1 | Proline-rich coiled-coil 1 |
| PSAT1 | Phosphoserine aminotransferase 1 |
| PSMC6 | Proteasome (prosome, macropain) 26S subunit, ATPase, 6 |
| PSTPIP2 | Proline-serine-threonine phosphatase interacting protein 2 |
| PTGES | Prostaglandin E synthase |
| PTMA | Prothymosin, alpha |
| RABAC1 | Rab acceptor 1 (prenylated) |
| RAD9A | RAD9 homolog A (S. pombe) |
| RAP1B | RAP1B, member of RAS oncogene family |
| RCC1 | Regulator of chromosome condensation 1 |
| RGS2 | Regulator of G-protein signaling 2, 24kDa |
| RNASE4 | Ribonuclease, RNase A family, 4 |
| RNF19B | Ring finger protein 19B |
| ROCK1 | Rho-associated, coiled-coil containing protein kinase 1 |
| RPLP1 | Ribosomal protein, large, P1 |
| RPN1 | Ribophorin I |
| RTKN | Rhotekin |
| RTP4 | Receptor (chemosensory) transporter protein 4 |
| SAR1A | SAR1 homolog A (S. cerevisiae) |
| SDF2L1 | Stromal cell-derived factor 2-like 1 |
| SEC11C | SEC11 homolog C (S. cerevisiae) |
| SEC24D | SEC24 family, member D (S. cerevisiae) |
| SEC31A | SEC31 homolog A (S. cerevisiae) |
| SEL1L | Sel-1 suppressor of lin-12-like (C. elegans) |
| SELK | Selenoprotein K |
| SELT | Selenoprotein T precursor |
| SEMA3A | Sema domain, immunoglobulin domain (Ig), short basic domain, secreted, (semaphorin) 3A |
| SERPINE1 | Serpin peptidase inhibitor, clade E (nexin, plasminogen activator inhibitor type 1), member 1 |
| SERTAD3 | SERTA domain containing 3 |
| SIL1 | SIL1 homolog, endoplasmic reticulum chaperone (S. cerevisiae) |
| SLA | Src-like-adaptor |
| SLAMF1 | Signaling lymphocytic activation molecule family member 1 |
| SLC10A7 | Solute carrier family 10 (sodium/bile acid cotransporter family), member 7 |
| SLC17A9 | Solute carrier family 17, member 9 |
| SLC1A4 | Solute carrier family 1 (glutamate/neutral amino acid transporter), member 4 |
| SLC1A5 | Solute carrier family 1 (neutral amino acid transporter), member 5 |
| SLC22A16 | Solute carrier family 22 (organic cation/carnitine transporter), member 16 |
| SLC26A6 | Solute carrier family 26, member 6 |
| SLC35B1 | Solute carrier family 35, member B1 |
| SLC39A14 | Solute carrier family 39 (zinc transporter), member 14 |
| SLC39A8 | Solute carrier family 39 (zinc transporter), member 8 |
| SLC3A2 | Solute carrier family 3 (activators of dibasic and neutral amino acid transport), member 2 |
| SLC7A1 | Solute carrier family 7 (cationic amino acid transporter, y+ system), member 1 |
| SLC7A5 | Solute carrier family 7 (amino acid transporter light chain, L system), member 5 |
| SLC9A9 | Solute carrier family 9, subfamily A (NHE9, cation proton antiporter 9), member 9 |
| SLFN11 | Schlafen family member 11 |
| SP140 | SP140 nuclear body protein |
| SPCS2 | Signal peptidase complex subunit 2 homolog (S. cerevisiae) |
| SPCS3 | Signal peptidase complex subunit 3 homolog (S. cerevisiae) |

| | |
|---|---|
| SPOCD1 | SPOC domain containing 1 |
| SRSF11 | Serine/arginine-rich splicing factor 11 |
| SRSF3 | Serine/arginine-rich splicing factor 3 |
| ST6GALNAC4 | ST6 (alpha-N-acetyl-neuraminyl-2,3-beta-galactosyl-1,3)-N-acetylgalactosaminide alpha-2,6-sialyltransferase 4 |
| SUMO1 | SMT3 suppressor of mif two 3 homolog 1 (S. cerevisiae) |
| SYVN1 | Synovial apoptosis inhibitor 1, synoviolin |
| TAF9 | TAF9 RNA polymerase II, TATA box binding protein (TBP)-associated factor, 32kDa |
| TANK | TRAF family member-associated NFKB activator |
| TARS | threonyl-tRNA synthetase |
| TCP1 | T-complex 1 |
| TDP2 | tyrosyl-DNA phosphodiesterase 2 |
| TES | Testis derived transcript (3 LIM domains) |
| TLR1 | Toll-like receptor 1 |
| TMED7 | Transmembrane emp24 protein transport domain containing 7 |
| TMEM163 | Transmembrane protein 163 |
| TMEM38B | Transmembrane protein 38B |
| TMEM39A | Transmembrane protein 39A |
| TMEM41B | Transmembrane protein 41B |
| TMEM50B | Transmembrane protein 50B |
| TMEM91 | Transmembrane protein 91 |
| TNFRSF4 | Tumor necrosis factor receptor superfamily, member 4 |
| TNFRSF9 | Tumor necrosis factor receptor superfamily, member 9 |
| TNFSF10 | Tumor necrosis factor (ligand) superfamily, member 10 |
| TOB1 | Transducer of ERBB2, 1 |
| TOPORS | Topoisomerase I binding, arginine/serine-rich, E3 ubiquitin protein ligase |
| TRAF1 | TNF receptor-associated factor 1 |
| TRAM2 | Translocation associated membrane protein 2 |
| TRIB1 | Tribbles homolog 1 (Drosophila) |
| TRIB3 | Tribbles homolog 3 (Drosophila) |
| TRIM47 | Tripartite motif containing 47 |
| TRIM5 | Tripartite motif containing 5 |
| TSN | Translin |
| TUBA3D | Tubulin, alpha 3d |
| TUBB3 | Tubulin, beta 3 class III |
| UAP1 | UDP-N-acteylglucosamine pyrophosphorylase 1 |
| UBA5 | Ubiquitin-like modifier activating enzyme 5 |
| UBE2T | Ubiquitin-conjugating enzyme E2T (putative) |
| UBXN1 | UBX domain protein 1 |
| UFD1L | Ubiquitin fusion degradation 1 like (yeast) |
| UGDH | UDP-glucose 6-dehydrogenase |
| UPB1 | Ureidopropionase, beta |
| UQCRH | Ubiquinol-cytochrome c reductase hinge protein |
| USP18 | Ubiquitin specific peptidase 18 |
| VPS26A | Vacuolar protein sorting 26 homolog A (S. pombe) |
| VSX2 | Visual system homeobox 2 |
| VWF | Von Willebrand factor |
| WFS1 | Wolfram syndrome 1 (wolframin) |
| WIPI1 | WD repeat domain, phosphoinositide interacting 1 |
| WTAP | Wilms tumor 1 associated protein |
| XCR1 | Chemokine (C motif) receptor 1 |
| ZC3H12A | Zinc finger CCCH-type containing 12A |
| ZFAND2A | Zinc finger, AN1-type domain 2A |
| ZNF395 | Zinc finger protein 395 |
| ZNF768 | Zinc finger protein 768 |
