# Supplementary material for: Pathogen‐induced inflammation is attenuated by the iminosugar MON‐DNJ via modulation of the unfolded protein response
Source: Immunology. 2021 Aug 1;164(3):587–601. doi: 10.1111/imm.13393 (PMC8517592; doi:10.1111/imm.13393)
Supplement: Supplementary file 6 — Table S3 [file IMM-164-587-s005.pdf]

Supplemental Table 3A. STRING-DB network analysis for biological process enrichment of Cluster 1 transcripts

Pathway	Count	FDR	Targets
response to endoplasmic reticulum stress	15	8.67E-17	AARS,ASNS,DERL2,DNAJB11,DNAJB9,HERPUD1,HSP90B1,HSPA5,HYOU1,PDIA4,PDIA6,SDF2L1,SELK,SYVN1,TRIB3
response to topologically incorrect protein	14	1.30E-16	AARS,ASNS,DDIT3,DERL2,DNAJB11,DNAJB9,HERPUD1,HSP90B1,HSPA5,HYOU1,MANF,PDIA6,SDF2L1,SYVN1
cellular response to topologically incorrect protein	13	1.30E-16	AARS,ASNS,DDIT3,DERL2,DNAJB11,DNAJB9,HERPUD1,HSP90B1,HSPA5,HYOU1,PDIA6,SDF2L1,SYVN1
response to unfolded protein	13	1.89E-15	AARS,ASNS,DDIT3,DERL2,DNAJB11,DNAJB9,HERPUD1,HSP90B1,HSPA5,HYOU1,MANF,PDIA6,SYVN1
endoplasmic reticulum unfolded protein response	12	1.89E-15	AARS,ASNS,DDIT3,DERL2,DNAJB11,DNAJB9,HERPUD1,HSP90B1,HSPA5,HYOU1,PDIA6,SYVN1
cellular response to unfolded protein	12	2.24E-15	AARS,ASNS,DDIT3,DERL2,DNAJB11,DNAJB9,HERPUD1,HSP90B1,HSPA5,HYOU1,PDIA6,SYVN1
ERAD pathway	8	4.37E-10	DERL2,DNAJB9,HERPUD1,HSP90B1,HSPA5,SDF2L1,SEL1L,SYVN1
ER-associated ubiquitin-dependent protein catabolic process	7	2.20E-08	DERL2,DNAJB9,HSP90B1,HSPA5,SDF2L1,SEL1L,SYVN1
retrograde protein transport, ER to cytosol	5	8.76E-08	DERL2,HERPUD1,HSP90B1,SEL1L,SYVN1
IRE1-mediated unfolded protein response	6	1.39E-06	DNAJB11,DNAJB9,HSPA5,HYOU1,PDIA6,SYVN1
cellular response to stress	16	4.51E-06	AARS,ASNS,DDIT3,DERL2,DNAJB11,DNAJB9,HERPUD1,HSP90B1,HSPA5,HYOU1,PDIA4,PDIA6,SDF2L1,SELK,SYVN1,TRIB3
PERK-mediated unfolded protein response	4	5.44E-06	ASNS,DDIT3,HERPUD1,HSPA5
ubiquitin-dependent protein catabolic process	9	5.66E-05	DDIT3,DERL2,DNAJB9,HERPUD1,HSP90B1,HSPA5,SDF2L1,SEL1L,SYVN1
proteasome-mediated ubiquitin-dependent protein catabolic process	8	5.66E-05	DDIT3,DERL2,DNAJB9,HSP90B1,HSPA5,SDF2L1,SEL1L,SYVN1
response to stress	20	8.97E-05	AARS,ANG,ASNS,DDIT3,DERL2,DNAJB11,DNAJB9,HERPUD1,HSP90B1,HSPA5,HYOU1,MANF,PDIA4,PDIA6,SDF2L1,SELK,SLC7A5,SLFN11,SYVN1,TRIB3
regulation of endoplasmic reticulum stress-induced intrinsic apoptotic signaling pathway	4	0.00012	DDIT3,HERPUD1,HYOU1,SYVN1
cellular protein catabolic process	9	0.000122	DDIT3,DERL2,DNAJB9,HERPUD1,HSP90B1,HSPA5,SDF2L1,SEL1L,SYVN1
negative regulation of response to endoplasmic reticulum stress	4	0.000171	DERL2,HERPUD1,HYOU1,SYVN1
ATF6-mediated unfolded protein response	3	0.000294	DDIT3,HSP90B1,HSPA5
response to organic substance	16	0.000654	AARS,ANG,DDIT3,DERL2,DNAJB11,DNAJB9,HERPUD1,HSP90B1,HSPA5,HYOU1,MANF,PDIA6,SDF2L1,SEL1L,SYVN1,TRIB3
protein folding	6	0.000973	AARS,DNAJB11,HSP90B1,HSPA5,PDIA4,PDIA6
negative regulation of endoplasmic reticulum stress-induced intrinsic apoptotic signaling pathway	3	0.00174	HERPUD1,HYOU1,SYVN1
cell redox homeostasis	4	0.00267	DDIT3,ERO1LB,PDIA4,PDIA6
cellular response to organic substance	13	0.00349	AARS,DDIT3,DERL2,DNAJB11,DNAJB9,HERPUD1,HSP90B1,HSPA5,HYOU1,PDIA6,SDF2L1,SYVN1,TRIB3
proteolysis	10	0.00555	DDIT3,DERL2,DNAJB9,ERO1LB,HERPUD1,HSP90B1,HSPA5,SDF2L1,SEL1L,SYVN1
ER-nucleus signaling pathway	3	0.0113	ASNS,HERPUD1,HSP90B1
intrinsic apoptotic signaling pathway in response to endoplasmic reticulum stress	3	0.012	DDIT3,SELK,TRIB3
protein folding in endoplasmic reticulum	2	0.0367	HSP90B1,HSPA5

Supplemental Table 3B. STRING-DB network analysis for biological process enrichment of Cluster 2 transcripts

Pathway	Count	FDR	Targets
response to endoplasmic reticulum stress	14	1.39E-09	ASNS,ATF4,ATP2A2,DNAJC10,EIF2AK3,FAM129A,FKBP14,GFPT1,PDIA5,SEC31A,SYVN1,UBA5,WFS1,WIPI1
endoplasmic reticulum unfolded protein response	10	1.58E-07	ASNS,ATF4,EIF2AK3,FKBP14,GFPT1,PDIA5,SEC31A,SYVN1,WFS1,WIPI1
cellular response to unfolded protein	10	1.58E-07	ASNS,ATF4,EIF2AK3,FKBP14,GFPT1,PDIA5,SEC31A,SYVN1,WFS1,WIPI1
protein N-linked glycosylation via asparagine	10	2.87E-07	GFPT1,MLEC,OSTC,PGM3,RPN1,SEC24D,SEC31A,ST6GALNAC4,SYVN1,UAP1
IRE1-mediated unfolded protein response	7	6.85E-06	FKBP14,GFPT1,PDIA5,SEC31A,SYVN1,WFS1,WIPI1
cellular response to stress	22	0.000222	ASNS,ATF4,ATP2A2,CISD2,DDIT4,DNAJC10,EIF2AK3,FAM129A,FKBP14,GFPT1,LARP1B,MSH2,PDIA5,RAD9A,SEC31A,SLC1A4,SYVN1,TMEM39A,UBA5,UBE2T,WFS1,WIPI1
nucleotide-sugar biosynthetic process	4	0.000491	GFPT1,PGM3,UAP1,UGDH
single-organism carbohydrate metabolic process	13	0.000538	ATF4,GFPT1,GOT1,MLEC,OSTC,PGM3,RPN1,SEC24D,SEC31A,ST6GALNAC4,SYVN1,UAP1,UGDH
UDP-N-acetylglucosamine biosynthetic process	3	0.000599	GFPT1,PGM3,UAP1
glycoprotein biosynthetic process	10	0.00125	GFPT1,MLEC,OSTC,PGM3,RPN1,SEC24D,SEC31A,ST6GALNAC4,SYVN1,UAP1
protein folding	8	0.00147	DNAJC10,FKBP11,FKBP14,MLEC,PDIA5,PPIL1,TUBB3,WFS1
carbohydrate metabolic process	14	0.00158	ATF4,GFPT1,GOT1,MLEC,OSTC,PGM3,RPN1,SEC24D,SEC31A,SLC3A2,ST6GALNAC4,SYVN1,UAP1,UGDH
signal peptide processing	3	0.00257	SEC11C,SPCS2,SPCS3
carbohydrate derivative biosynthetic process	12	0.00318	GFPT1,MLEC,OSTC,PGM3,PIGA,RPN1,SEC24D,SEC31A,ST6GALNAC4,SYVN1,UAP1,UGDH
peptidyl-amino acid modification	14	0.00354	EIF2AK3,FKBP11,FKBP14,GFPT1,MLEC,OSTC,PGM3,PPIL1,RPN1,SEC24D,SEC31A,ST6GALNAC4,SYVN1,UAP1
ER-nucleus signaling pathway	4	0.00416	ASNS,ATF4,ATP2A2,WFS1
amide biosynthetic process	10	0.00416	ASNS,C12orf65,CARS,EIF2AK3,MTHFD2,RPN1,SEC11C,SPCS2,SPCS3,TARS
post-translational protein modification	9	0.00416	GFPT1,MLEC,PGM3,PIGA,RPN1,SEC24D,SEC31A,ST6GALNAC4,UAP1
cellular protein metabolic process	29	0.00583	APP,ASNS,ATF4,C12orf65,CARS,DNAJC10,FKBP11,FKBP14,GFPT1,MLEC,MTMR4,OSTC,PDIA5,PGM3,PIGA,PPIL1,RPN1,SEC24D,SEC31A,ST6GALNAC4,SYVN1,TARS,TRIM5,TUBB3,UAP1,UBA5,UBE2T,WFS1,WIPI1
PERK-mediated unfolded protein response	3	0.00685	ASNS,ATF4,EIF2AK3
single-organism metabolic process	32	0.0155	ALDH1L2,ANG,APP,ASNS,ATF4,CARS,CISD2,DNAJC10,EIF2AK3,GFPT1,GPT2,MLEC,MSH2,MTFR1,MTHFD2,MTMR4,OSTC,PDIA5,PGM3,PIGA,RAD9A,RPN1,SEC24D,SEC31A,ST6GALNAC4,SYVN1,TARS,UAP1,UBE2T,UGDH,WFS1,WIPI1
glutamine transport	2	0.0217	SLC1A4,SLC1A5
endoplasmic reticulum calcium ion homeostasis	3	0.0228	APP,ATP2A2,WFS1
translation	8	0.0274	C12orf65,CARS,EIF2AK3,RPN1,SEC11C,SPCS2,SPCS3,TARS
response to stress	27	0.0464	ANG,ASNS,ATF4,ATP2A2,CISD2,DDIT4,DNAJC10,EIF2AK3,FAM129A,FKBP14,GFPT1,HABP4,LARP1B,MSH2,PDIA5,RAD9A,SEC31A,SLC1A4,SLC3A2,SP140,SYVN1,TMEM39A,TRIM5,UBA5,UBE2T,WFS1,WIPI1

Supplemental Table 3C. STRING-DB network analysis for biological process enrichment of Cluster 6 transcripts

Pathway	Count	FDR	Targets
response to lipopolysaccharide	10	6.73E-06	ANKRD1,CYP27B1,IRAK2,NFKBIA,PDE4B,PELI1,PTGES,SERPINE1,TRIB1,ZC3H12A
positive regulation of B cell proliferation	5	7.14E-05	ADA,CD38,GPR183,PELI1,TNFRSF4
response to other organism	12	7.14E-05	ANKRD1,BATF,CYP27B1,IER3,IRAK2,NFKBIA,PDE4B,PELI1,PTGES,SERPINE1,TRIB1,ZC3H12A
regulation of cell activation	10	8.10E-05	ADA,C1QTNF1,CD38,EBI3,GPR183,HSPH1,MERTK,PELI1,TNFRSF4,ZC3H12A
response to bacterium	10	0.000127	ANKRD1,CYP27B1,IRAK2,NFKBIA,PDE4B,PELI1,PTGES,SERPINE1,TRIB1,ZC3H12A
regulation of leukocyte activation	9	0.000275	ADA,CD38,EBI3,GPR183,HSPH1,MERTK,PELI1,TNFRSF4,ZC3H12A
positive regulation of lymphocyte proliferation	6	0.000275	ADA,CD38,EBI3,GPR183,PELI1,TNFRSF4
response to external stimulus	17	0.000287	ADA,ANKRD1,BATF,CCL23,CCL4L2,CYP27B1,FPR1,FPR3,IER3,IRAK2,NFKBIA,PDE4B,PELI1,PTGES,SERPINE1,TRIB1,ZC3H12A
cellular response to lipopolysaccharide	6	0.000484	ANKRD1,IRAK2,NFKBIA,PDE4B,SERPINE1,ZC3H12A
positive regulation of lymphocyte activation	7	0.000594	ADA,CD38,EBI3,GPR183,HSPH1,PELI1,TNFRSF4
negative regulation of sequence-specific DNA binding transcription factor activity	6	0.000643	IRAK2,NFKBIA,PELI1,TNFRSF4,TRIB1,ZC3H12A
regulation of lymphocyte activation	8	0.000655	ADA,CD38,EBI3,GPR183,HSPH1,MERTK,PELI1,TNFRSF4
response to lipid	11	0.000753	ANKRD1,CD38,CYP27B1,IRAK2,NFKBIA,PDE4B,PELI1,PTGES,SERPINE1,TRIB1,ZC3H12A
regulation of cytokine production	9	0.0013	ALOX15B,EBI3,NFKBIA,PDE4B,PELI1,SERPINE1,TNFRSF4,TNFRSF9,ZC3H12A
regulation of cell proliferation	14	0.00138	ADA,ALOX15B,CCL23,CD38,CYP27B1,EBI3,GPR183,NFKBIA,PELI1,PTGES,SLAMF1,TNFRSF4,TNFRSF9,TRIB1
regulation of localization	17	0.0019	ADA,ALOX15B,ANKRD1,C1QTNF1,CD38,CLIC2,HSPH1,IER3,MERTK,NFKBIA,PDE4B,SERPINE1,SLAMF1,TNFRSF4,TNFRSF9,TRIB1,ZC3H12A
cellular response to zinc ion	3	0.00324	MT1G,MT1H,MT1M
negative regulation of biological process	23	0.00336	ALOX15B,ANKRD1,C1QTNF1,CCL23,CLIC2,CYP27B1,DNAJA4,GPR132,HSPH1,IRAK2,LIMA1,MERTK,MT1G,MT1H,MT1M,NFKBIA,PDE4B,PELI1,PTGES,SERPINE1,TNFRSF4,TRIB1,ZC3H12A
negative regulation of multicellular organismal process	11	0.00336	ADA,C1QTNF1,CD38,FKBP4,NFKBIA,PDE4B,SERPINE1,TNFRSF4,TNFRSF9,TRIB1,ZC3H12A
defense response	13	0.00353	BATF,CCL23,CCL4L2,CD163,CHST2,CYP27B1,IRAK2,NFKBIA,PELI1,PTGES,RNF19B,SERPINE1,TNFRSF4
positive regulation of immune system process	10	0.00477	ADA,CD38,EBI3,GPR183,HSPH1,IRAK2,PDE4B,SERPINE1,TNFRSF4,TRIB1
multi-organism process	16	0.00503	ANKRD1,BATF,CD38,CYP27B1,FKBP4,IER3,IRAK2,MERTK,NFKBIA,PDE4B,PELI1,PTGES,SERPINE1,SLAMF1,TRIB1,ZC3H12A
response to stress	20	0.00588	ANKRD1,BATF,CCL23,CCL4L2,CD163,CD38,CHST2,CYP27B1,DNAJA4,FKBP4,HSPH1,IER3,IRAK2,NFKBIA,PELI1,PTGES,RNF19B,SERPINE1,TNFRSF4,TRIB1
regulation of transport	14	0.00588	ADA,ALOX15B,ANKRD1,C1QTNF1,CD38,CLIC2,IER3,MERTK,NFKBIA,PDE4B,SERPINE1,SLAMF1,TNFRSF4,TNFRSF9
negative regulation of NF-kappaB transcription factor activity	4	0.00642	IRAK2,NFKBIA,PELI1,ZC3H12A
regulation of sequence-specific DNA binding transcription factor activity	7	0.00642	IRAK2,NFKBIA,PELI1,TNFRSF4,TRAF1,TRIB1,ZC3H12A
cellular response to stimulus	27	0.00642	ANKRD1,BATF,CCL23,CCL4L2,CD38,CLIC2,CYP27B1,EBI3,FPR1,FPR3,GPR132,GPR183,HSPH1,IER3,IRAK2,MT1G,MT1H,MT1M,NFKBIA,PDE4B,PTGES,SERPINE1,SLAMF1,TNFRSF4,TRAF1,TRIB1,ZC3H12A
immune response	12	0.00784	ADA,BATF,CCL23,CCL4L2,CYP27B1,EBI3,GPR183,IRAK2,NFKBIA,PELI1,RNF19B,TNFRSF4
response to oxygen-containing compound	12	0.00839	ADA,ANKRD1,CD38,CYP27B1,IRAK2,NFKBIA,PDE4B,PELI1,PTGES,SERPINE1,TRIB1,ZC3H12A
leukocyte activation	7	0.00922	BATF,EBI3,GPR183,MERTK,MT1G,SLAMF1,TNFRSF4
regulation of immune system process	12	0.00981	ADA,CD38,EBI3,GPR183,HSPH1,IRAK2,MERTK,PDE4B,SERPINE1,TNFRSF4,TRIB1,ZC3H12A
inflammatory response	7	0.015	CCL23,CCL4L2,CD163,CHST2,IRAK2,PTGES,TNFRSF4
B cell activation involved in immune response	3	0.0166	ADA,BATF,GPR183
response to chemical	20	0.0166	ADA,ANKRD1,CCL23,CCL4L2,CYP27B1,EBI3,FPR1,FPR3,HSPH1,IRAK2,MT1G,MT1H,MT1M,PDE4B,PELI1,PTGES,SERPINE1,TNFRSF4,TRIB1,ZC3H12A
cell activation	8	0.0219	BATF,EBI3,GPR183,MERTK,MT1G,SERPINE1,SLAMF1,TNFRSF4
lymphocyte activation	6	0.0263	BATF,EBI3,GPR183,MERTK,SLAMF1,TNFRSF4
response to stimulus	28	0.0263	ANKRD1,BATF,CCL23,CCL4L2,CD163,CHST2,CLIC2,CYP27B1,DNAJA4,EBI3,FPR1,FPR3,GPR132,GPR183,HSPH1,IER3,IRAK2,MT1G,MT1H,MT1M,NFKBIA,PDE4B,RNF19B,SERPINE1,SLAMF1,TRAF1,TRIB1,ZC3H12A
regulation of multicellular organismal process	15	0.03	ADA,ANKRD1,C1QTNF1,CD38,CLIC2,CYP27B1,EBI3,FKBP4,PDE4B,PELI1,SERPINE1,TNFRSF4,TNFRSF9,TRIB1,ZC3H12A
negative regulation of molecular function	10	0.0331	CCL23,CLIC2,CYP27B1,IRAK2,NFKBIA,PELI1,SERPINE1,TNFRSF4,TRIB1,ZC3H12A
regulation of molecular function	16	0.0347	ARHGEF10L,CCL23,CLIC2,CYP27B1,FPR1,HSPH1,IRAK2,NFKBIA,PDE4B,PELI1,SERPINE1,SLAMF1,TNFRSF4,TRAF1,TRIB1,ZC3H12A
positive regulation of multicellular organismal process	11	0.0377	ADA,ANKRD1,C1QTNF1,CD38,CYP27B1,EBI3,NFKBIA,PDE4B,PELI1,SERPINE1,TRIB1
positive regulation of cytokine production	6	0.0393	ALOX15B,EBI3,NFKBIA,PDE4B,PELI1,SERPINE1
negative regulation of growth	5	0.0393	ALOX15B,CYP27B1,MT1G,MT1H,MT1M
negative regulation of immune system process	6	0.041	ADA,MERTK,NFKBIA,PELI1,TRIB1,ZC3H12A
positive regulation of alpha-beta T cell activation	3	0.041	ADA,EBI3,HSPH1
response to organic substance	15	0.0423	ADA,ANKRD1,CD38,CYP27B1,EBI3,HSPH1,IRAK2,MT1G,PDE4B,PELI1,PTGES,SERPINE1,TNFRSF4,TRIB1,ZC3H12A
mature B cell differentiation involved in immune response	2	0.0449	ADA,GPR183

Supplemental Table 3D. STRING-DB network analysis of differentially expressed transcripts

Category	Pathway	Count	FDR	Targets
1	response to endoplasmic reticulum stress	35	1.43E-22	AARS,ASNA1,ASNS,ATF3,ATF4,ATP2A2,CALR,DERL2,DNAJB11,DNAJB9,DNAJC10,EIF2AK3,FAM129A,FKBP14,GFPT1,HERPUD1,HSP90B1,HSPA5,HYOU1,NPLOC4,PDIA4,PDIA5,PDIA6,PPP1R15B,PPP2CB,PSMC6,SDF2L1,SEC31A,SELK,SYVN1,TRIB3,UBA5,UFD1L,WFS1,WIPI1
1	response to topologically incorrect protein	29	7.93E-19	AARS,ASNA1,ASNS,ATF3,ATF4,ATXN3,CALR,DDIT3,DERL2,DNAJB11,DNAJB5,DNAJB9,EIF2AK3,FKBP14,GFPT1,HERPUD1,HSP90B1,HSPA5,HSPH1,HYOU1,MANF,PDIA5,PDIA6,SDF2L1,SEC31A,SYVN1,UFD1L,WFS1,WIPI1
1	cellular response to topologically incorrect protein	26	7.93E-19	AARS,ASNA1,ASNS,ATF3,ATF4,ATXN3,CALR,DDIT3,DERL2,DNAJB11,DNAJB9,EIF2AK3,FKBP14,GFPT1,HERPUD1,HSP90B1,HSPA5,HYOU1,PDIA5,PDIA6,SDF2L1,SEC31A,SYVN1,UFD1L,WFS1,WIPI1
1	response to unfolded protein	26	2.12E-16	AARS,ASNA1,ASNS,ATF3,ATF4,CALR,DDIT3,DERL2,DNAJB11,DNAJB5,DNAJB9,EIF2AK3,FKBP14,GFPT1,HERPUD1,HSP90B1,HSPA5,HSPH1,HYOU1,MANF,PDIA5,PDIA6,SEC31A,SYVN1,WFS1,WIPI1
1	endoplasmic reticulum unfolded protein response	23	2.12E-16	AARS,ASNA1,ASNS,ATF3,ATF4,CALR,DDIT3,DERL2,DNAJB11,DNAJB9,EIF2AK3,FKBP14,GFPT1,HERPUD1,HSP90B1,HSPA5,HYOU1,PDIA5,PDIA6,SEC31A,SYVN1,WFS1,WIPI1
1	cellular response to unfolded protein	23	2.78E-16	AARS,ASNA1,ASNS,ATF3,ATF4,CALR,DDIT3,DERL2,DNAJB11,DNAJB9,EIF2AK3,FKBP14,GFPT1,HERPUD1,HSP90B1,HSPA5,HYOU1,PDIA5,PDIA6,SEC31A,SYVN1,WFS1,WIPI1
1	protein folding	27	2.12E-14	AARS,CALR,DNAJA4,DNAJB11,DNAJB5,DNAJC10,FKBP11,FKBP14,FKBP2,FKBP4,FKBP5,HSP90B1,HSPA5,HSPH1,MLEC,PDIA4,PDIA5,PDIA6,PFDN2,PPIG,PPIL1,PRKCSH,SIL1,TCP1,TUBA3D,TUBB3,WFS1
1	cellular response to stress	71	4.02E-14	AARS,ANKRD1,ASNA1,ASNS,ATF4,ATMIN,ATP1B1,ATP2A2,ATXN3,BATF,BCL2A1,CALR,CD36,CISD2,DDIT3,DDIT4,DERL2,DNAJB11,DNAJB9,DNAJC10,DTX3L,DUSP6,DYRK2,EEPD1,EIF2AK3,FAM129A,FANCE,FKBP14,FKBP4,GABARAPL2,GFPT1,HERPUD1,HMGB1,HSP90B1,HSPA5,HSPH1,HTRA2,HYOU1,ID2,IER3,IRAK2,KLF4,LARP1B,LIG4,MSH2,NFKBIA,NPLOC4,PDIA4,PDIA5,PDIA6,PPP1CB,PPP1R15B,PPP2CB,PSMC6,RAD9A,SDF2L1,SEC31A,SELK,SLC1A4,SUMO1,SYVN1,TAF9,TDP2,TMEM39A,TRIB1,TRIB3,UBA5,UBE2T,UFD1L,WFS1,WIPI1
1	response to stress	105	4.00E-12	AARS,ABAT,ACP5,ANG,ANKRD1,ASNA1,ASNS,ATF4,ATMIN,ATP2A2,ATXN3,BATF,BCL2A1,CALR,CAPZA1,CCL23,CCL4L2,CD163,CD38,CHST2,CISD2,CTNNB1,CYP27B1,DDIT3,DDIT4,DERL2,DNAJA4,DNAJB11,DNAJB5,DNAJB9,DNAJC10,DTX3L,DUSP6,DYRK2,EEPD1,EIF2AK3,FABP5,FAM129A,FANCE,FKBP14,FKBP4,GABARAPL2,GFPT1,HABP4,HERPUD1,HMGB1,HSP90B1,HSPA5,HSPH1,HYOU1,ID2,IER3,IRAK2,KLF4,LARP1B,LIG4,LY75,MANF,MATK,METRNL,MSH2,NFKBIA,NPLOC4,PDIA4,PDIA5,PDIA6,PELI1,PPP1CB,PPP1R15B,PPP2CB,PRKCSH,PSMC6,PTGES,RAD9A,RAP1B,RNF19B,SDF2L1,SEC31A,SELK,SERPINE1,SLC1A4,SLC26A6,SLC3A2,SLC7A5,SLFN11,SP140,SUMO1,SYVN1,TAF9,TANK,TDP2,TLR1,TMEM39A,TNFRSF4,TRIB1,TRIB3,TRIM5,UBA5,UBE2T,UFD1L,USP18,VWF,WFS1,WIPI1,XCR1
1	IRE1-mediated unfolded protein response	13	7.12E-09	ASNA1,DNAJB11,DNAJB9,FKBP14,GFPT1,HSPA5,HYOU1,PDIA5,PDIA6,SEC31A,SYVN1,WFS1,WIPI1
1	ERAD pathway	13	1.29E-08	DERL2,DNAJB9,DNAJC10,HERPUD1,HSP90B1,HSPA5,NPLOC4,PSMC6,SDF2L1,SEL1L,SYVN1,UFD1L,WFS1
4	response to organic substance	76	7.84E-08	AARS,ACP5,ADA,ANG,ANKRD1,ASNA1,ATF3,ATF4,ATP2A2,ATXN3,CD36,CD38,CTNNB1,CTR9,CYP27B1,DDIT3,DDIT4,DERL2,DNAJB11,DNAJB5,DNAJB9,DNAJC10,EBI3,EIF2AK3,FKBP14,GCLC,GDF15,GFPT1,GNB4,GNG10,GOT1,HERPUD1,HRSP12,HSP90B1,HSPA5,HSPH1,HTRA2,HYOU1,IRAK2,KLF4,KPNA2,MANF,MT1G,MTMR4,NPLOC4,PDE4B,PDIA5,PDIA6,PELI1,PPP1CB,PPP2CB,PSMC6,PTGES,RAP1B,SDF2L1,SEC31A,SEL1L,SELT,SERPINE1,SLC26A6,SLC3A2,SRSF3,SUMO1,SYVN1,TAF9,TLR1,TNFRSF4,TRIB1,TRIB3,TRIM5,UFD1L,USP18,WFS1,WIPI1,XCR1,ZC3H12A
1	ER-associated ubiquitin-dependent protein catabolic process	12	8.06E-08	DERL2,DNAJB9,DNAJC10,HSP90B1,HSPA5,NPLOC4,PSMC6,SDF2L1,SEL1L,SYVN1,UFD1L,WFS1
1	PERK-mediated unfolded protein response	7	1.71E-07	ASNS,ATF3,ATF4,DDIT3,EIF2AK3,HERPUD1,HSPA5
1	ER-nucleus signaling pathway	9	7.66E-07	ASNS,ATF3,ATF4,ATP2A2,CALR,HERPUD1,HSP90B1,PPP1R15B,WFS1
1	retrograde protein transport, ER to cytosol	7	1.10E-06	DERL2,HERPUD1,HSP90B1,NPLOC4,SEL1L,SYVN1,UFD1L
1	cellular protein metabolic process	90	1.10E-06	ALG13,ALK,APP,ASNA1,ASNS,ATF3,ATF4,ATXN3,B3GNT5,C12orf65,CALR,CARS,CTR9,DDIT3,DERL2,DNAJA4,DNAJB5,DNAJB9,DNAJC10,DTX3L,DUSP6,DYRK2,EEF1B2,EXT1,FICD,FKBP11,FKBP14,FKBP2,FKBP4,FKBP5,GADD45GIP1,GFPT1,GMPPB,HSP90B1,HSPA5,HSPH1,HTRA2,HYOU1,IRAK2,LYPLA1,MATK,MED21,MERTK,MLEC,MTMR4,NPLOC4,OSTC,PDIA4,PDIA5,PDIA6,PELI1,PFDN2,PGM3,PIGA,PIM2,PPIG,PPIL1,PPP1CB,PRPF4B,PSMC6,RNF19B,ROCK1,RPLP1,RPN1,SDF2L1,SEC24D,SEC31A,SEL1L,SELT,SIL1,ST6GALNAC4,SUMO1,SYVN1,TAF9,TARS,TCP1,TOPORS,TRIB1,TRIB3,TRIM5,TUBA3D,TUBB3,UAP1,UBA5,UBE2T,UBXN1,UFD1L,USP18,WFS1,WIPI1
4	cellular response to chemical stimulus	68	3.22E-06	AARS,ANKRD1,ASNA1,ATF3,ATF4,ATXN3,CCL23,CCL4L2,CD36,CTNNB1,CTR9,CYP27B1,CYP51A1,DDIT3,DDIT4,DERL2,DNAJB11,DNAJB9,DUSP6,EBI3,EIF2AK3,FKBP14,GCLC,GDF15,GFPT1,GNB4,GNG10,GOT1,HERPUD1,HMGB1,HSP90B1,HSPA5,HTRA2,HYOU1,ID2,IRAK2,KLF4,KPNA2,LIG4,MT1G,MT1H,MT1M,MTMR4,PDE4B,PDIA5,PDIA6,PPP1CB,PPP2CB,RAP1B,SDF2L1,SEC31A,SELT,SERPINE1,SLC26A6,SRSF3,SUMO1,SYVN1,TNFRSF4,TRIB3,TRIM5,UFD1L,UGDH,USP18,WFS1,WIPI1,XCR1,ZC3H12A,ZFAND2A
1	protein N-linked glycosylation via asparagine	14	6.66E-06	ALG13,CALR,GFPT1,GMPPB,MLEC,OSTC,PGM3,PRKCSH,RPN1,SEC24D,SEC31A,ST6GALNAC4,SYVN1,UAP1
4	response to chemical	93	9.98E-06	AARS,ACP5,ADA,ANG,ANKRD1,APP,ASNA1,ATF3,ATF4,ATP2A2,ATXN3,CCL23,CCL4L2,CD36,CTNNB1,CTR9,CYP27B1,CYP51A1,DDIT3,DDIT4,DERL2,DNAJB11,DNAJB5,DNAJB9,DNAJC10,EBI3,EIF2AK3,EVL,EXT1,FKBP14,FPR1,FPR3,GDF15,GFPT1,GNB4,GNG10,GOT1,HERPUD1,HMGB1,HRSP12,HSP90B1,HSPA5,HSPH1,HYOU1,ID2,IRAK2,KLF4,KPNA2,LIG4,MANF,MT1G,MT1H,MT1M,MTMR4,NPLOC4,PDE4B,PDIA5,PDIA6,PELI1,PNP,PPP1CB,PPP1R15B,PSMC6,PTGES,RAP1B,ROCK1,RTP4,SDF2L1,SEC31A,SEL1L,SELT,SEMA3A,SERPINE1,SLC26A6,SLC3A2,SRSF3,SUMO1,SYVN1,TAF9,TLR1,TNFRSF4,TRIB1,TRIB3,TRIM5,TUBB3,UFD1L,UGDH,USP18,WFS1,WIPI1,XCR1,ZC3H12A,ZFAND2A
4	cellular response to organic substance	57	2.02E-05	AARS,ANKRD1,ASNA1,ATF3,ATF4,ATXN3,CALR,CD36,CTNNB1,CTR9,DDIT3,DDIT4,DERL2,DNAJB11,DNAJB9,DUSP6,EBI3,EIF2AK3,FKBP14,GDF15,GFPT1,GNB4,GNG10,GOT1,HERPUD1,HSP90B1,HSPA5,HTRA2,HYOU1,IRAK2,KLF4,KPNA2,MT1G,MTMR4,PDE4B,PDIA5,PDIA6,PPP1CB,PPP2CB,RAP1B,SDF2L1,SEC31A,SELT,SERPINE1,SLC26A6,SRSF3,SUMO1,SYVN1,TNFRSF4,TRIB3,TRIM5,UFD1L,USP18,WFS1,WIPI1,XCR1,ZC3H12A
4	cellular response to biotic stimulus	14	5.23E-05	ANKRD1,CD36,CTR9,DDIT3,EIF2AK3,HSPA5,IRAK2,NFKBIA,PDE4B,PPP1R15B,SERPINE1,TLR1,WFS1,ZC3H12A
1	protein N-linked glycosylation	13	8.95E-05	ALG13,CALR,GFPT1,GMPPB,MLEC,OSTC,PGM3,RPN1,SEC24D,SEC31A,ST6GALNAC4,SYVN1,UAP1
1	ER overload response	5	0.000117	DDIT3,EIF2AK3,HSPA5,PPP1R15B,WFS1

4	response to stimulus	139	0.000154	AARS,ACP5,ALK,ANG,ANKRD1,ANP32A,ASNA1,ATF4,ATMIN,ATP1B1,ATP2A2,ATXN3,BATF,BCL2A1,CALR,CAPZA1,CCL23,CCL4L2,CD163,CD164,CD276,CD36,CDC42EP3,CHST2,CISD2,CLIC2,CTNNB1,CTNS,CTR9,CYP27B1,CYP51A1,DDIT3,DDIT4,DERL2,DNAJA4,DNAJB11,DNAJB5,DNAJB9,DNAJC10,DTX3L,DYRK2,EBI3,EEPD1,EIF2AK3,EVL,FABP5,FAM129A,FANCE,FKBP14,FPR1,FPR3,G3BP1,GABARAPL2,GABRE,GDF15,GFPT1,GNB4,GOT1,GPR132,GPR183,HABP4,HERPUD1,HMGB1,HRSP12,HSP90B1,HSPA5,HSPH1,HYOU1,IER3,IRAK2,KLF4,KPNA2,LARP1B,LIG4,LY75,MANF,MATK,METRNL,MIS12,MSH2,MT1G,MT1H,MT1M,NFKBIA,NPLOC4,PDE4B,PDIA4,PDIA5,PDIA6,PIK3AP1,PIM2,PNP,PPP1CB,PPP1R15B,PRKCSH,PSMC6,RAD9A,RNF19B,ROCK1,RTKN,RTP4,SDF2L1,SEC31A,SELK,SELT,SEMA3A,SERPINE1,SLAMF1,SLC1A4,SLC26A6,SLC3A2,SLC7A5,SLFN11,SP140,SRSF3,SUMO1,SYVN1,TAF9,TDP2,TMEM39A,TMEM91,TOB1,TRAF1,TRIB1,TRIB3,TRIM5,TUBB3,UBA5,UBE2T,UFD1L,UGDH,USP18,VSX2,VWF,WFS1,WIPI1,XCR1,ZC3H12A,ZFAND2A
1	chaperone-mediated protein folding	8	0.000155	CALR,FKBP11,FKBP14,FKBP2,FKBP4,FKBP5,HSPH1,PDIA4
4	single-organism cellular process	184	0.000173	ACP5,ADI1,AGPAT9,ALDH1L2,ALK,ANG,ANKRD1,ANP32A,ASNA1,ASNS,ATL1,ATMIN,ATP1B1,ATP2A2,ATXN3,B3GNT5,BANP,BATF,BCL2A1,BEX2,BORA,C12orf65,C1D,C1QTNF1,CABLES2,CALR,CARS,CAST,CCDC28B,CCL23,CCL4L2,CCNG2,CCPG1,CD164,CD276,CD36,CDC42EP3,CHST2,CISD2,CLIC2,CTNNB1,CTNS,CYB561,CYP27B1,DDIT3,DERL2,DNAAF1,DNAJB11,DNAJB9,DPYD,DTX3L,DYNC1LI1,DYRK2,EEPD1,ELOVL6,ERO1LB,EXT1,FANCE,FKBP14,FNDC3A,FPR1,FPR3,G3BP1,GABARAPL2,GADD45GIP1,GDF15,GFPT1,GMPPB,GNB4,GPR132,GPR183,GPT2,HABP4,HIAT1,HMGB1,HRSP12,HSP90B1,HSPA5,HYOU1,ID2,IRAK2,KCNK6,KIAA1279,KLF4,KPNA2,LIG4,LIMA1,LIN7C,LTBP3,LYPLA1,MAF,MATK,ME3,MED21,MEI1,METRNL,MFSD2A,MIS12,MLEC,MPLKIP,MSH2,MSTO1,MT1G,MTFR1,MTHFD2,MTMR4,NDUFA5,NFKBIA,NPLOC4,OSTC,PDE4B,PGM3,PIGA,PIK3AP1,PNP,PPP1CB,PPP1R15B,PSAT1,PSMC6,PSTPIP2,PTGES,RAD9A,RCC1,RGS2,RNF19B,ROCK1,RPLP1,RPN1,RTKN,RTP4,SDF2L1,SEC11C,SEC24D,SEC31A,SEL1L,SELK,SEMA3A,SERPINE1,SLAMF1,SLC10A7,SLC17A9,SLC1A4,SLC1A5,SLC22A16,SLC26A6,SLC35B1,SLC39A14,SLC3A2,SLC7A1,SLC7A5,SPCS2,SPCS3,SRSF3,ST6GALNAC4,SYVN1,TAF9,TARS,TCP1,TDP2,TMEM38B,TMEM91,TNFRSF4,TNFRSF9,TNFSF10,TOB1,TOPORS,TRAF1,TRIB1,TRIB3,TRIM5,TUBA3D,UAP1,UBE2T,UFD1L,UGDH,UPB1,UQCRH,USP18,VSX2,VWF,WFS1,WIPI1,WTAP,ZC3H12A
4	single-organism metabolic process	96	0.000195	AARS,ACP5,ADI1,AGPAT9,ALDH1L2,ALK,AMY2B,ANG,ANKRD1,APP,ASNS,ATF3,ATF4,ATXN3,B3GNT5,BATF,CALR,CARS,CD36,CHST2,CISD2,CLIC2,CTNS,CTR9,CYB561,CYP27B1,CYP51A1,DERL2,DHRS11,DNAJB9,DNAJC10,DPYD,DTX3L,DUSP6,EEPD1,EIF2AK3,ELOVL6,EXT1,FANCE,GABARAPL2,GADD45GIP1,GFPT1,GMPPB,GNB4,GNG10,GPT2,HCCS,HERPUD1,HMGB1,HSP90B1,HSPA5,HTRA2,IRAK2,LIG4,LYPLA1,MLEC,MSH2,MTFR1,MTHFD2,MTMR4,NDUFA5,NPLOC4,OSTC,PDE4B,PDIA5,PGM3,PIGA,PNP,PPP1CB,PSAT1,PSMC6,PTGES,RAD9A,RPN1,SDF2L1,SEC24D,SEC31A,SEL1L,SLC7A5,ST6GALNAC4,SUMO1,SYVN1,TAF9,TARS,TDP2,TRAM2,TRIB1,TRIB3,UAP1,UBE2T,UFD1L,UGDH,UPB1,VWF,WFS1,WIPI1
4	cellular response to stimulus	123	0.000315	AARS,ALK,ANKRD1,ANP32A,APP,ASNA1,ATF4,ATMIN,ATP1B1,ATP2A2,ATXN3,BATF,BCL2A1,CALR,CCL23,CCL4L2,CD164,CD36,CD38,CDC42EP3,CISD2,CLIC2,CTNNB1,CTR9,CYP27B1,CYP51A1,DDIT3,DDIT4,DERL2,DNAJB11,DNAJB9,DNAJC10,DTX3L,DUSP6,DYRK2,EBI3,EEPD1,EIF2AK3,EVL,EXT1,FAM129A,FANCE,FKBP14,FPR1,FPR3,G3BP1,GABARAPL2,GABRE,GCLC,GDF15,GFPT1,GNB4,GOT1,GPR132,GPR183,HABP4,HERPUD1,HMGB1,HSP90B1,HSPA5,HSPH1,HTRA2,HYOU1,IER3,IRAK2,KLF4,KPNA2,LARP1B,LIG4,MATK,MIS12,MSH2,MT1G,MT1H,MT1M,MTMR4,NFKBIA,NPLOC4,PDE4B,PDIA4,PDIA5,PDIA6,PIK3AP1,PPP1CB,PPP1R15B,PRKCSH,PSMC6,PTGES,RAD9A,ROCK1,RTKN,SDF2L1,SEC31A,SELK,SELT,SEMA3A,SERPINE1,SLAMF1,SLC1A4,SLC26A6,SRSF3,SUMO1,SYVN1,TAF9,TDP2,TMEM39A,TNFRSF4,TNFSF10,TOB1,TRAF1,TRIB1,TRIB3,TRIM5,UBA5,UBE2T,UFD1L,UGDH,USP18,WFS1,WIPI1,XCR1,ZC3H12A,ZFAND2A
4	single-organism transport	72	0.000417	ABAT,ADA,ALDH5A1,APP,ASNA1,ATP1B1,ATP2A2,CALR,CD36,CLIC2,CTNNB1,CTNS,CYB561,CYP27B1,DDIT3,DERL2,FABP5,FKBP4,GABARAPL2,GABRE,HABP4,HERPUD1,HIAT1,HSP90B1,HSPA5,KCNK6,KIAA1033,KIAA1279,KPNA2,LIN7C,MERTK,MFSD2A,NFKBIA,NPLOC4,PDIA4,PNP,RNF19B,RPLP1,RPN1,RTP4,SEC11C,SEC24D,SEC31A,SEL1L,SELK,SELT,SERPINE1,SLC10A7,SLC17A9,SLC1A4,SLC1A5,SLC22A16,SLC26A6,SLC35B1,SLC39A14,SLC3A2,SLC7A1,SLC7A5,SPCS2,SPCS3,SRSF11,SRSF3,SYVN1,TMEM38B,TOB1,UFD1L,UQCRH,VPS26A,VWF,WFS1,WIPI1,XCR1
1	proteasome-mediated ubiquitin-dependent protein catabolic process	18	0.000432	ATXN3,DDIT3,DERL2,DNAJB9,DNAJC10,HSP90B1,HSPA5,NPLOC4,PPP2CB,PSMC6,SDF2L1,SEL1L,SYVN1,TOPORS,UBXN1,UFD1L,USP18,WFS1
1	protein metabolic process	91	0.000448	ALG13,ALK,APP,ASNA1,ASNS,ATF3,ATF4,B3GNT5,C12orf65,CALR,CARS,CTR9,DDIT3,DERL2,DNAJA4,DNAJB5,DNAJB9,DNAJC10,DTX3L,DUSP6,DYRK2,EEF1B2,EXT1,FICD,FKBP11,FKBP14,FKBP2,FKBP4,FKBP5,GADD45GIP1,GFPT1,GMPPB,HSP90B1,HSPA5,HSPH1,HTRA2,HYOU1,IRAK2,LYPLA1,MATK,MED21,MERTK,MLEC,MTMR4,NPLOC4,OSTC,PDIA4,PDIA5,PDIA6,PELI1,PFDN2,PGM3,PIGA,PIM2,PPIG,PPIL1,PPP1CB,PRPF4B,PSMC6,RNF19B,ROCK1,RPLP1,RPN1,SDF2L1,SEC24D,SEC31A,SEL1L,SELT,SIL1,ST6GALNAC4,SUMO1,SYVN1,TAF9,TARS,TCP1,TNFSF10,TOPORS,TRIB1,TRIB3,TRIM5,TUBA3D,TUBB3,UAP1,UBA5,UBE2T,UBXN1,UFD1L,USP18,VWF,WFS1,WIPI1
4	cellular process	212	0.000551	ACP5,ADI1,AGPAT9,ALDH1L2,ALK,ANG,ANKRD1,ANP32A,ASNA1,ATF4,ATF7IP2,ATL1,ATMIN,ATP1B1,ATP2A2,ATXN3,B3GNT5,BANP,BCAS2,BCL2A1,BEX2,BORA,C12orf65,C1D,CABLES2,CALR,CARS,CAST,CCDC28B,CCL23,CCL4L2,CCNG2,CCPG1,CD164,CD276,CD36,CDC42EP3,CHST2,CISD2,CLIC2,COMMD8,CTNNB1,CTNS,CYB561,CYP27B1,CYP51A1,DDIT3,DDX17,DERL2,DNAAF1,DNAJA4,DNAJB5,DNAJB9,DPYD,DTX3L,DYNC1LI1,DYRK2,EEF1B2,EEPD1,EIF2AK3,ELOVL6,EXT1,FAM129A,FANCE,FICD,FKBP11,FKBP14,FKBP2,FKBP5,FNDC3A,FPR1,FPR3,G3BP1,GABARAPL2,GADD45GIP1,GDF15,GFPT1,GMPPB,GNB4,GPR132,GPR183,GPT2,HABP4,HIAT1,HMGB1,HRSP12,HSP90B1,HSPA5,HYOU1,ID2,IRAK2,KCNK6,KCTD15,KIAA1033,KIAA1279,KLF4,KPNA2,LARP1B,LARP4,LIG4,LIMA1,LIN7C,LTBP3,MATK,MBNL2,ME3,MEI1,METRNL,MFSD2A,MIS12,MLEC,MPLKIP,MPV17L2,MSH2,MSTO1,MT1G,MT1H,MT1M,MTFR1,MTHFD2,MTMR4,N4BP2,NDUFA5,NFKBIA,NFXL1,NPLOC4,OSTC,PC,PDE4B,PDIA4,PDIA5,PDIA6,PFDN2,PIGA,PIK3AP1,PNP,PPIG,PPIL1,PPP1CB,PRPF4B,PSAT1,PSMC6,PSTPIP2,PTGES,PTMA,RAD9A,RCC1,RGS2,RNASE4,RNF19B,ROCK1,RPLP1,RPN1,RTP4,SDF2L1,SEC24D,SEC31A,SELK,SEMA3A,SERPINE1,SERTAD3,SIL1,SLAMF1,SLC10A7,SLC17A9,SLC1A4,SLC1A5,SLC22A16,SLC26A6,SLC35B1,SLC39A14,SLC3A2,SLC7A1,SLC7A5,SP140,SPOCD1,SRSF11,SRSF3,ST6GALNAC4,SYVN1,TAF9,TARS,TDP2,TMEM38B,TMEM39A,TMEM91,TNFRSF4,TNFSF10,TOB1,TOPORS,TRIB1,TRIB3,TRIM5,TSN,UAP1,UBA5,UBE2T,UBXN1,UFD1L,UGDH,UPB1,UQCRH,USP18,VSX2,VWF,WFS1,WIPI1,WTAP,ZC3H12A,ZFAND2A,ZNF395,ZNF768
4	single-organism process	188	0.000647	ADI1,AGPAT9,ALDH1L2,ALK,AMY2B,ANG,ANKRD1,ANP32A,ASNA1,ATL1,ATMIN,ATP1B1,ATP2A2,ATXN3,B3GNT5,BANP,BATF,BCL2A1,BEX2,BORA,C12orf65,C1D,C1QTNF1,CABLES2,CALR,CARS,CAST,CCDC28B,CCL23,CCL4L2,CCNG2,CCPG1,CD164,CD276,CD36,CDC42EP3,CHST2,CLIC2,CRELD1,CTNNB1,CYB561,CYP27B1,CYP51A1,DDIT3,DERL2,DHRS11,DNAAF1,DNAJB11,DNAJB9,DPYD,DTX3L,DYNC1LI1,DYRK2,EEPD1,ELOVL6,EXT1,FANCE,FGFBP3,FKBP14,FNDC3A,FPR1,FPR3,G3BP1,GABARAPL2,GADD45GIP1,GDF15,GFPT1,GMPPB,GNB4,GNL3,GPR132,GPR183,GPT2,HABP4,HCCS,HIAT1,HMGB1,HRSP12,HSP90B1,HSPA5,HYOU1,ID2,IRAK2,KCTD15,KIAA1033,KIAA1279,KLF4,KPNA2,LIG4,LIMA1,LIN7C,LTBP3,LYPLA1,MAF,MATK,MED21,MEI1,MERTK,METRNL,MFSD2A,MIS12,MLEC,MPLKIP,MSH2,MSTO1,MT1G,MTFR1,MTHFD2,MTMR4,NDUFA5,NFKBIA,NPLOC4,OSTC,PDE4B,PIGA,PIK3AP1,PNP,PPP1R15B,PSAT1,PSMC6,PSTPIP2,PTGES,RAD9A,RCC1,RGS2,RNF19B,ROCK1,RPLP1,RPN1,RTKN,RTP4,SDF2L1,SEC11C,SEC31A,SEL1L,SELK,SEMA3A,SERPINE1,SLAMF1,SLC10A7,SLC17A9,SLC1A4,SLC1A5,SLC22A16,SLC26A6,SLC35B1,SLC39A14,SLC3A2,SLC7A1,SLC7A5,SPCS2,SPCS3,SRSF11,SRSF3,ST6GALNAC4,SYVN1,TAF9,TARS,TCP1,TDP2,TMEM38B,TMEM41B,TMEM91,TNFRSF4,TNFRSF9,TNFSF10,TOB1,TOPORS,TRAF1,TRAM2,TRIB1,TRIB3,TRIM5,TUBA3D,UAP1,UBE2T,UFD1L,UGDH,UPB1,UQCRH,USP18,VPS26A,VSX2,VWF,WFS1,WIPI1,WTAP,ZC3H12A

	4	single-organism localization	74	0.000839	ABAT,ADA,ALDH5A1,APP,ASNA1,ATP1B1,ATP2A2,CALR,CD36,CLIC2,CTNNB1,CTNS,CYB561,CYP27B1,DDIT3,DERL2,FABP5,FKBP4,GABARAPL2,GABRE,HABP4,HERPUD1,HIAT1,HSP90B1,HSPA5,KCNK6,KIAA1033,KIAA1279,KPNA2,LIN7C,MERTK,MFSD2A,MSTO1,NFKBIA,NPLOC4,PDIA4,PNP,RNF19B,RPLP1,RPN1,RTP4,SEC11C,SEC24D,SEC31A,SEL1L,SELK,SELT,SERPINE1,SLC10A7,SLC17A9,SLC1A4,SLC1A5,SLC22A16,SLC26A6,SLC35B1,SLC39A14,SLC3A2,SLC7A1,SLC7A5,SPCS2,SPCS3,SRSF11,SRSF3,SYVN1,TMEM38B,TOB1,TOPORS,UFD1L,UQCRH,VPS26A,VWF,WFS1,WIPI1,XCR1
	4	establishment of localization in cell	50	0.000925	ABAT,ALDH5A1,ANP32A,APP,ATP1B1,ATP2A2,CALR,CD36,CTNNB1,DDIT3,DERL2,EIF2AK3,GABARAPL2,HABP4,HERPUD1,HSP90B1,HSPA5,KIAA1033,KIAA1279,KPNA2,LIN7C,MERTK,NFKBIA,NPLOC4,PDIA4,PNP,RNF19B,RPLP1,RPN1,RTP4,SAR1A,SEC11C,SEC31A,SEL1L,SELT,SERPINE1,SIL1,SLC17A9,SPCS2,SPCS3,SRSF11,SRSF3,SYVN1,TOB1,UFD1L,VPS26A,VWF,WFS1,WIPI1,XCR1
	1	protein peptidyl-prolyl isomerization	7	0.000938	FKBP11,FKBP14,FKBP2,FKBP4,FKBP5,PPIG,PPIL1
	1	transport	80	0.001	ABAT,ADA,ALDH5A1,ANP32A,APP,ASNA1,ATP1B1,ATP2A2,CALR,CD163,CD36,CLIC2,CTNNB1,CTNS,CYB561,CYP27B1,DDIT3,DERL2,DYNC1LI1,FABP5,FKBP4,G3BP1,GABARAPL2,HABP4,HERPUD1,HIAT1,HSP90B1,HSPH1,HYOU1,KCNK6,KIAA1033,KIAA1279,LIN7C,LY75,MERTK,MFSD2A,NFKBIA,NPLOC4,PDIA4,PNP,RNF19B,RPLP1,RPN1,RTP4,SAR1A,SEC11C,SEC31A,SEL1L,SELK,SELT,SERPINE1,SIL1,SLC10A7,SLC17A9,SLC1A4,SLC1A5,SLC22A16,SLC26A6,SLC35B1,SLC39A14,SLC3A2,SLC7A1,SLC7A5,SPCS2,SPCS3,SRSF11,SRSF3,SYVN1,TCP1,TMED7,TMEM38B,TOB1,TRAM2,UFD1L,UQCRH,VPS26A,VWF,WFS1,WIPI1,XCR1
	1	nucleotide-sugar biosynthetic process	5	0.00117	GFPT1,GMPPB,PGM3,UAP1,UGDH
	1	protein folding in endoplasmic reticulum	4	0.00129	CALR,DNAJC10,HSP90B1,HSPA5
	4	negative regulation of biological process	93	0.00153	AARS,ACP5,ALOX15B,ANG,ANKRD1,ASNS,ATP2A2,BANP,BCL2A1,C1D,C1QTNF1,CALR,CAPZA1,CAST,CCL23,CD164,CD276,CD36,CLIC2,CTNNB1,CTR9,CYP27B1,DDIT3,DERL2,DNAJA4,DNAJB5,DNAJC10,DUSP6,DYRK2,EVI5L,EVL,FAM129A,FICD,G3BP1,GABARAPL2,GADD45GIP1,GPR132,HMGB1,HRSP12,HSP90B1,HSPH1,HTRA2,IRAK2,KLF4,LIG4,LIMA1,LYPLA1,MAF,MED21,MERTK,METRNL,MSH2,MT1G,MT1H,MT1M,MTMR4,NFKBIA,NFXL1,PC,PDE4B,PELI1,PIK3AP1,PIM2,PPP1CB,PPP1R15B,PSMC6,PTGES,RAD9A,RAP1B,RGS2,RTKN,SAR1A,SEMA3A,SERPINE1,SERTAD3,SLFN11,SPOCD1,SUMO1,SYVN1,TAF9,TDP2,TES,TNFRSF4,TNFSF10,TOPORS,TRIB1,TRIB3,TRIM5,UBXN1,VSX2,WFS1,XCR1,ZC3H12A
	1	cell redox homeostasis	8	0.00175	DDIT3,DNAJC10,ERO1LB,GCLC,PDIA4,PDIA5,PDIA6,SELT
	1	ATF6-mediated unfolded protein response	4	0.00214	CALR,DDIT3,HSP90B1,HSPA5
	4	localization	93	0.00255	ABAT,ADA,ALDH5A1,ANG,ANP32A,APP,ASNA1,ATP1B1,ATP2A2,BANP,CALR,CCL23,CCL4L2,CD163,CD36,CLIC2,CTNNB1,CTNS,CYB561,CYP27B1,DDIT3,DDIT4,DERL2,DYNC1LI1,FABP5,FKBP4,G3BP1,GABARAPL2,HABP4,HERPUD1,HIAT1,HMGB1,HSP90B1,HSPH1,HYOU1,KCNK6,KIAA1033,KIAA1279,LIN7C,LY75,MATK,MERTK,MFSD2A,MSTO1,NFKBIA,NPLOC4,PDE4B,PDIA4,PNP,PSTPIP2,RNF19B,ROCK1,RPLP1,RPN1,RTP4,SAR1A,SEC11C,SEC31A,SEL1L,SELK,SELT,SEMA3A,SERPINE1,SIL1,SLC10A7,SLC17A9,SLC1A4,SLC1A5,SLC22A16,SLC26A6,SLC35B1,SLC39A14,SLC3A2,SLC7A1,SLC7A5,SPCS2,SPCS3,SRSF11,SRSF3,SYVN1,TCP1,TMED7,TMEM38B,TOB1,TOPORS,TRAM2,UFD1L,UQCRH,VPS26A,VWF,WFS1,WIPI1,XCR1
	3	intrinsic apoptotic signaling pathway	12	0.00255	ATF4,BCL2A1,DDIT3,DDIT4,DNAJC10,DYRK2,EIF2AK3,HTRA2,IER3,MSH2,SELK,TRIB3
	1	negative regulation of sequence-specific DNA binding transcription factor activity	11	0.00261	DDIT3,ID2,IRAK2,KLF4,NFKBIA,PELI1,SUMO1,TNFRSF4,TRIB1,WFS1,ZC3H12A
3	1	intrinsic apoptotic signaling pathway in response to endoplasmic reticulum stress	6	0.00261	ATF4,DDIT3,DNAJC10,EIF2AK3,SELK,TRIB3
	3	regulation of apoptotic process	41	0.00262	AARS,ADA,ALK,ANKRD1,ASNS,ATF3,ATF4,BCL2A1,BEX2,CALR,CAST,CD38,CTNNB1,DDIT3,DUSP6,GDF15,HMGB1,HSP90B1,HSPA5,HSPH1,HTRA2,KLF4,LIG4,MERTK,MSH2,NFKBIA,PIM2,PPP1CB,PSMC6,RAD9A,ROCK1,SDF2L1,SERPINE1,SYVN1,TAF9,TCP1,TNFRSF4,TNFSF10,TOPORS,TRAF1,WFS1
	1	peptidyl-amino acid modification	30	0.00292	ALG13,ALK,CALR,CTR9,DYRK2,EIF2AK3,FKBP11,FKBP14,FKBP2,FKBP4,FKBP5,GFPT1,GMPPB,MATK,MERTK,MLEC,OSTC,PGM3,PPIG,PPIL1,PRKCSH,RPN1,SEC24D,SEC31A,ST6GALNAC4,SUMO1,SYVN1,TAF9,TOPORS,UAP1
	4	cellular amino acid metabolic process	17	0.00294	AARS,ADI1,ALDH1L2,ALDH5A1,ASNS,ATF4,CARS,CTNS,DPYD,ERO1LB,GCLC,GFPT1,GPT2,PSAT1,SLC7A5,TARS,UPB1
	4	response to biotic stimulus	26	0.0042	ACP5,ANKRD1,BATF,CD36,CTR9,CYP27B1,DDIT3,DDIT4,EIF2AK3,HSPA5,IER3,IRAK2,NFKBIA,PDE4B,PELI1,PIM2,PPP1R15B,PTGES,SERPINE1,SLFN11,TLR1,TMEM91,TRIB1,TRIM5,WFS1,ZC3H12A
	4	cellular localization	54	0.00461	ABAT,ALDH5A1,ANP32A,APP,ASNA1,ATP1B1,ATP2A2,BANP,CALR,CD36,CTNNB1,DDIT3,DERL2,EIF2AK3,GABARAPL2,HABP4,HERPUD1,HSP90B1,HSPA5,KIAA1033,KIAA1279,KPNA2,LIN7C,MERTK,MSTO1,NFKBIA,NPLOC4,PDIA4,PNP,RNF19B,RPLP1,RPN1,RTP4,SAR1A,SEC11C,SEC31A,SEL1L,SELT,SERPINE1,SIL1,SLC17A9,SPCS2,SPCS3,SRSF11,SRSF3,SYVN1,TOB1,TOPORS,UFD1L,VPS26A,VWF,WFS1,WIPI1,XCR1
	3	regulation of cell death	42	0.00471	AARS,ADA,ALK,ANKRD1,ASNS,ATF3,ATF4,BCL2A1,BEX2,CALR,CAST,CD38,CTNNB1,DDIT3,DDIT4,DUSP6,GDF15,HMGB1,HSP90B1,HSPA5,HSPH1,HTRA2,KLF4,LIG4,MERTK,MSH2,NFKBIA,PIM2,PPP1CB,PSMC6,RAD9A,ROCK1,SDF2L1,SERPINE1,SYVN1,TAF9,TCP1,TNFRSF4,TNFSF10,TOPORS,TRAF1,WFS1
	1	cellular amino acid biosynthetic process	8	0.00499	ABAT,ADI1,ASNS,DPYD,GPT2,MTHFD2,PSAT1,UPB1
	4	negative regulation of cellular process	85	0.00601	AARS,ACP5,ALOX15B,ANG,ANKRD1,ASNS,ATF4,BCL2A1,C1D,C1QTNF1,CALR,CAPZA1,CAST,CCL23,CD164,CD276,CD36,CLIC2,CTNNB1,CTR9,CYP27B1,DDIT3,DERL2,DNAJA4,DNAJB5,DNAJC10,DUSP6,DYRK2,EVI5L,EVL,FAM129A,G3BP1,GABARAPL2,GADD45GIP1,GPR132,HMGB1,HRSP12,HSP90B1,HSPH1,HTRA2,IRAK2,KLF4,LIG4,LIMA1,LYPLA1,MAF,MED21,MERTK,MSH2,MTMR4,NFKBIA,NFXL1,PELI1,PIK3AP1,PIM2,PPP1CB,PPP1R15B,PSMC6,PTGES,RAD9A,RAP1B,RGS2,ROCK1,SAR1A,SEMA3A,SERPINE1,SERTAD3,SLFN11,SPOCD1,SUMO1,SYVN1,TAF9,TDP2,TES,TNFRSF4,TNFSF10,TOPORS,TRIB1,TRIB3,TRIM5,UBXN1,VSX2,WFS1,XCR1,ZC3H12A
	1	positive regulation of transcription from RNA polymerase II promoter in response to endoplasmic reticulum stress	4	0.00605	ATF3,ATF4,DDIT3,HSPA5
	1	single-organism carbohydrate metabolic process	23	0.00637	ALDH5A1,ATF3,ATF4,B3GNT5,CALR,EXT1,FABP5,GCLC,GFPT1,GMPPB,GOT1,MLEC,OSTC,PC,PGM3,PPP1CB,RPN1,SEC24D,SEC31A,ST6GALNAC4,SYVN1,UAP1,UGDH
	4	cellular metabolic process	152	0.00679	ACP5,ADI1,AGPAT9,ALDH1L2,ALK,ANG,ANKRD1,ANP32A,APP,ASNA1,ASNS,ATF3,ATF7IP2,ATMIN,ATXN3,B3GNT5,BANP,BCAS2,C12orf65,C1D,CALR,CARS,CD36,CHST2,CISD2,COMMD8,CTNNB1,CTNS,CTR9,CYB561,CYP27B1,CYP51A1,DDIT3,DDX17,DERL2,DNAJA4,DNAJB5,DNAJB9,DNAJC10,DPYD,DTX3L,DUSP6,DYRK2,EEF1B2,EEPD1,ELOVL6,EXT1,FANCE,FICD,FKBP11,FKBP14,FKBP2,FKBP4,FKBP5,G3BP1,GABARAPL2,GADD45GIP1,GFPT1,GMPPB,GNB4,GNG10,GPT2,HABP4,HMGB1,HRSP12,HSP90B1,HSPA5,HSPH1,HYOU1,ID2,IRAK2,KLF4,KPNA2,LIG4,LMO4,MATK,MBNL2,ME3,MERTK,MLEC,MSH2,MTFR1,MTHFD2,MTMR4,N4BP2,NDUFA5,NFXL1,NPLOC4,OSTC,PDE4B,PDIA4,PDIA5,PDIA6,PELI1,PFDN2,PIGA,PIM2,PNP,PPIG,PPIL1,PPP1CB,PRPF4B,PSAT1,PSMC6,PTGES,PTMA,RAD9A,RNASE4,RNF19B,ROCK1,RPLP1,RPN1,SDF2L1,SEC24D,SEC31A,SEL1L,SELT,SERPINE1,SERTAD3,SIL1,SLC7A5,SPOCD1,SRSF11,SRSF3,ST6GALNAC4,SUMO1,SYVN1,TAF9,TARS,TCP1,TDP2,TOPORS,TRIB1,TRIM5,TSN,TUBA3D,TUBB3,UAP1,UBA5,UBE2T,UBXN1,UFD1L,UGDH,UPB1,USP18,VSX2,WFS1,WIPI1,WTAP,ZC3H12A,ZNF395,ZNF768
	1	positive regulation of proteasomal protein catabolic process	8	0.00679	ATXN3,GCLC,HERPUD1,PSMC6,SUMO1,TRIB1,TRIB3,ZFAND2A

	1	ubiquitin-dependent protein catabolic process	19	0.00715	ATXN3,DDIT3,DERL2,DNAJB9,DNAJC10,HERPUD1,HSP90B1,HSPA5,NPLOC4,PPP2CB,PSMC6,SDF2L1,SEL1L,SYVN1,TOPORS,UBXN1,UFD1L,USP18,WFS1
	4	organic substance transport	49	0.00742	ADA,ATP1B1,CALR,CD36,CTNS,DERL2,EIF2AK3,FABP5,GABARAPL2,HERPUD1,HSP90B1,KIAA1033,LIN7C,MFSD2A,NFKBIA,NPLOC4,PDIA4,PNP,RNF19B,RPLP1,RPN1,RTP4,SAR1A,SEC11C,SEC24D,SEC31A,SEL1L,SELT,SIL1,SLC1A4,SLC1A5,SLC22A16,SLC26A6,SLC35B1,SLC3A2,SLC7A1,SLC7A5,SPCS2,SPCS3,SRSF11,SRSF3,SYVN1,TCP1,TMED7,TOB1,TRAM2,UFD1L,VPS26A,WFS1
	1	UDP-N-acetylglucosamine biosynthetic process	3	0.00768	GFPT1,PGM3,UAP1
	1	protein glycosylation	15	0.00911	ALG13,B3GNT5,CALR,EXT1,GFPT1,GMPPB,MLEC,OSTC,PGM3,RPN1,SEC24D,SEC31A,ST6GALNAC4,SYVN1,UAP1
	1	regulation of binding	14	0.0099	APP,CCL23,CLIC2,DDIT3,EIF2AK3,HABP4,HERPUD1,HMGB1,HSPA5,ID2,NFKBIA,ROCK1,SUMO1,TRIB3
	3	programmed cell death	33	0.0101	ALOX15B,APP,ATF4,BCL2A1,BEX2,C1D,CD38,CTNNB1,DDIT3,DDIT4,DNAJC10,DYRK2,GCLC,HERPUD1,HMGB1,HTRA2,KLF4,LIG4,MSH2,NFKBIA,PIM2,PPP2CB,PSMC6,ROCK1,RTKN,SELK,SEMA3A,TNFRSF4,TNFRSF9,TNFSF10,TRAF1,TRIB3,ZC3H12A
	4	cellular macromolecule metabolic process	127	0.0101	ALG13,ALK,ANG,ANKRD1,ANP32A,APP,ASNA1,ASNS,ATF3,ATF7IP2,ATMIN,ATXN3,B3GNT5,BANP,BCAS2,C12orf65,C1D,CALR,CARS,COMMD8,CTNNB1,CTR9,DDIT3,DDIT4,DDX17,DERL2,DNAJA4,DNAJB5,DNAJB9,DNAJC10,DTX3L,DUSP6,DYRK2,EEF1B2,EEPD1,EXT1,FANCE,FICD,FKBP11,FKBP14,FKBP2,FKBP4,FKBP5,GADD45GIP1,GFPT1,GMPPB,HABP4,HMGB1,HRSP12,HSP90B1,HSPA5,HSPH1,HTRA2,HYOU1,ID2,IRAK2,KLF4,KPNA2,LIG4,LMO4,LYPLA1,MATK,MBNL2,MERTK,MLEC,MSH2,MTMR4,NFXL1,NPLOC4,OSTC,PDIA4,PDIA5,PDIA6,PELI1,PFDN2,PGM3,PIGA,PIM2,PPIG,PPIL1,PPP1CB,PRPF4B,PSMC6,PTMA,RAD9A,RNASE4,RNF19B,ROCK1,RPLP1,RPN1,SDF2L1,SEC24D,SEC31A,SEL1L,SELT,SERPINE1,SERTAD3,SIL1,SPOCD1,SRSF11,SRSF3,ST6GALNAC4,SUMO1,SYVN1,TAF9,TARS,TCP1,TDP2,TOPORS,TRIB1,TRIB3,TRIM5,TSN,TUBA3D,TUBB3,UAP1,UBA5,UBE2T,UBXN1,UFD1L,USP18,VSX2,WFS1,WIPI1,WTAP,ZNF395,ZNF768
3	1	regulation of endoplasmic reticulum stress-induced intrinsic apoptotic signaling pathway	5	0.0105	DDIT3,HERPUD1,HYOU1,SYVN1,WFS1
	4	cytoplasmic transport	25	0.0113	ANP32A,ATP2A2,CALR,DDIT3,DERL2,HERPUD1,HSP90B1,KPNA2,NFKBIA,NPLOC4,RPLP1,RPN1,SEC11C,SEC24D,SEC31A,SEL1L,SPCS2,SPCS3,SRSF11,SRSF3,SYVN1,TOB1,UFD1L,WIPI1,XCR1
	1	cellular protein catabolic process	20	0.0131	ATXN3,DDIT3,DERL2,DNAJB9,DNAJC10,HERPUD1,HSP90B1,HSPA5,HTRA2,NPLOC4,PPP2CB,PSMC6,SDF2L1,SEL1L,SYVN1,TOPORS,UBXN1,UFD1L,USP18,WFS1
	3	regulation of apoptotic signaling pathway	17	0.0137	ATF3,BCL2A1,CTNNB1,DDIT3,GCLC,HERPUD1,HSPH1,HTRA2,IER3,PPP1CB,RAD9A,SERPINE1,SYVN1,TAF9,TNFSF10,TRAF1,WFS1
3	1	positive regulation of proteasomal ubiquitin-dependent protein catabolic process	7	0.0163	ATXN3,GCLC,HERPUD1,SUMO1,TRIB1,TRIB3,ZFAND2A
3	1	negative regulation of endoplasmic reticulum stress-induced intrinsic apoptotic signaling pathway	4	0.0163	HERPUD1,HYOU1,SYVN1,WFS1
	1	negative regulation of response to endoplasmic reticulum stress	5	0.0163	DERL2,HERPUD1,HYOU1,PPP1R15B,SYVN1
	2	toll-like receptor signaling pathway	9	0.0168	CD36,DUSP6,HSP90B1,IRAK2,NFKBIA,PELI1,PIK3AP1,TANK,TLR1
	4	carbohydrate metabolic process	26	0.0171	ALDH5A1,AMY2B,ATF3,ATF4,B3GNT5,CALR,CHST2,EXT1,FABP5,GCLC,GFPT1,GMPPB,GOT1,MLEC,OSTC,PC,PGM3,PPP1CB,RPN1,SEC24D,SEC31A,SLC3A2,ST6GALNAC4,SYVN1,UAP1,UGDH
	3	apoptotic process	32	0.0171	ALOX15B,APP,ATF4,BCL2A1,BEX2,C1D,CD38,CTNNB1,DDIT3,DDIT4,DNAJC10,DYRK2,GCLC,HERPUD1,HMGB1,HTRA2,KLF4,LIG4,MSH2,NFKBIA,PIM2,PPP2CB,PSMC6,ROCK1,RTKN,SELK,SEMA3A,TNFRSF9,TNFSF10,TRAF1,TRIB3,ZC3H12A
	1	protein transport	35	0.0171	ATP1B1,CALR,CD36,DERL2,EIF2AK3,GABARAPL2,HERPUD1,HSP90B1,KIAA1033,LIN7C,NFKBIA,NPLOC4,PDIA4,PNP,RNF19B,RPLP1,RPN1,RTP4,SAR1A,SEC11C,SEC24D,SEC31A,SEL1L,SELT,SIL1,SPCS2,SPCS3,SYVN1,TCP1,TMED7,TOB1,TRAM2,UFD1L,VPS26A,WFS1
	2	B cell activation involved in immune response	5	0.0179	ADA,BATF,GPR183,LIG4,MSH2
	2	response to molecule of bacterial origin	14	0.0187	ACP5,ANKRD1,CD36,CTR9,CYP27B1,IRAK2,NFKBIA,PDE4B,PELI1,PTGES,SERPINE1,TLR1,TRIB1,ZC3H12A
	4	organic acid transmembrane transport	9	0.0187	CD36,CTNS,SLC1A4,SLC1A5,SLC22A16,SLC26A6,SLC3A2,SLC7A1,SLC7A5
	1	positive regulation of protein metabolic process	38	0.0192	ALK,ANG,APP,ATXN3,C1QTNF1,CD276,CD36,CTNNB1,CTR9,EBI3,EIF2AK3,FAM129A,FPR1,GCLC,GDF15,GPR183,HERPUD1,HMGB1,HSPA5,HSPH1,HTRA2,IER3,IRAK2,KLF4,MPV17L2,NFKBIA,PELI1,PSMC6,RAP1B,SUMO1,TLR1,TNFSF10,TOPORS,TRIB1,TRIB3,TRIM5,WFS1,ZFAND2A
	4	regulation of cellular catabolic process	24	0.0201	ATP1B1,ATXN3,CISD2,DDIT4,GABARAPL2,GCLC,HERPUD1,HTRA2,IER3,LARP1B,PIM2,PSMC6,SLC17A9,SLC1A4,SUMO1,TAF9,TMEM39A,TOB1,TRIB1,TRIB3,UBXN1,USP18,ZC3H12A,ZFAND2A
	1	endoplasmic reticulum calcium ion homeostasis	4	0.0223	APP,ATP2A2,HERPUD1,WFS1
	4	biological_process	207	0.023	ACN9,ADI1,AGPAT9,ALDH1L2,ALK,AMY2B,ANG,ANKRD1,ANP32A,ARHGEF10L,ASNA1,ATF4,ATF7IP2,ATL1,ATP1B1,ATP2A2,BANP,BCAS2,BCL2A1,BEX2,BORA,C12orf65,CABLES2,CALR,CARS,CAST,CCDC28B,CCL23,CCL4L2,CD163,CD164,CD36,CDC42EP3,CHST2,CLIC2,COMMD8,CRELD1,CTNNB1,CYB561,CYP27B1,CYP51A1,DDIT3,DDX17,DHRS11,DNAAF1,DNAJB9,DPYD,DTX3L,DYRK2,EEF1B2,EEPD1,EIF2AK3,ENTPD7,EVI5L,EXT1,FAM129A,FANCE,FGFBP3,FICD,FKBP11,FKBP14,FKBP2,FKBP5,FNDC3A,FPR1,FPR3,FZD2,GABARAPL2,GADD45GIP1,GFPT1,GMPPB,GNB4,GNL3,GPR132,GPR183,GPT2,HABP4,HCCS,HIAT1,HMGB1,HSP90B1,HSPA5,HSPH1,ID2,IRAK2,ISOC2,KCTD15,KIAA1033,KIAA1279,KLF4,LARP1B,LARP4,LIG4,LIMA1,LIN7C,LTBP3,LY75,LY9,LYPLA1,MANF,MATK,MBNL2,MEI1,MERTK,METRNL,MFSD2A,MIS12,MLEC,MPLKIP,MPV17L2,MSH2,MT1G,MT1H,MT1M,MTFR1,MTHFD2,N4BP2,NDUFA5,NFKBIA,NFXL1,NPLOC4,OSTC,PC,PDE4B,PDIA4,PDIA5,PDIA6,PFDN2,PIGA,PNP,PPIG,PPIL1,PPP1CB,PRPF4B,PSAT1,PSMC6,PSTPIP2,PTMA,RCC1,RGS2,RNASE4,RNF19B,ROCK1,RPLP1,RTKN,RTP4,SAR1A,SDF2L1,SEC31A,SELK,SEMA3A,SERPINE1,SIL1,SLA,SLC10A7,SLC17A9,SLC1A4,SLC1A5,SLC22A16,SLC26A6,SLC35B1,SLC39A14,SLC3A2,SLC7A1,SLC7A5,SLFN11,SP140,SRSF11,SRSF3,ST6GALNAC4,SUMO1,SYVN1,TAF9,TARS,TDP2,TES,TMED7,TMEM38B,TMEM39A,TMEM41B,TMEM91,TNFRSF4,TOPORS,TRAM2,TRIB1,TRIB3,TRIM5,TSN,UAP1,UBA5,UBE2T,UBXN1,UFD1L,UGDH,UPB1,UQCRH,USP18,VPS26A,VSX2,VWF,WFS1,WIPI1,WTAP,ZC3H12A,ZFAND2A,ZNF395,ZNF768
	4	cell activation	22	0.0241	APP,BATF,CAST,CD276,CD36,CTNNB1,EBI3,GPR183,HABP4,HMGB1,HSPA5,ID2,LIG4,MERTK,MSH2,MT1G,RAP1B,SERPINE1,SLAMF1,TLR1,TNFRSF4,VWF
	4	establishment of protein localization	36	0.0246	ASNA1,ATP1B1,CALR,CD36,DERL2,EIF2AK3,GABARAPL2,HERPUD1,HSP90B1,KIAA1033,LIN7C,NFKBIA,NPLOC4,PDIA4,PNP,RNF19B,RPLP1,RPN1,RTP4,SAR1A,SEC11C,SEC24D,SEC31A,SEL1L,SELT,SIL1,SPCS2,SPCS3,SYVN1,TCP1,TMED7,TOB1,TRAM2,UFD1L,VPS26A,WFS1
	1	regulation of proteasomal protein catabolic process	12	0.0246	ATXN3,GABARAPL2,GCLC,HERPUD1,PSMC6,SUMO1,TAF9,TRIB1,TRIB3,UBXN1,USP18,ZFAND2A
	2	immune response	36	0.0248	ADA,APP,BATF,CAPZA1,CCL23,CCL4L2,CD164,CD276,CD36,CTNNB1,CYP27B1,DUSP6,EBI3,GPR183,HMGB1,HSP90B1,IRAK2,LIG4,LY75,MATK,MSH2,NFKBIA,PELI1,PNP,PRKCSH,PSMC6,RNF19B,SLC26A6,SUMO1,TANK,TLR1,TNFRSF4,TNFSF10,TRIB3,TRIM5,USP18

4	regulation of catabolic process	26	0.0248	ATP1B1,ATXN3,BANP,CISD2,DDIT4,DERL2,GABARAPL2,GCLC,HERPUD1,HTRA2,IER3,LARP1B,PIM2,PSMC6,SLC17A9,SLC1A4,SUMO1,TAF9,TMEM39A,TOB1,TRIB1,TRIB3,UBXN1,USP18,ZC3H12A,ZFAND2A
4	macromolecule metabolic process	133	0.0248	ALG13,ALK,ANG,ANKRD1,ANP32A,APP,ASNA1,ASNS,ATF3,ATF7IP2,ATMIN,B3GNT5,BANP,BCAS2,C12orf65,C1D,CALR,CARS,CHST2,COMMD8,CTNNB1,CTR9,DDIT3,DDIT4,DDX17,DERL2,DNAJA4,DNAJB5,DNAJB9,DNAJC10,DTX3L,DUSP6,DYRK2,EEF1B2,EEPD1,EXT1,FANCE,FICD,FKBP11,FKBP14,FKBP2,FKBP4,FKBP5,G3BP1,GADD45GIP1,GFPT1,GMPPB,HABP4,HMGB1,HRSP12,HSP90B1,HSPA5,HSPH1,HTRA2,HYOU1,ID2,IRAK2,KLF4,KPNA2,LIG4,LMO4,MATK,MBNL2,MERTK,MLEC,MSH2,MTMR4,N4BP2,NFXL1,NPLOC4,OSTC,PDIA4,PDIA5,PDIA6,PELI1,PFDN2,PGM3,PIGA,PIM2,PPIG,PPIL1,PPP1CB,PRPF4B,PSMC6,PTMA,RAD9A,RNASE4,RNF19B,ROCK1,RPLP1,RPN1,SDF2L1,SEC24D,SEC31A,SEL1L,SELT,SERPINE1,SERTAD3,SIL1,SPOCD1,SRSF11,SRSF3,ST6GALNAC4,SUMO1,SYVN1,TAF9,TARS,TCP1,TDP2,TNFSF10,TOPORS,TRAM2,TRIB1,TRIB3,TRIM5,TSN,TUBA3D,TUBB3,UAP1,UBA5,UBE2T,UBXN1,UFD1L,UGDH,USP18,VSX2,VWF,WFS1,WIPI1,WTAP,ZC3H12A,ZNF395,ZNF768
3	negative regulation of neuron death	10	0.0248	AARS,ATF4,CTNNB1,GCLC,HTRA2,HYOU1,LIG4,MSH2,ROCK1,WFS1
4	single-organism biosynthetic process	36	0.0254	ABAT,ADI1,AGPAT9,ALOX15B,ANG,ASNS,ATF3,ATF4,B3GNT5,CHST2,CTNS,CYP27B1,CYP51A1,DPYD,ELOVL6,EXT1,FABP5,GADD45GIP1,GCLC,GFPT1,GMPPB,GPT2,MTHFD2,MTMR4,NPLOC4,PC,PGM3,PIGA,PNP,PSAT1,PTGES,TRAM2,UAP1,UFD1L,UGDH,UPB1
1	post-translational protein modification	15	0.0256	ALG13,B3GNT5,CALR,GFPT1,GMPPB,MLEC,PGM3,PIGA,PRKCSH,RPN1,SEC24D,SEC31A,ST6GALNAC4,SUMO1,UAP1
4	signal peptide processing	3	0.0263	SEC11C,SPCS2,SPCS3
1	protein catabolic process	20	0.0281	ATXN3,DDIT3,DERL2,DNAJB9,DNAJC10,HSP90B1,HSPA5,HTRA2,LYPLA1,NPLOC4,PPP2CB,PSMC6,SDF2L1,SEL1L,SYVN1,TOPORS,UBXN1,UFD1L,USP18,WFS1
1	glycosylation	14	0.0281	B3GNT5,CALR,EXT1,GFPT1,GMPPB,MLEC,OSTC,PGM3,RPN1,SEC24D,SEC31A,ST6GALNAC4,SYVN1,UAP1
1	regulation of protein metabolic process	56	0.0285	AARS,ALK,ANG,ATF3,ATXN3,BANP,C1QTNF1,CALR,CD36,CTNNB1,CTR9,DDIT4,DERL2,DNAJC10,DUSP6,EBI3,EIF2AK3,FAM129A,FPR1,GABARAPL2,GCLC,GDF15,GPR183,HMGB1,HSP90B1,HSPA5,HSPH1,HTRA2,IER3,IRAK2,KLF4,MPV17L2,NFKBIA,PELI1,PPP1CB,PPP1R15B,PSMC6,RAP1B,RGS2,SELT,SERPINE1,SPOCD1,SUMO1,TAF9,TLR1,TNFRSF4,TNFSF10,TOB1,TOPORS,TRIB1,TRIB3,TRIM5,UBXN1,USP18,WFS1,ZFAND2A
4	cellular response to DNA damage stimulus	23	0.0289	ATMIN,ATXN3,BATF,BCL2A1,DDIT3,DDIT4,DTX3L,DYRK2,EEPD1,FANCE,HMGB1,HTRA2,IER3,LIG4,MSH2,NPLOC4,PSMC6,RAD9A,SUMO1,TAF9,TDP2,UBE2T,UFD1L
4	gamma-aminobutyric acid catabolic process	2	0.0289	ABAT,ALDH5A1
4	beta-alanine biosynthetic process	2	0.0289	DPYD,UPB1
2	positive regulation of B cell proliferation	5	0.0289	ADA,CD38,GPR183,PELI1,TNFRSF4
2	positive regulation of lymphocyte proliferation	8	0.0289	ADA,CD276,CD38,EBI3,GPR183,PELI1,PNP,TNFRSF4
4	regulation of transcription from RNA polymerase II promoter in response to arsenic-containing substance	2	0.0289	ATF3,ATF4
2	cellular response to molecule of bacterial origin	9	0.0289	ANKRD1,CD36,CTR9,IRAK2,NFKBIA,PDE4B,SERPINE1,TLR1,ZC3H12A
1	regulation of ATF6-mediated unfolded protein response	2	0.0289	HSPA5,WFS1
2	immune system process	48	0.0292	APP,ATP1B1,BATF,CALR,CAPZA1,CCL23,CCL4L2,CD164,CD276,CD38,CYP27B1,DDIT4,DUSP6,GPR183,HMGB1,HSP90B1,ID2,IRAK2,KLF4,LIG4,LY75,MATK,MSH2,MT1G,PDE4B,PELI1,PGM3,PIK3AP1,PNP,PRKCSH,PSMC6,RNF19B,ROCK1,SEC24D,SEC31A,SLAMF1,SLC26A6,SLC3A2,SLC7A5,SLFN11,SUMO1,TANK,TMEM91,TNFRSF4,TNFSF10,TRIB3,TRIM5,USP18
4	single-organism catabolic process	27	0.0292	ABAT,ALDH1L2,ALDH5A1,CISD2,CYP27B1,DERL2,DNAJB9,DNAJC10,DPYD,FABP5,GABARAPL2,GPT2,HERPUD1,HSP90B1,HSPA5,NPLOC4,PDE4B,PNP,PPP1CB,PSMC6,SDF2L1,SEL1L,SYVN1,UFD1L,UPB1,WFS1,WIPI1
4	small molecule biosynthetic process	16	0.0295	ABAT,ADI1,ALOX15B,ASNS,CYP27B1,CYP51A1,DPYD,ELOVL6,FABP5,GPT2,MTHFD2,PNP,PSAT1,PTGES,UGDH,UPB1
1	negative regulation of protein metabolic process	30	0.0295	ANG,APP,ATF3,BANP,CALR,CD276,CTNNB1,DDIT4,DERL2,DNAJC10,DUSP6,EIF2AK3,FAM129A,GABARAPL2,GCLC,HERPUD1,HRSP12,HSPH1,KLF4,PPP1R15B,PSMC6,RGS2,SERPINE1,SPOCD1,TAF9,TNFSF10,TOB1,TRIB1,TRIB3,UBXN1
4	anion transmembrane transport	13	0.0302	ASNA1,CD36,CLIC2,CTNS,GABRE,SLC1A4,SLC1A5,SLC22A16,SLC26A6,SLC35B1,SLC3A2,SLC7A1,SLC7A5
3	negative regulation of apoptotic signaling pathway	11	0.0304	BCL2A1,CTNNB1,GCLC,HERPUD1,HSPH1,HTRA2,IER3,SERPINE1,SYVN1,TAF9,WFS1
2	response to lipopolysaccharide	13	0.0315	ACP5,ANKRD1,CD36,CTR9,CYP27B1,IRAK2,NFKBIA,PDE4B,PELI1,PTGES,SERPINE1,TRIB1,ZC3H12A
3	regulation of neuron death	12	0.0315	AARS,ATF4,CTNNB1,DDIT3,DDIT4,GCLC,HTRA2,HYOU1,LIG4,MSH2,ROCK1,WFS1
4	single-organism intracellular transport	32	0.0315	APP,ATP1B1,ATP2A2,CALR,DDIT3,DERL2,GABARAPL2,HERPUD1,HSP90B1,KIAA1033,KIAA1279,KPNA2,NFKBIA,NPLOC4,RPLP1,RPN1,RTP4,SEC11C,SEC24D,SEC31A,SEL1L,SPCS2,SPCS3,SRSF11,SRSF3,SYVN1,TOB1,UFD1L,VPS26A,WFS1,WIPI1,XCR1
1	protein maturation by protein folding	3	0.032	CALR,ERO1LB,WFS1
3	negative regulation of apoptotic process	26	0.032	AARS,ADA,ASNS,BCL2A1,CAST,CD38,CTNNB1,EIF2AK3,HSP90B1,HSPA5,HSPH1,HTRA2,KLF4,LIG4,MERTK,MSH2,NFKBIA,PIM2,PSMC6,ROCK1,SERPINE1,SYVN1,TAF9,TNFSF10,TOPORS,WFS1
1	glycoprotein biosynthetic process	15	0.0333	ALG13,B3GNT5,CALR,EXT1,GFPT1,GMPPB,MLEC,OSTC,PGM3,RPN1,SEC24D,SEC31A,ST6GALNAC4,SYVN1,UAP1
1	regulation of proteasomal ubiquitin-dependent protein catabolic process	9	0.0333	ATXN3,GCLC,HERPUD1,SUMO1,TAF9,TRIB1,TRIB3,UBXN1,ZFAND2A
4	positive regulation of macromolecule metabolic process	59	0.0351	ALK,ANG,ANKRD1,APP,ATF3,ATMIN,ATXN3,BANP,BATF,CALR,CCPG1,CD276,CD36,CD38,CTNNB1,CTR9,DDIT3,DDX17,DYRK2,EBI3,FAM129A,FPR1,FZD2,GCLC,GDF15,GNL3,GPR183,HERPUD1,HMGB1,HSPH1,HTRA2,ID2,IER3,IRAK2,KLF4,MAF,MED21,MPV17L2,NFKBIA,PELI1,PIM2,PSMC6,RAP1B,SERPINE1,SERTAD3,SUMO1,TAF9,TLR1,TNFSF10,TOB1,TOPORS,TRAF1,TRIB1,TRIB3,TRIM5,VSX2,WFS1,ZC3H12A,ZFAND2A
4	positive regulation of cellular metabolic process	62	0.0357	ALK,ANG,ANKRD1,APP,ATF3,ATMIN,ATP1B1,ATXN3,BANP,BATF,C1QTNF1,CALR,CCPG1,CD276,CD36,CD38,CTNNB1,CTR9,DDIT3,DDX17,DYRK2,EBI3,FAM129A,FPR1,FZD2,GCLC,GDF15,GNL3,GPR183,HERPUD1,HMGB1,HSPH1,HTRA2,ID2,IRAK2,KLF4,LARP1B,MAF,MED21,MPV17L2,NFKBIA,PELI1,PIM2,PSMC6,RAP1B,SERPINE1,SERTAD3,SLC1A4,SUMO1,TAF9,TLR1,TMEM39A,TNFSF10,TOB1,TOPORS,TRAF1,TRIB1,TRIB3,TRIM5,VSX2,ZC3H12A,ZFAND2A
4	positive regulation of metabolic process	73	0.0364	ALK,ANG,ANKRD1,APP,ARHGEF10L,ATF3,ATMIN,ATP1B1,ATXN3,BANP,BATF,CALR,CCPG1,CD276,CD36,CD38,CDC42EP3,CTNNB1,CTR9,CYP27B1,DDIT3,DDX17,DNAJC10,DYRK2,EBI3,EVI5L,FAM129A,FPR1,FZD2,GABARAPL2,GCLC,GDF15,GNL3,GPR183,HERPUD1,HMGB1,HSPH1,HTRA2,ID2,IER3,IRAK2,KLF4,LARP1B,MAF,MED21,MPV17L2,MSH2,NFKBIA,PELI1,PIGA,PIM2,PSMC6,RAP1B,RCC1,RGS2,SERPINE1,SERTAD3,SLC1A4,SUMO1,TAF9,TLR1,TMEM39A,TNFSF10,TOB1,TOPORS,TRAF1,TRIB1,TRIB3,TRIM5,VSX2,WFS1,ZC3H12A,ZFAND2A

4	protein localization	42	0.0384	ASNA1,ATP1B1,BANP,CALR,CD36,CTNNB1,DERL2,EIF2AK3,FKBP4,GABARAPL2,HERPUD1,HSP90B1,HSPA5,KIAA1033,LIN7C,NFKBIA,NPLOC4,PDIA4,PNP,RNF19B,RPLP1,RPN1,RTP4,SAR1A,SEC11C,SEC24D,SEC31A,SEL1L,SELT,SIL1,SPCS2,SPCS3,SYVN1,TCP1,TMED7,TOB1,TOPORS,TRAM2,UFD1L,VPS26A,WFS1,WIPI1
3	negative regulation of cell death	27	0.0398	AARS,ADA,ASNS,ATF4,BCL2A1,CAST,CD38,CTNNB1,EIF2AK3,HSP90B1,HSPA5,HSPH1,HTRA2,KLF4,LIG4,MERTK,MSH2,NFKBIA,PIM2,PSMC6,ROCK1,SERPINE1,SYVN1,TAF9,TNFSF10,TOPORS,WFS1
4	primary metabolic process	148	0.0405	ADI1,AGPAT9,ALDH1L2,ALK,AMY2B,ANG,ANKRD1,ANP32A,ASNA1,ASNS,ATF7IP2,ATMIN,B3GNT5,BANP,BCAS2,C12orf65,C1D,CALR,CARS,CD36,CHST2,COMMD8,CTNNB1,CTNSCTR9,CYP27B1,CYP51A1,DDIT3,DDIT4,DDX17,DERL2,DNAJA4,DNAJB5,DNAJB9,DNAJC10,DPYD,DTX3L,DUSP6,DYRK2,EEF1B2,EEPD1,ELOVL6,EXT1,FANCE,FICD,FKBP11,FKBP14,FKBP2,FKBP4,FKBP5,G3BP1,GADD45GIP1,GFPT1,GMPPB,GPT2,HABP4,HMGB1,HRSP12,HSP90B1,HSPA5,HSPH1,HYOU1,ID2,IRAK2,KLF4,KPNA2,LIG4,LMO4,LYPLA1,MATK,MBNL2,MERTK,MLEC,MSH2,MTMR4,N4BP2,NDUFA5,NFXL1,NPLOC4,OSTC,PC,PDE4B,PDIA4,PDIA5,PDIA6,PELI1,PFDN2,PGM3,PIGA,PIM2,PNP,PPIG,PPIL1,PPP1CB,PRPF4B,PSAT1,PSMC6,PTGES,PTMA,RAD9A,RNASE4,RNF19B,ROCK1,RPLP1,RPN1,SDF2L1,SEC24D,SEC31A,SEL1L,SELT,SERPINE1,SERTAD3,SIL1,SLC3A2,SLC7A5,SPOCD1,SRSF11,SRSF3,ST6GALNAC4,SUMO1,SYVN1,TAF9,TARS,TCP1,TDP2,TNFSF10,TOPORS,TRIB1,TRIM5,TSN,TUBA3D,TUBB3,UAP1,UBA5,UBE2T,UBXN1,UFD1L,UGDH,UPB1,USP18,VSX2,VWF,WFS1,WIPI1,WTAP,ZC3H12A,ZNF395,ZNF768
3	regulation of intrinsic apoptotic signaling pathway	9	0.0405	BCL2A1,DDIT3,HERPUD1,HSPH1,HTRA2,RAD9A,SYVN1,TAF9,WFS1
4	regulation of sequence-specific DNA binding transcription factor activity	15	0.0409	ALK,DDIT3,FZD2,ID2,IRAK2,KLF4,NFKBIA,PELI1,SUMO1,TNFRSF4,TRAF1,TRIB1,TRIM5,WFS1,ZC3H12A
1	intracellular protein transport	23	0.0485	ATP1B1,CALR,DERL2,HERPUD1,HSP90B1,KPNA2,NFKBIA,NPLOC4,RPLP1,RPN1,RTP4,SAR1A,SEC11C,SEC24D,SEL1L,SIL1,SPCS2,SPCS3,SYVN1,TOB1,UFD1L,VPS26A,WFS1
4	cell communication	97	0.0486	AARS,ABAT,ALDH5A1,ALK,ANG,ANP32A,APP,ASNA1,ASNS,ATP1B1,ATP2A2,ATXN3,BATF,BCL2A1,CALR,CCL23,CD164,CD36,CD38,CDC42EP3,CISD2,CLIC2,CTNNB1,CTR9,DDIT4,DERL2,DNAJB11,DNAJB9,DNAJC10,DUSP6,DYRK2,EBI3,EVL,EXT1,FKBP14,FKBP4,FPR1,FPR3,G3BP1,GABARAPL2,GABRE,GDF15,GFPT1,GNB4,GPR132,GPR183,HERPUD1,HSP90B1,HSPA5,HTRA2,HYOU1,IER3,IRAK2,KCNK6,KLF4,KPNA2,LIN7C,MATK,MIS12,MSH2,MTMR4,PDE4B,PDIA5,PDIA6,PIK3AP1,PPP1CB,PPP1R15B,PPP2CB,PRKCSH,PSMC6,PTGES,ROCK1,RTKN,SEC31A,SEL1L,SELK,SELT,SEMA3A,SERPINE1,SLAMF1,SLC1A4,SLC26A6,SRSF3,SUMO1,SYVN1,TDP2,TNFRSF4,TNFSF10,TOB1,TRAF1,TRIB1,TRIB3,TRIM5,USP18,WFS1,WIPI1,XCR1
4	carboxylic acid biosynthetic process	12	0.0486	ABAT,ADI1,ALOX15B,ASNS,DPYD,ELOVL6,GPT2,MTHFD2,PSAT1,PTGES,UGDH,UPB1
3	negative regulation of intrinsic apoptotic signaling pathway	7	0.0486	BCL2A1,HERPUD1,HSPH1,HTRA2,SYVN1,TAF9,WFS1
4	regulation of localization	52	0.0489	ABAT,ADA,ALOX15B,ANG,ANKRD1,ATF4,ATP1B1,ATP2A2,BORA,C1QTNF1,CALR,CD276,CD36,CD38,CLIC2,CTNNB1,DDIT3,DERL2,DNAAF1,DYRK2,EIF2AK3,EVL,HMGB1,HSPA5,HSPH1,HTRA2,IER3,KCNK6,KLF4,LYPLA1,MERTK,NFKBIA,PDE4B,RAP1B,ROCK1,SAR1A,SEC11C,SERPINE1,SLAMF1,SLC17A9,SLC26A6,SPCS2,SPCS3,SUMO1,TLR1,TNFRSF4,TNFRSF9,TRIB1,TRIB3,WFS1,XCR1,ZC3H12A
4	positive regulation of macromolecule biosynthetic process	40	0.0494	ALK,ANKRD1,APP,ATF3,ATMIN,BANP,BATF,CALR,CCPG1,CD276,CD38,CTNNB1,CTR9,DDIT3,DDX17,DYRK2,EBI3,FAM129A,FZD2,GNL3,HMGB1,HSPA5,HSPH1,ID2,IRAK2,KLF4,MAF,MED21,MPV17L2,NFKBIA,PIM2,PSMC6,SERPINE1,SERTAD3,TAF9,TLR1,TOPORS,TRAF1,TRIM5,VSX2

Pathway association denoted by colour as follows: UPR only (Blue), Inflammation only (Green), Cell survival (Red), UPR and inflammation (Purple), General cell process (Grey)
