# Supplementary material for: Pathogen‐induced inflammation is attenuated by the iminosugar MON‐DNJ via modulation of the unfolded protein response
Source: Immunology. 2021 Aug 1;164(3):587–601. doi: 10.1111/imm.13393 (PMC8517592; doi:10.1111/imm.13393)
Supplement: Supplementary file 7 — Table S4 [file IMM-164-587-s001.pdf]

Supplemental Table 4A. UPR-associated STRING-DB networks responding to M*O*N-DNJ

Pathway	Count	FDR	Targets
response to endoplasmic reticulum stress	35	1.43E-22	AARS,ASNA1,ASNS,ATF3,ATF4,ATP2A2,CALR,DERL2,DNAJB11,DNAJB9,DNAJC10,EIF2AK3,FAM129A,FKBP14,GFPT1,HERPUD1,HSP90B1,HSPA5,HYOU1,NPLOC4,PDIA4,PDIA5,PDIA6,PPP1R15B,PPP2CB,PSMC6,SDF2L1,SEC31A,SELK,SYVN1,TRIB3,UBA5,UFD1L,WFS1,WIPI1
response to topologically incorrect protein	29	7.93E-19	AARS,ASNA1,ASNS,ATF3,ATF4,ATXN3,CALR,DDIT3,DERL2,DNAJB11,DNAJB5,DNAJB9,EIF2AK3,FKBP14,GFPT1,HERPUD1,HSP90B1,HSPA5,HSPH1,HYOU1,MANF,PDIA5,PDIA6,SDF2L1,SEC31A,SYVN1,UFD1L,WFS1,WIPI1
cellular response to topologically incorrect protein	26	7.93E-19	AARS,ASNA1,ASNS,ATF3,ATF4,ATXN3,CALR,DDIT3,DERL2,DNAJB11,DNAJB9,EIF2AK3,FKBP14,GFPT1,HERPUD1,HSP90B1,HSPA5,HYOU1,PDIA5,PDIA6,SDF2L1,SEC31A,SYVN1,UFD1L,WFS1,WIPI1
response to unfolded protein	26	2.12E-16	AARS,ASNA1,ASNS,ATF3,ATF4,CALR,DDIT3,DERL2,DNAJB11,DNAJB5,DNAJB9,EIF2AK3,FKBP14,GFPT1,HERPUD1,HSP90B1,HSPA5,HSPH1,HYOU1,MANF,PDIA5,PDIA6,SEC31A,SYVN1,WFS1,WIPI1
endoplasmic reticulum unfolded protein response	23	2.12E-16	AARS,ASNA1,ASNS,ATF3,ATF4,CALR,DDIT3,DERL2,DNAJB11,DNAJB9,EIF2AK3,FKBP14,GFPT1,HERPUD1,HSP90B1,HSPA5,HYOU1,PDIA5,PDIA6,SEC31A,SYVN1,WFS1,WIPI1
cellular response to unfolded protein	23	2.78E-16	AARS,ASNA1,ASNS,ATF3,ATF4,CALR,DDIT3,DERL2,DNAJB11,DNAJB9,EIF2AK3,FKBP14,GFPT1,HERPUD1,HSP90B1,HSPA5,HYOU1,PDIA5,PDIA6,SEC31A,SYVN1,WFS1,WIPI1
protein folding	27	2.12E-14	AARS,CALR,DNAJA4,DNAJB11,DNAJB5,DNAJC10,FKBP11,FKBP14,FKBP2,FKBP4,FKBP5,HSP90B1,HSPA5,HSPH1,MLEC,PDIA4,PDIA5,PDIA6,PFDN2,PPIG,PPIL1,PRKCSH,SIL1,TCP1,TUBA3D,TUBB3,WFS1
cellular response to stress	71	4.02E-14	AARS,ANKRD1,ASNA1,ASNS,ATF4,ATMIN,ATP1B1,ATP2A2,ATXN3,BATF,BCL2A1,CALR,CD36,CISD2,DDIT3,DDIT4,DERL2,DNAJB11,DNAJB9,DNAJC10,DTX3L,DUSP6,DYRK2,EEPD1,EIF2AK3,FAM129A,FANCE,FKBP14,FKBP4,GABARAPL2,GFPT1,HERPUD1,HMGB1,HSP90B1,HSPA5,HSPH1,HTRA2,HYOU1,ID2,IER3,IRAK2,KLF4,LARP1B,LIG4,MSH2,NFKBIA,NPLOC4,PDIA4,PDIA5,PDIA6,PPP1CB,PPP1R15B,PPP2CB,PSMC6,RAD9A,SDF2L1,SEC31A,SELK,SLC1A4,SUMO1,SYVN1,TAF9,TDP2,TMEM39A,TRIB1,TRIB3,UBA5,UBE2T,UFD1L,WFS1,WIPI1
response to stress	105	4.00E-12	AARS,ABAT,ACP5,ANG,ANKRD1,ASNA1,ASNS,ATF4,ATMIN,ATP2A2,ATXN3,BATF,BCL2A1,CALR,CAPZA1,CCL23,CCL4L2,CD163,CD38,CHST2,CISD2,CTNNB1,CYP27B1,DDIT3,DDIT4,DERL2,DNAJA4,DNAJB11,DNAJB5,DNAJB9,DNAJC10,DTX3L,DUSP6,DYRK2,EEPD1,EIF2AK3,FABP5,FAM129A,FANCE,FKBP14,FKBP4,GABARAPL2,GFPT1,HABP4,HERPUD1,HMGB1,HSP90B1,HSPA5,HSPH1,HYOU1,ID2,IER3,IRAK2,KLF4,LARP1B,LIG4,LY75,MANF,MATK,METRNL,MSH2,NFKBIA,NPLOC4,PDIA4,PDIA5,PDIA6,PELI1,PPP1CB,PPP1R15B,PPP2CB,PRKCSH,PSMC6,PTGES,RAD9A,RAP1B,RNF19B,SDF2L1,SEC31A,SELK,SERPINE1,SLC1A4,SLC26A6,SLC3A2,SLC7A5,SLFN11,SP140,SUMO1,SYVN1,TAF9,TANK,TDP2,TLR1,TMEM39A,TNFRSF4,TRIB1,TRIB3,TRIM5,UBA5,UBE2T,UFD1L,USP18,VWF,WFS1,WIPI1,XCR1
IRE1-mediated unfolded protein response	13	7.12E-09	ASNA1,DNAJB11,DNAJB9,FKBP14,GFPT1,HSPA5,HYOU1,PDIA5,PDIA6,SEC31A,SYVN1,WFS1,WIPI1
ERAD pathway	13	1.29E-08	DERL2,DNAJB9,DNAJC10,HERPUD1,HSP90B1,HSPA5,NPLOC4,PSMC6,SDF2L1,SEL1L,SYVN1,UFD1L,WFS1
ER-associated ubiquitin-dependent protein catabolic process	12	8.06E-08	DERL2,DNAJB9,DNAJC10,HSP90B1,HSPA5,NPLOC4,PSMC6,SDF2L1,SEL1L,SYVN1,UFD1L,WFS1
PERK-mediated unfolded protein response	7	1.71E-07	ASNS,ATF3,ATF4,DDIT3,EIF2AK3,HERPUD1,HSPA5
ER-nucleus signaling pathway	9	7.66E-07	ASNS,ATF3,ATF4,ATP2A2,CALR,HERPUD1,HSP90B1,PPP1R15B,WFS1
retrograde protein transport, ER to cytosol	7	1.10E-06	DERL2,HERPUD1,HSP90B1,NPLOC4,SEL1L,SYVN1,UFD1L
cellular protein metabolic process	90	1.10E-06	ALG13,ALK,APP,ASNA1,ASNS,ATF3,ATF4,ATXN3,B3GNT5,C12orf65,CALR,CARS,CTR9,DDIT3,DERL2,DNAJA4,DNAJB5,DNAJB9,DNAJC10,DTX3L,DUSP6,DYRK2,EEF1B2,EXT1,FICD,FKBP11,FKBP14,FKBP2,FKBP4,FKBP5,GADD45GIP1,GFPT1,GMPPB,HSP90B1,HSPA5,HSPH1,HTRA2,HYOU1,IRAK2,LYPLA1,MATK,MED21,MERTK,MLEC,MTMR4,NPLOC4,OSTC,PDIA4,PDIA5,PDIA6,PELI1,PFDN2,PGM3,PIGA,PIM2,PPIG,PPIL1,PPP1CB,PRPF4B,PSMC6,RNF19B,ROCK1,RPLP1,RPN1,SDF2L1,SEC24D,SEC31A,SEL1L,SELT,SIL1,ST6GALNAC4,SUMO1,SYVN1,TAF9,TARS,TCP1,TOPORS,TRIB1,TRIB3,TRIM5,TUBA3D,TUBB3,UAP1,UBA5,UBE2T,UBXN1,UFD1L,USP18,WFS1,WIPI1
protein N-linked glycosylation via asparagine	14	6.66E-06	ALG13,CALR,GFPT1,GMPPB,MLEC,OSTC,PGM3,PRKCSH,RPN1,SEC24D,SEC31A,ST6GALNAC4,SYVN1,UAP1
protein N-linked glycosylation	13	8.95E-05	ALG13,CALR,GFPT1,GMPPB,MLEC,OSTC,PGM3,RPN1,SEC24D,SEC31A,ST6GALNAC4,SYVN1,UAP1
ER overload response	5	0.000117	DDIT3,EIF2AK3,HSPA5,PPP1R15B,WFS1
chaperone-mediated protein folding	8	0.000155	CALR,FKBP11,FKBP14,FKBP2,FKBP4,FKBP5,HSPH1,PDIA4
proteasome-mediated ubiquitin-dependent protein catabolic process	18	0.000432	ATXN3,DDIT3,DERL2,DNAJB9,DNAJC10,HSP90B1,HSPA5,NPLOC4,PPP2CB,PSMC6,SDF2L1,SEL1L,SYVN1,TOPORS,UBXN1,UFD1L,USP18,WFS1
protein metabolic process	91	0.000448	ALG13,ALK,APP,ASNA1,ASNS,ATF3,ATF4,B3GNT5,C12orf65,CALR,CARS,CTR9,DDIT3,DERL2,DNAJA4,DNAJB5,DNAJB9,DNAJC10,DTX3L,DUSP6,DYRK2,EEF1B2,EXT1,FICD,FKBP11,FKBP14,FKBP2,FKBP4,FKBP5,GADD45GIP1,GFPT1,GMPPB,HSP90B1,HSPA5,HSPH1,HTRA2,HYOU1,IRAK2,LYPLA1,MATK,MED21,MERTK,MLEC,MTMR4,NPLOC4,OSTC,PDIA4,PDIA5,PDIA6,PELI1,PFDN2,PGM3,PIGA,PIM2,PPIG,PPIL1,PPP1CB,PRPF4B,PSMC6,RNF19B,ROCK1,RPLP1,RPN1,SDF2L1,SEC24D,SEC31A,SEL1L,SELT,SIL1,ST6GALNAC4,SUMO1,SYVN1,TAF9,TARS,TCP1,TNFSF10,TOPORS,TRIB1,TRIB3,TRIM5,TUBA3D,TUBB3,UAP1,UBA5,UBE2T,UBXN1,UFD1L,USP18,VWF,WFS1,WIPI1
protein peptidyl-prolyl isomerization	7	0.000938	FKBP11,FKBP14,FKBP2,FKBP4,FKBP5,PPIG,PPIL1

transport	80	0.001	ABAT,ADA,ALDH5A1,ANP32A,APP,ASNA1,ATP1B1,ATP2A2,CALR,CD163,CD36,CLIC2,CTNNB1,CTNS,CYB561,CYP27B1,DDIT3,DERL2,DYNC1LI1,FABP5,FKBP4,G3BP1,GABARAPL2,HABP4,HERPUD1,HIAT1,HSP90B1,HSPH1,HYOU1,KCNK6,KIAA1033,KIAA1279,LIN7C,LY75,MERTK,MFSD2A,NFKBIA,NPLOC4,PDIA4,PNP,RNF19B,RPLP1,RPN1,RTP4,SAR1A,SEC11C,SEC31A,SEL1L,SELK,SELT,SERPINE1,SIL1,SLC10A7,SLC17A9,SLC1A4,SLC1A5,SLC22A16,SLC26A6,SLC35B1,SLC39A14,SLC3A2,SLC7A1,SLC7A5,SPCS2,SPCS3,SRSF11,SRSF3,SYVN1,TCP1,TMED7,TMEM38B,TOB1,TRAM2,UFD1L,UQCRH,VPS26A,VWF,WFS1,WIPI1,XCR1
nucleotide-sugar biosynthetic process	5	0.00117	GFPT1,GMPPB,PGM3,UAP1,UGDH
protein folding in endoplasmic reticulum	4	0.00129	CALR,DNAJC10,HSP90B1,HSPA5
cell redox homeostasis	8	0.00175	DDIT3,DNAJC10,ERO1LB,GCLC,PDIA4,PDIA5,PDIA6,SELT
ATF6-mediated unfolded protein response	4	0.00214	CALR,DDIT3,HSP90B1,HSPA5
negative regulation of sequence-specific DNA binding transcription factor activity	11	0.00261	DDIT3,ID2,IRAK2,KLF4,NFKBIA,PELI1,SUMO1,TNFRSF4,TRIB1,WFS1,ZC3H12A
intrinsic apoptotic signaling pathway in response to endoplasmic reticulum stress	6	0.00261	ATF4,DDIT3,DNAJC10,EIF2AK3,SELK,TRIB3
peptidyl-amino acid modification	30	0.00292	ALG13,ALK,CALR,CTR9,DYRK2,EIF2AK3,FKBP11,FKBP14,FKBP2,FKBP4,FKBP5,GFPT1,GMPPB,MATK,MERTK,MLEC,OSTC,PGM3,PPIG,PPIL1,PRKCSH,RPN1,SEC24D,SEC31A,ST6GALNAC4,SUMO1,SYVN1,TAF9,TOPORS,UAP1
cellular amino acid biosynthetic process	8	0.00499	ABAT,ADI1,ASNS,DPYD,GPT2,MTHFD2,PSAT1,UPB1
positive regulation of transcription from RNA polymerase II promoter in response to endoplasmic reticulum stress	4	0.00605	ATF3,ATF4,DDIT3,HSPA5
single-organism carbohydrate metabolic process	23	0.00637	ALDH5A1,ATF3,ATF4,B3GNT5,CALR,EXT1,FABP5,GCLC,GFPT1,GMPPB,GOT1,MLEC,OSTC,PC,PGM3,PPP1CB,RPN1,SEC24D,SEC31A,ST6GALNAC4,SYVN1,UAP1,UGDH
positive regulation of proteasomal protein catabolic process	8	0.00679	ATXN3,GCLC,HERPUD1,PSMC6,SUMO1,TRIB1,TRIB3,ZFAND2A
ubiquitin-dependent protein catabolic process	19	0.00715	ATXN3,DDIT3,DERL2,DNAJB9,DNAJC10,HERPUD1,HSP90B1,HSPA5,NPLOC4,PPP2CB,PSMC6,SDF2L1,SEL1L,SYVN1,TOPORS,UBXN1,UFD1L,USP18,WFS1
UDP-N-acetylglucosamine biosynthetic process	3	0.00768	GFPT1,PGM3,UAP1
protein glycosylation	15	0.00911	ALG13,B3GNT5,CALR,EXT1,GFPT1,GMPPB,MLEC,OSTC,PGM3,RPN1,SEC24D,SEC31A,ST6GALNAC4,SYVN1,UAP1
regulation of binding	14	0.0099	APP,CCL23,CLIC2,DDIT3,EIF2AK3,HABP4,HERPUD1,HMGB1,HSPA5,ID2,NFKBIA,ROCK1,SUMO1,TRIB3
regulation of endoplasmic reticulum stress-induced intrinsic apoptotic signaling pathway	5	0.0105	DDIT3,HERPUD1,HYOU1,SYVN1,WFS1
cellular protein catabolic process	20	0.0131	ATXN3,DDIT3,DERL2,DNAJB9,DNAJC10,HERPUD1,HSP90B1,HSPA5,HTRA2,NPLOC4,PPP2CB,PSMC6,SDF2L1,SEL1L,SYVN1,TOPORS,UBXN1,UFD1L,USP18,WFS1
positive regulation of proteasomal ubiquitin-dependent protein catabolic process	7	0.0163	ATXN3,GCLC,HERPUD1,SUMO1,TRIB1,TRIB3,ZFAND2A
negative regulation of endoplasmic reticulum stress-induced intrinsic apoptotic signaling pathway	4	0.0163	HERPUD1,HYOU1,SYVN1,WFS1
negative regulation of response to endoplasmic reticulum stress	5	0.0163	DERL2,HERPUD1,HYOU1,PPP1R15B,SYVN1
protein transport	35	0.0171	ATP1B1,CALR,CD36,DERL2,EIF2AK3,GABARAPL2,HERPUD1,HSP90B1,KIAA1033,LIN7C,NFKBIA,NPLOC4,PDIA4,PNP,RNF19B,RPLP1,RPN1,RTP4,SAR1A,SEC11C,SEC24D,SEC31A,SEL1L,SELT,SIL1,SPCS2,SPCS3,SYVN1,TCP1,TMED7,TOB1,TRAM2,UFD1L,VPS26A,WFS1
positive regulation of protein metabolic process	38	0.0192	ALK,ANG,APP,ATXN3,C1QTNF1,CD276,CD36,CTNNB1,CTR9,EBI3,EIF2AK3,FAM129A,FPR1,GCLC,GDF15,GPR183,HERPUD1,HMGB1,HSPA5,HSPH1,HTRA2,IER3,IRAK2,KLF4,MPV17L2,NFKBIA,PELI1,PSMC6,RAP1B,SUMO1,TLR1,TNFSF10,TOPORS,TRIB1,TRIB3,TRIM5,WFS1,ZFAND2A
endoplasmic reticulum calcium ion homeostasis	4	0.0223	APP,ATP2A2,HERPUD1,WFS1
regulation of proteasomal protein catabolic process	12	0.0246	ATXN3,GABARAPL2,GCLC,HERPUD1,PSMC6,SUMO1,TAF9,TRIB1,TRIB3,UBXN1,USP18,ZFAND2A
post-translational protein modification	15	0.0256	ALG13,B3GNT5,CALR,GFPT1,GMPPB,MLEC,PGM3,PIGA,PRKCSH,RPN1,SEC24D,SEC31A,ST6GALNAC4,SUMO1,UAP1
protein catabolic process	20	0.0281	ATXN3,DDIT3,DERL2,DNAJB9,DNAJC10,HSP90B1,HSPA5,HTRA2,LYPLA1,NPLOC4,PPP2CB,PSMC6,SDF2L1,SEL1L,SYVN1,TOPORS,UBXN1,UFD1L,USP18,WFS1
glycosylation	14	0.0281	B3GNT5,CALR,EXT1,GFPT1,GMPPB,MLEC,OSTC,PGM3,RPN1,SEC24D,SEC31A,ST6GALNAC4,SYVN1,UAP1

regulation of protein metabolic process	56	0.0285	AARS,ALK,ANG,ATF3,ATXN3,BANP,C1QTNF1,CALR,CD36,CTNNB1,CTR9,DDIT4,DERL2,DNAJC10,DUSP6,EBI3,EIF2AK3,FAM129A,FPR1,GABARAPL2,GCLC,GDF15,GPR183,HMGB1,HSP90B1,HSPA5,HSPH1,HTRA2,IER3,IRAK2,KLF4,MPV17L2,NFKBIA,PELI1,PPP1CB,PPP1R15B,PSMC6,RAP1B,RGS2,SELT,SERPINE1,SPOCD1,SUMO1,TAF9,TLR1,TNFRSF4,TNFSF10,TOB1,TOPORS,TRIB1,TRIB3,TRIM5,UBXN1,USP18,WFS1,ZFAND2A
regulation of ATF6-mediated unfolded protein response	2	0.0289	HSPA5,WFS1
negative regulation of protein metabolic process	30	0.0295	ANG,APP,ATF3,BANP,CALR,CD276,CTNNB1,DDIT4,DERL2,DNAJC10,DUSP6,EIF2AK3,FAM129A,GABARAPL2,GCLC,HERPUD1,HRSP12,HSPH1,KLF4,PPP1R15B,PSMC6,RGS2,SERPINE1,SPOCD1,TAF9,TNFSF10,TOB1,TRIB1,TRIB3,UBXN1
protein maturation by protein folding	3	0.032	CALR,ERO1LB,WFS1
glycoprotein biosynthetic process	15	0.0333	ALG13,B3GNT5,CALR,EXT1,GFPT1,GMPPB,MLEC,OSTC,PGM3,RPN1,SEC24D,SEC31A,ST6GALNAC4,SYVN1,UAP1
regulation of proteasomal ubiquitin-dependent protein catabolic process	9	0.0333	ATXN3,GCLC,HERPUD1,SUMO1,TAF9,TRIB1,TRIB3,UBXN1,ZFAND2A
intracellular protein transport	23	0.0485	ATP1B1,CALR,DERL2,HERPUD1,HSP90B1,KPNA2,NFKBIA,NPLOC4,RPLP1,RPN1,RTP4,SAR1A,SEC11C,SEC24D,SEL1L,SIL1,SPCS2,SPCS3,SYVN1,TOB1,UFD1L,VPS26A,WFS1

Supplemental Table 4B. Inflammation-associated STRING-DB networks responding to M*O*N-DNJ

Pathway	Count	FDR	Targets
toll-like receptor signaling pathway	9	0.0168	CD36,DUSP6,HSP90B1,IRAK2,NFKBIA,PELI1,PIK3AP1,TANK,TLR1
B cell activation involved in immune response	5	0.0179	ADA,BATF,GPR183,LIG4,MSH2
response to molecule of bacterial origin	14	0.0187	ACP5,ANKRD1,CD36,CTR9,CYP27B1,IRAK2,NFKBIA,PDE4B,PELI1,PTGES,SERPINE1,TLR1,TRIB1,ZC3H12A
immune response	36	0.0248	ADA,APP,BATF,CAPZA1,CCL23,CCL4L2,CD164,CD276,CD36,CTNNB1,CYP27B1,DUSP6,EBI3,GPR183,HMGB1,HSP90B1,IRAK2,LIG4,LY75,MATK,MSH2,NFKBIA,PELI1,PNP,PRKCSH,PSMC6,RNF19B,SLC26A6,SUMO1,TANK,TLR1,TNFRSF4,TNFSF10,TRIB3,TRIM5,USP18
positive regulation of B cell proliferation	5	0.0289	ADA,CD38,GPR183,PELI1,TNFRSF4
positive regulation of lymphocyte proliferation	8	0.0289	ADA,CD276,CD38,EBI3,GPR183,PELI1,PNP,TNFRSF4
cellular response to molecule of bacterial origin	9	0.0289	ANKRD1,CD36,CTR9,IRAK2,NFKBIA,PDE4B,SERPINE1,TLR1,ZC3H12A
immune system process	48	0.0292	APP,ATP1B1,BATF,CALR,CAPZA1,CCL23,CCL4L2,CD164,CD276,CD38,CYP27B1,DDIT4,DUSP6,GPR183,HMGB1,HSP90B1,ID2,IRAK2,KLF4,LIG4,LY75,MATK,MSH2,MT1G,PDE4B,PELI1,PGM3,PIK3AP1,PNP,PRKCSH,PSMC6,RNF19B,ROCK1,SEC24D,SEC31A,SLAMF1,SLC26A6,SLC3A2,SLC7A5,SLFN11,SUMO1,TANK,TMEM91,TNFRSF4,TNFSF10,TRIB3,TRIM5,USP18
response to lipopolysaccharide	13	0.0315	ACP5,ANKRD1,CD36,CTR9,CYP27B1,IRAK2,NFKBIA,PDE4B,PELI1,PTGES,SERPINE1,TRIB1,ZC3H12A

Supplemental Table 4C. Cell fate-associated STRING-DB networks responding to MON-DNJ

Pathway	Count	FDR	Targets
intrinsic apoptotic signaling pathway	12	0.00255	ATF4,BCL2A1,DDIT3,DDIT4,DNAJC10,DYRK2,EIF2AK3,HTRA2,IER3,MSH2,SELK,TRIB3
intrinsic apoptotic signaling pathway in response to endoplasmic reticulum stress	6	0.00261	ATF4,DDIT3,DNAJC10,EIF2AK3,SELK,TRIB3
regulation of apoptotic process	41	0.00262	AARS,ADA,ALK,ANKRD1,ASNS,ATF3,ATF4,BCL2A1,BEX2,CALR,CAST,CD38,CTNNB1,DDIT3,DUSP6,GDF15,HMGB1,HSP90B1,HSPA5,HSPH1,HTRA2,KLF4,LIG4,MERTK,MSH2,NFKBIA,PIM2,PPP1CB,PSMC6,RAD9A,ROCK1,SDF2L1,SERPINE1,SYVN1,TAF9,TCP1,TNFRSF4,TNFSF10,TOPORS,TRAF1,WFS1
regulation of cell death	42	0.00471	AARS,ADA,ALK,ANKRD1,ASNS,ATF3,ATF4,BCL2A1,BEX2,CALR,CAST,CD38,CTNNB1,DDIT3,DDIT4,DUSP6,GDF15,HMGB1,HSP90B1,HSPA5,HSPH1,HTRA2,KLF4,LIG4,MERTK,MSH2,NFKBIA,PIM2,PPP1CB,PSMC6,RAD9A,ROCK1,SDF2L1,SERPINE1,SYVN1,TAF9,TCP1,TNFRSF4,TNFSF10,TOPORS,TRAF1,WFS1
programmed cell death	33	0.0101	ALOX15B,APP,ATF4,BCL2A1,BEX2,C1D,CD38,CTNNB1,DDIT3,DDIT4,DNAJC10,DYRK2,GCLC,HERPUD1,HMGB1,HTRA2,KLF4,LIG4,MSH2,NFKBIA,PIM2,PPP2CB,PSMC6,ROCK1,RTKN,SELK,SEMA3A,TNFRSF4,TNFRSF9,TNFSF10,TRAF1,TRIB3,ZC3H12A
regulation of endoplasmic reticulum stress-induced intrinsic apoptotic signaling pathway	5	0.0105	DDIT3,HERPUD1,HYOU1,SYVN1,WFS1
regulation of apoptotic signaling pathway	17	0.0137	ATF3,BCL2A1,CTNNB1,DDIT3,GCLC,HERPUD1,HSPH1,HTRA2,IER3,PPP1CB,RAD9A,SERPINE1,SYVN1,TAF9,TNFSF10,TRAF1,WFS1
positive regulation of proteasomal ubiquitin-dependent protein catabolic process	7	0.0163	ATXN3,GCLC,HERPUD1,SUMO1,TRIB1,TRIB3,ZFAND2A
negative regulation of endoplasmic reticulum stress-induced intrinsic apoptotic signaling pathway	4	0.0163	HERPUD1,HYOU1,SYVN1,WFS1
apoptotic process	32	0.0171	ALOX15B,APP,ATF4,BCL2A1,BEX2,C1D,CD38,CTNNB1,DDIT3,DDIT4,DNAJC10,DYRK2,GCLC,HERPUD1,HMGB1,HTRA2,KLF4,LIG4,MSH2,NFKBIA,PIM2,PPP2CB,PSMC6,ROCK1,RTKN,SELK,SEMA3A,TNFRSF9,TNFSF10,TRAF1,TRIB3,ZC3H12A
negative regulation of neuron death	10	0.0248	AARS,ATF4,CTNNB1,GCLC,HTRA2,HYOU1,LIG4,MSH2,ROCK1,WFS1
negative regulation of apoptotic signaling pathway	11	0.0304	BCL2A1,CTNNB1,GCLC,HERPUD1,HSPH1,HTRA2,IER3,SERPINE1,SYVN1,TAF9,WFS1
regulation of neuron death	12	0.0315	AARS,ATF4,CTNNB1,DDIT3,DDIT4,GCLC,HTRA2,HYOU1,LIG4,MSH2,ROCK1,WFS1
negative regulation of apoptotic process	26	0.032	AARS,ADA,ASNS,BCL2A1,CAST,CD38,CTNNB1,EIF2AK3,HSP90B1,HSPA5,HSPH1,HTRA2,KLF4,LIG4,MERTK,MSH2,NFKBIA,PIM2,PSMC6,ROCK1,SERPINE1,SYVN1,TAF9,TNFSF10,TOPORS,WFS1
negative regulation of cell death	27	0.0398	AARS,ADA,ASNS,ATF4,BCL2A1,CAST,CD38,CTNNB1,EIF2AK3,HSP90B1,HSPA5,HSPH1,HTRA2,KLF4,LIG4,MERTK,MSH2,NFKBIA,PIM2,PSMC6,ROCK1,SERPINE1,SYVN1,TAF9,TNFSF10,TOPORS,WFS1
regulation of intrinsic apoptotic signaling pathway	9	0.0405	BCL2A1,DDIT3,HERPUD1,HSPH1,HTRA2,RAD9A,SYVN1,TAF9,WFS1
negative regulation of intrinsic apoptotic signaling pathway	7	0.0486	BCL2A1,HERPUD1,HSPH1,HTRA2,SYVN1,TAF9,WFS1
