# Supplementary material for: Pathogen‐induced inflammation is attenuated by the iminosugar MON‐DNJ via modulation of the unfolded protein response
Source: Immunology. 2021 Aug 1;164(3):587–601. doi: 10.1111/imm.13393 (PMC8517592; doi:10.1111/imm.13393)
Supplement: Supplementary file 8 — Table S5 [file IMM-164-587-s003.pdf]

Supplemental Table 5. Cluster 4 gene signature members

Gene Symbol	Gene Name
ABAT	4-aminobutyrate aminotransferase
ADI1	Acireductone dioxygenase 1
ALDH5A1	Aldehyde dehydrogenase 5 family, member A1
ALG13	Asparagine-linked glycosylation 13 homolog (S. cerevisiae)
AMY2B	Amylase, alpha 2B (pancreatic)
ANP32A	Acidic (leucine-rich) nuclear phosphoprotein 32 family, member A
ATMIN	ATM interactor
B3GNT5	UDP-GlcNAc:betaGal beta-1,3-N-acetylglucosaminyltransferase 5
BCAS2	Breast carcinoma amplified sequence 2
BCL2A1	BCL2-related protein A1
C1D	C1D nuclear receptor corepressor
C6orf62	Chromosome 6 open reading frame 62
CAPZA1	Capping protein (actin filament) muscle Z-line, alpha 1
CAST	Calpastatin
CBWD1	COBW domain containing 1
CD164	CD164 molecule, sialomucin
COMMD8	COMM domain containing 8
CTNNB1	Catenin (cadherin-associated protein), beta 1, 88kDa
CTR9	Ctr9, Paf1/RNA polymerase II complex component, homolog (S. cerevisiae)
CYP51A1	Cytochrome P450, family 51, subfamily A, polypeptide 1
DYNC1LI1	Dynein, cytoplasmic 1, light intermediate chain 1
EEF1B2	Eukaryotic translation elongation factor 1 beta 2
FABP5	Fatty acid binding protein 5 (psoriasis-associated)
FAM70A	Family with sequence similarity 70, member A
G3BP1	GTPase activating protein (SH3 domain) binding protein 1
GABARAPL2	GABA(A) receptor-associated protein-like 2
GCLC	Glutamate-cysteine ligase, catalytic subunit
GNG10	Guanine nucleotide binding protein (G protein), gamma 10
HCCS	Holocytochrome c synthase
HIAT1	Hippocampus abundant transcript 1
HMGB1	High mobility group box 1
HRSP12	Heat-responsive protein 12
KIAA1033	KIAA1033
KIAA1279	KIAA1279
KPNA2	Karyopherin alpha 2 (RAG cohort 1, importin alpha 1)
LIG4	Ligase IV, DNA, ATP-dependent
LIN7C	Lin-7 homolog C (C. elegans)
LY75	Lymphocyte antigen 75
LYPLA1	Lysophospholipase I
MED21	Mediator complex subunit 21
MPLKIP	M-phase specific PLK1 interacting protein
NDUFA5	NADH dehydrogenase (ubiquinone) 1 alpha subcomplex, 5, 13kDa
PIK3AP1	Phosphoinositide-3-kinase adaptor protein 1
PPIG	Peptidylprolyl isomerase G (cyclophilin G)
PPP1CB	Protein phosphatase 1, catalytic subunit, beta isozyme
PPP1R15B	Protein phosphatase 1, regulatory subunit 15B
PPP2CB	Protein phosphatase 2, catalytic subunit, beta isozyme
PSMC6	Proteasome (prosome, macropain) 26S subunit, ATPase, 6
PTMA	Prothymosin, alpha
RAP1B	RAP1B, member of RAS oncogene family
ROCK1	Rho-associated, coiled-coil containing protein kinase 1
RPLP1	Ribosomal protein, large, P1
SELT	Selenoprotein T precursor
SLC39A8	Solute carrier family 39 (zinc transporter), member 8
SRSF11	Serine/arginine-rich splicing factor 11
SRSF3	Serine/arginine-rich splicing factor 3
SUMO1	SMT3 suppressor of mif two 3 homolog 1 (S. cerevisiae)
TAF9	TAF9 RNA polymerase II, TATA box binding protein (TBP)-associated factor, 32kDa
TANK	TRAF family member-associated NFKB activator
TDP2	tyrosyl-DNA phosphodiesterase 2
TLR1	Toll-like receptor 1
TMEM38B	Transmembrane protein 38B
TOPORS	Topoisomerase I binding, arginine/serine-rich, E3 ubiquitin protein ligase
TSN	Translin
TUBA3D	Tubulin, alpha 3d
UQCRH	Ubiquinol-cytochrome c reductase hinge protein
VPS26A	Vacuolar protein sorting 26 homolog A (S. pombe)
