# Supplementary material for: Pathogen‐induced inflammation is attenuated by the iminosugar MON‐DNJ via modulation of the unfolded protein response
Source: Immunology. 2021 Aug 1;164(3):587–601. doi: 10.1111/imm.13393 (PMC8517592; doi:10.1111/imm.13393)
Supplement: Supplementary file 9 — Table S6 [file IMM-164-587-s006.pdf]

Supplemental Table 6. Genes used to construct network in Figure 7c

Gene Symbol	Gene Name	UPR	Inflammation	Cell Fate
ADA	Adenosine deaminase	Y	Y	Y
ANKRD1	Ankyrin repeat domain 1 (cardiac muscle)	Y	Y	Y
APP	Amyloid beta (A4) precursor protein	Y	Y	Y
**BCL2A1**	**BCL2-related protein A1**	**Y**	**N**	**Y**
**C1D**	**C1D nuclear receptor corepressor**	**N**	**N**	**Y**
CALR	Calreticulin	Y	Y	Y
**CAPZA1**	**Capping protein (actin filament) muscle Z-line, alpha 1**	**Y**	**Y**	**N**
**CAST**	**Calpastatin**	**N**	**N**	**Y**
**CD164**	**CD164 molecule, sialomucin**	**N**	**Y**	**N**
CD38	CD38 molecule	Y	Y	Y
**CTNNB1**	**Catenin (cadherin-associated protein), beta 1, 88kDa**	**Y**	**Y**	**Y**
**CTR9**	**Ctr9, Paf1/RNA polymerase II complex component, homolog (S. cerevisiae)**	**Y**	**Y**	**N**
DDIT4	DNA-damage-inducible transcript 4	Y	Y	Y
DUSP6	Dual specificity phosphatase 6	Y	Y	Y
**GCLC**	**Glutamate-cysteine ligase, catalytic subunit**	**Y**	**N**	**Y**
**GPR183**	**G protein-coupled receptor 183**	**Y**	**Y**	**N**
**HMGB1**	**High mobility group box 1**	**Y**	**Y**	**Y**
HSP90B1	Heat shock protein 90kDa beta (Grp94), member 1	Y	Y	Y
KLF4	Kruppel-like factor 4 (gut)	Y	Y	Y
**LIG4**	**Ligase IV, DNA, ATP-dependent**	**Y**	**Y**	**Y**
**LY75**	**Lymphocyte antigen 75**	**Y**	**Y**	**N**
MSH2	mutS homolog 2, colon cancer, nonpolyposis type 1 (E. coli)	Y	Y	Y
NFKBIA	Nuclear factor of kappa light polypeptide gene enhancer in B-cells inhibitor, alpha	Y	Y	Y
**PIK3AP1**	**Phosphoinositide-3-kinase adaptor protein 1**	**N**	**Y**	**N**
PIM2	Pim-2 oncogene	Y	Y	Y
**PPP1CB**	**Protein phosphatase 1, catalytic subunit, beta isozyme**	**Y**	**N**	**Y**
**PPP2CB**	**Protein phosphatase 2, catalytic subunit, beta isozyme**	**Y**	**N**	**Y**
**PSMC6**	**Proteasome (prosome, macropain) 26S subunit, ATPase, 6**	**Y**	**Y**	**Y**
**ROCK1**	**Rho-associated, coiled-coil containing protein kinase 1**	**Y**	**Y**	**Y**
SERPINE1	Serpin peptidase inhibitor, clade E (nexin, plasminogen activator inhibitor type 1), member 1	Y	Y	Y
**SUMO1**	**SMT3 suppressor of mif two 3 homolog 1 (S. cerevisiae)**	**Y**	**Y**	**Y**
**TAF9**	**TAF9 RNA polymerase II, TATA box binding protein (TBP)-associated factor, 32kDa**	**Y**	**N**	**Y**
**TANK**	**TRAF family member-associated NFKB activator**	**Y**	**Y**	**N**
**TLR1**	**Toll-like receptor 1**	**Y**	**Y**	**N**
TNFRSF4	Tumor necrosis factor receptor superfamily, member 4	Y	Y	Y
TNFSF10	Tumor necrosis factor (ligand) superfamily, member 10	Y	Y	Y
**TOPORS**	**Topoisomerase I binding, arginine/serine-rich, E3 ubiquitin protein ligase**	**Y**	**N**	**Y**
TRIB1	Tribbles homolog 1 (Drosophila)	Y	Y	Y
TRIB3	Tribbles homolog 3 (Drosophila)	Y	Y	Y
ZC3H12A	Zinc finger CCCH-type containing 12A	Y	Y	Y

Bold text indicates transcriptional response pattern indicated by (*) in **Figure 3**
