# Supplementary material for: Pathogen‐induced inflammation is attenuated by the iminosugar MON‐DNJ via modulation of the unfolded protein response
Source: Immunology. 2021 Aug 1;164(3):587–601. doi: 10.1111/imm.13393 (PMC8517592; doi:10.1111/imm.13393)
Supplement: Supplementary file 10 — Table S7 [file IMM-164-587-s002.pdf]

Supplemental Table 7. M*O*N-DNJ regulated genes at 24 hours

Gene Symbol	Gene Name
CRELD1	Cysteine-rich with EGF-like domains 1
CRELD2	Cysteine-rich with EGF-like domains 2
DDIT3	DNA-damage-inducible transcript 3
HSPA5	Heat shock 70kDa protein 5 (glucose-regulated protein, 78kDa)
HYOU1	Hypoxia up-regulated 1
ISOC2	Isochorismatase domain containing 2
MANF	Mesencephalic astrocyte-derived neurotrophic factor
NUCB2	Nucleobindin 2
PDIA4	Protein disulfide isomerase family A, member 4
PDIA6	Protein disulfide isomerase family A, member 6
SDF2L1	Stromal cell-derived factor 2-like 1
SLC39A8	Solute carrier family 39 (zinc transporter), member 8
TMEM50B	Transmembrane protein 50B
